# A crown-group cnidarian from the Ediacaran of Charnwood Forest, UK

**DOI:** 10.1038/s41559-022-01807-x

**Published:** 2022-07-25

**Authors:** F. S. Dunn, C. G. Kenchington, L. A. Parry, J. W. Clark, R. S. Kendall, P. R. Wilby

**Affiliations:** 1 Oxford University Museum of Natural History, University of Oxford, Oxford, UK; 2 Department of Earth Sciences, University of Cambridge, Cambridge, UK; 3 Department of Earth Sciences, University of Oxford, Oxford, UK; 4 School of Biological Sciences, University of Bristol, Bristol, UK; 5 British Geological Survey, Cardiff University, Cardiff, UK; 6 British Geological Survey, Nicker Hill, Keyworth, Nottingham, UK; 7 Department of Geology, University of Leicester, Leicester, UK

**Keywords:** Palaeontology, Phylogenetics

## Abstract

Cnidarians are a disparate and ancient phylum, encompassing corals and jellyfish, and occupy both the pelagic and benthic realms. They have a rich fossil record from the Phanerozoic eon lending insight into the early history of the group but, although cnidarians diverged from other animals in the Precambrian period, their record from the Ediacaran period (635–542 million years ago) is controversial. Here, we describe a new fossil cnidarian—*Auroralumina attenboroughii* gen. et sp. nov.—from the Ediacaran of Charnwood Forest (557–562 million years ago) that shows two bifurcating polyps enclosed in a rigid, polyhedral, organic skeleton with evidence of simple, densely packed tentacles. *Auroralumina* displays a suite of characters allying it to early medusozoans but shows others more typical of Anthozoa. Phylogenetic analyses recover *Auroralumina* as a stem-group medusozoan and, therefore, the oldest crown-group cnidarian. *Auroralumina* demonstrates both the establishment of the crown group of an animal phylum and the fixation of its body plan tens of millions of years before the Cambrian diversification of animal life.

## Abstract (web summary)

A new fossil cnidarian, *Auroralumina attenboroughi*, from the Ediacaran of Charnwood Forest, UK, described as showing mosaic anthozoan and medusozoan characters, is the oldest yet-known crown-group cnidarian.

## Main

**Fig. 1:**
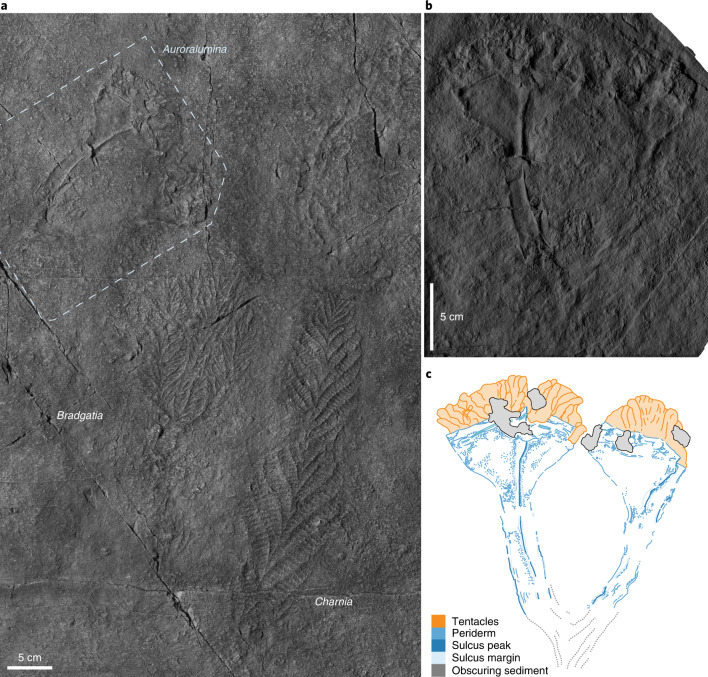
Holotype specimen of *Auroralumina attenboroughii*. **a**, In context alongside rangeomorph fossils preserved in a comparable manner and distinct from the textured background substrate (GSM 105874); imaged under low-angle light. **b**,**c**, Plastotype (GSM 106119) (**b**) and interpretative drawing (**c**) showing the differentiated stalk and cup of each goblet, well-defined corner sulci (now ridges) and texturally distinct tentacles. The proximal portions of both goblets, including their mutual branching point, are concealed beneath a thin cover of sediment but are nonetheless discernible as topographically and texturally distinct tracts (dashed grey line); see Fig. 2 for more information. RTI file available^76^.

The material investigated is a single specimen preserved alongside well-known macrofossils of the Ediacaran period, including *Charnia masoni* and *Bradgatia linfordensis* (Fig. 1). Like them, it consists of a low-profile epirelief impression.

**Horizon and locality.** Bed B (ref. ^1^), Bradgate Formation, Maplewell Group, Charnian Supergroup, Leicestershire, UK; Ediacaran period, 557–562 million years ago (Ma) (ref. ^2^).

Cnidaria Hatschek, 1888

Medusozoa Peterson, 1979

***Auroralumina attenboroughii*** gen. et sp. nov.

**Etymology.** *Aurora* (latin) dawn, referencing the great age of the fossil; *lumina* (latin) light, alluding to the torch-like appearance of the organism; *attenboroughii*, after Sir David Attenborough for his work raising awareness of the Ediacaran fossils of Charnwood Forest.

**Fig. 2:**
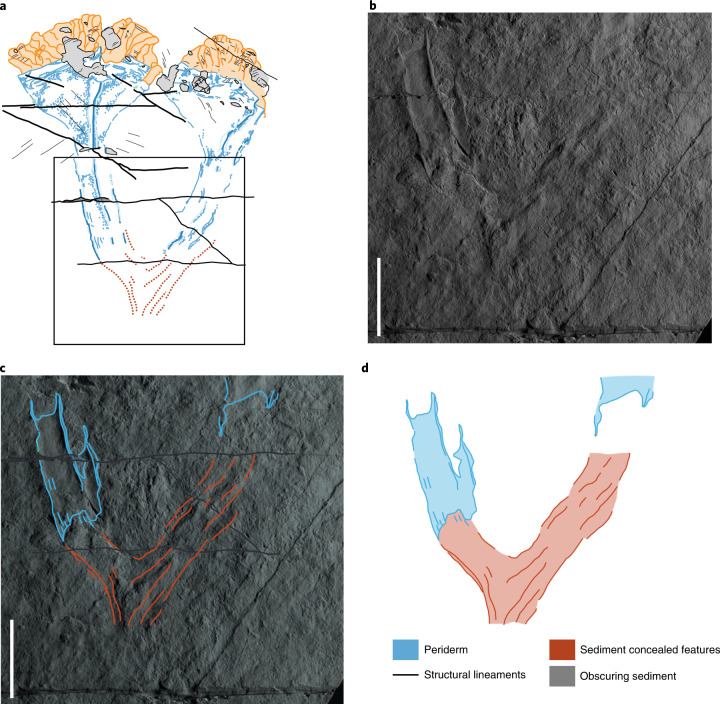
Details of the proximal part of the holotype specimen of *Auroralumina* (GSM 106119). **a**, Interpretative drawing of entire specimen, with area shown in **b**–**d** outlined. **b**, Base of the preserved specimen, showing progressive cover of the left-hand goblet towards the bifurcation point and the mostly concealed proximal part of the right-hand goblet. The margins of the fossil in the concealed area are impressed—albeit weakly—through the sediment and the area underlain by the skeleton is defined by a change in sediment texture. Fossil photographed under low-angle light. **c**, Interpretative overlay, generated by combining observations made under multiple lighting directions. **d**, Interpretative drawing from **c**, showing symmetrical bifurcation of the two goblets and probable broken proximal termination of the specimen. Key in **d** covers all annotations in this figure. Scale bar in **b** and **c**, 5 cm.

**Fig. 3:**
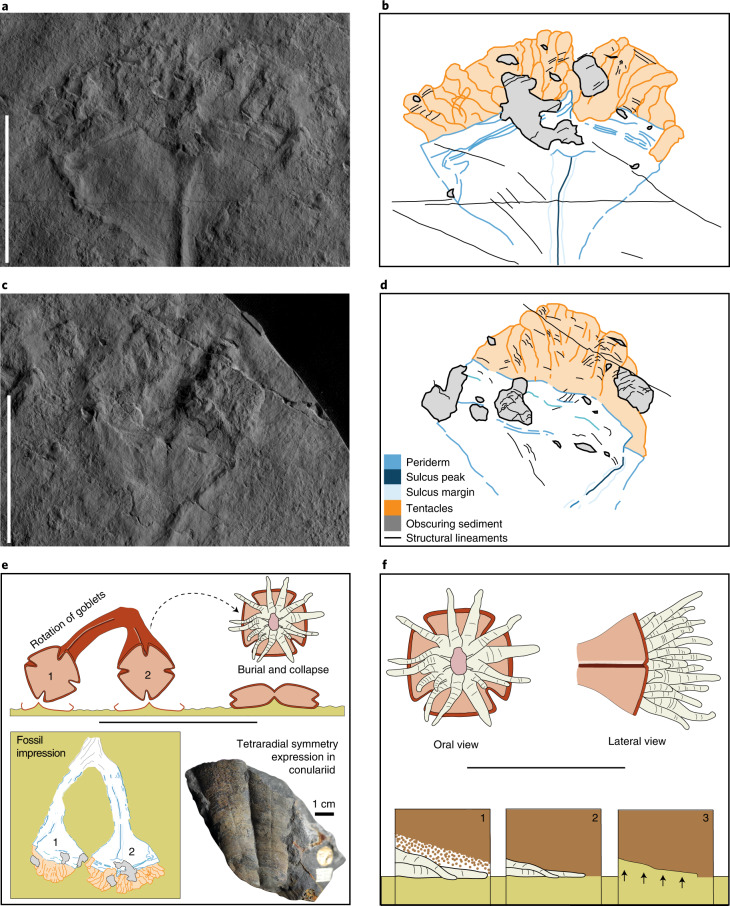
Details of the distal anatomy of *Auroralumina attenboroughii* (GSM 106119) and the mode of preservation. **a**, Left-hand goblet, with dense crown of overlapping tentacles and conspicuous corner sulcus (now a ridge) and band (now a trench) near the aperture rim. The margins of the fossil are well-defined and the tentacle crown texturally and topographically distinct from the smooth periderm. **b**, Interpretative drawing of region in **a**. **c**, Right-hand goblet, principally preserving only one face but with a second partially visible where its edge (and intervening corner sulcus) was twisted into the plane of preservation, towards the right-hand side. **d**, Interpretative drawing of region in **c**. Specimen photographed under low-angle light and interpretations based on features revealed by varying the lighting direction. Scale bar in **a** and **c**, 5 cm. **e**,**f**, Preservation of the goblet and tentacles of *A. attenboroughii*. **e**, Apical view of the two goblets showing how their different orientations at the time of burial generated different views of the tetraradial structure in the fossil in lateral aspect. Schematic goblets (labelled 1 and 2) are representative of the two goblets in *Auroralumina*. The interpretative drawing of *Auroralumina* is also shown, with goblets labelled 1 and 2 next to a conulariid cnidarian (OUMNH DU17), also inferred to have been tetraradial in life, to illustrate analogous preservation of multiple faces in lateral view. **f**, Hypothetical arrangement of the tentacles in oral view in vivo and probable arrangement of tentacles in lateral view at time of burial along with proposed preservational pathway of the tentacles. 1: Tentacles, mostly overlapping, buried by sediment. 2: Partial retraction and deflation postmortem. 3: Decay and casting of the resultant space by sediment from below.

**Holotype.** See Figs. 1–3. The holotype specimen remains in situ in the field; the plastotype is housed at the British Geological Survey, Nottingham (GSM 106119). For the Reflectance Transformation Imaging (RTI) image of the holotype specimen (GSM 106352), see Data availability. These casts were taken from primary mould GSM 105874.

**Diagnosis.** Thecate, medusozoan cnidarian with colonial polypoid phase. Equi-sized, bifurcating polyps are encased in goblet-shaped, organic-walled, periderm with deep-corner sulci imparting a polyhedral outline and a form of radial symmetry but without conspicuous external ornament, excepting a thin concentric band near the aperatural rim (Fig. 1). Periderm divided into two regions: the stalk and the cup. Polyp possesses a dense crown of uniform, unbranched tentacles which extend beyond the aperture of the periderm. Genus diagnosis the same by monotypy.

**Description.** The holotype (Fig. 1) is ~20 cm in total length and is surrounded by a subtle microbial mat fabric that shows no indication of wrinkling or folding (Fig. 1). It comprises two, well-defined, subparallel, goblet-shaped impressions that bifurcate from a less distinct area partially obscured beneath a thin cover of sediment: no detail is preserved proximal of this point (Fig. 2). The goblet-shaped structures are equi-sized and are each constructed of a stalk (~12 cm in length) which abruptly expands into a distinct cup (~6 cm in length). Each goblet has a well-defined linear ridge, running proximodistally, dividing them into two visible faces which, at their maximum, are ~6 cm wide. The left-hand goblet is divided symetrically by the ridge, which runs its entire length up to the apical margin of the cup, whereas the right-hand goblet is asymmetrically bisected.

The apical margin of the cup is defined by a straight rim and by a narrow trench (corresponding to a low ridge in the living organism) that runs parallel to it ~0.8 cm below. No other surface ornament is present. Fringing the apical margin is a dense crown of short (~2.75 cm), apparently uniform and simple projections, each maintaining an approximately constant width and with a blunt termination. These are not contiguous with the apical margins of the cups; instead, they appear to emanate from within them. A minimum of 30, locally overlapping, projections are distinguishable in the better-preserved (left-hand; Fig. 3a,b) cup.

## Taphonomy and interpretation

The fossil is sharply differentiated from the irregularly textured background substrate and, like all other fossils on the surface, only one side of its lateral exterior is preserved. The left-hand goblet outline is symmetrical across the left and right, suggesting that the other side of the goblet was identical and therefore indicating that the goblet was probably tetraradial (Fig. 3e). Preservation of the goblets and the crowns is markedly different (Fig. 1). The goblets are preserved in negative epirelief with raised rims, in common with most other fossils in the assemblage but the rims are notably sharper and higher and the goblet surfaces are smooth (Fig. 1). The central ridges show the greatest relief of any fossilized remains on the surface (Fig. 1). The absence of evidence for deformation, the sharper definition and the higher relief of the fossil relative to other co-occurring taxa (for example, rangeomorphs) all imply that the goblets were constructed of stiffer material. As these are negative epirelief impressions, the relief of structures is in the opposite sense—so in life, the raised structure would have been a trough, separating distinct faces of the goblet as a sulcus. There is no evidence for the former presence of biominerals (for example, brittle fracture or dissolution features), leading us to conclude that the goblets were originally organic-walled. There is no original carbonaceous material remaining in any Ediacaran Charnwood Forest locality. The preservation of two faces separated by a deep sulcus is common in fossil cnidarians, such as conulariids (Fig. 3e) and is a consequence of the compression of a three-dimensional organism onto a two-dimensional surface during burial (Fig. 3e).

The different bisections of the two goblets imply that they are preserved in different orientations. The occurrence of two symmetrical faces in one goblet (left-hand), and of a similarly sized face and partial face in the other (right-hand), is consistent with each goblet presenting a different view of an originally tetraradially symmetrical structure, much as in fossil conulariids and *Carinachites*^3,4^ (Fig. 3e). However, it could also represent a biradial structure as with hexangulaconulariids^5^ or—if the margins of the left-hand goblet do not represent the margins of the periderm faces—triradial symmetry^6^. The preservation cannot be reconciled with pentaradial symmetry, which would require unequal face widths, a condition not currently known in cnidarians with those symmetry states (for example, refs. ^7,8^). We view tetraradial symmetry as most plausible because we consider that the margins of the left-hand goblet probably reflect the margins of faces and note that the maximum width of the largest face in the right-hand goblet is the same as the maximum width of the equi-sized faces in the left-hand goblet. However, we acknowledge uncertainty that might be resolved by discovery of additional specimens. The basal-most part of the specimen, past our inferred bifurcation point, does not align with the orientation of either of the two goblets individually, which supports our interpretation of the goblets as bifurfcating rather than two separate individuals. The obscured and indistinct nature of the most basal point means that we cannot say how much of the original organism is missing—the specimen we have may have been much larger in life, with additional goblets that are absent from our preserved view of the organism.

Unlike the goblets, the crowns are preserved in positive epirelief, recording the upper surface of the organism. They have poorly defined margins and faint wrinkling, recording the combined impressions of multiple overlapping projections (Fig. 3f) as is seen in, for example, multifoliate rangeomorphs^9^ where multiple branches overlap. The specimen is preserved in lateral view—as with all other fossils on the surface—so it is not possible to see the arrangement of this crown axially, on the interior of the goblets. The projections in the crown bear greatest similarity to tentacles of living animals, but are preserved as a compound impression rather than as individual tentacles. They lack external ornament and do not appear to taper.

**Fig. 4:**
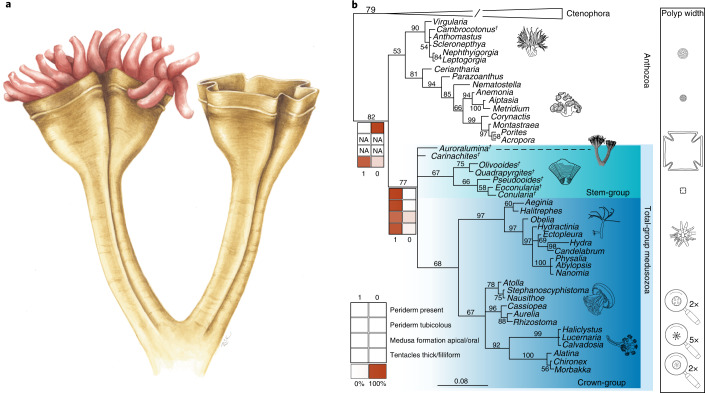
The Phylogenetic position of *Auroralumina attenboroughii*. **a**, Artistic reconstruction of *Auroralumina*. **b**, Bayesian phylogenetic analysis of animals (348 characters, 108 taxa, mk + gamma model) incorporating *Auroralumina attenboroughii*. Numbers indicate posterior probabilities and scale bar shows expected number of substitutions per site. Fossils are indicated by dagger symbols. Raw polyp width is shown on the right, with the mean size shown for the extant groups sampled (for logged polyp size graph, see Extended Data Fig. 3). Branch length shown. Maximum polyp width data also shown in Extended Data Fig. 3. NA indicates where ancestral state values were inapplicable because they were derived from characters recovered as absent.

The combined taphonomic expression of the fossil suggests stark differences in tissue toughness between the two parts, implying that these were originally constructed of different materials: one more rigid than rangeomorph fronds and able to deform the underlying sediment surface (the goblets) and the other sufficiently less resilient than rangeomorph fronds to have had its volume cast by sediment ingressed from below (the crown)^10,11^. We therefore interpret *Auroralumina* as a polypoid cnidarian, with a smooth, resistant, organic-walled periderm encasing a soft polyp that bears unbranched tentacles (Fig. 4a). The combination of a polyhedral organic-walled exoskeleton and corner sulci with associated softer tissues emerging from the aperture is compatible with interpretation of this structure as a cnidarian periderm to the exclusion of other potential structures. The body of the polyp would have been inside the cup in life and so only the protruding tentacles are preserved in this lateral view.

## Phylogenetic position and morphospace occupation

**Extended Data Fig. 1:**
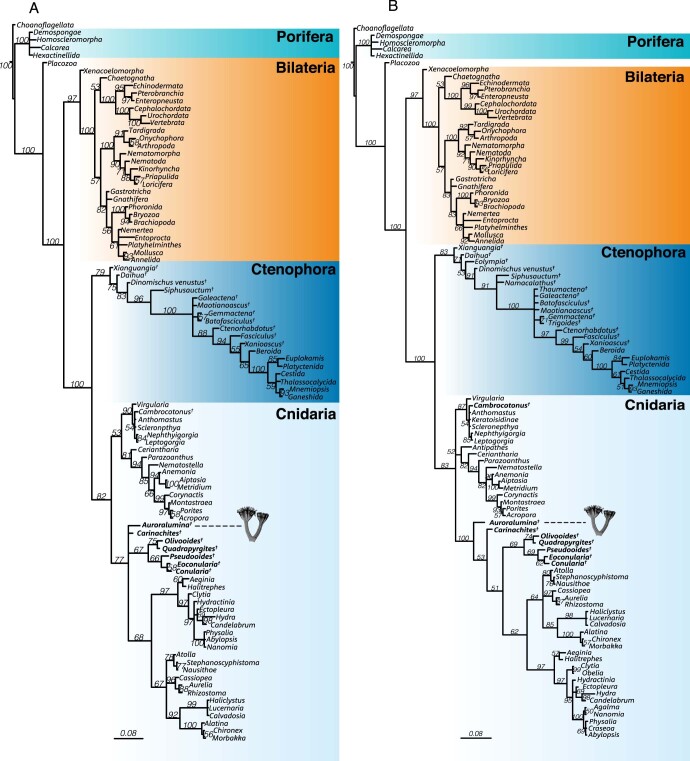
Unconstrained phylogenetic topologies presented in full. (**a**) Excluding the fossil taxa *Namacalathus* and *Eolympia, Antipathes* and those taxa that posses uninformative character states after safe taxonomic reduction (**b**) including all taxa. *Auroralumina* is recovered as a cnidarian in both trees. Fossil cnidarians are shown in bold and the position of *Auroralumina* is highlighted with a silhouette. Scale bar for branch lengths is in units of expected number of substitutions per site.

**Extended Data Fig. 2:**
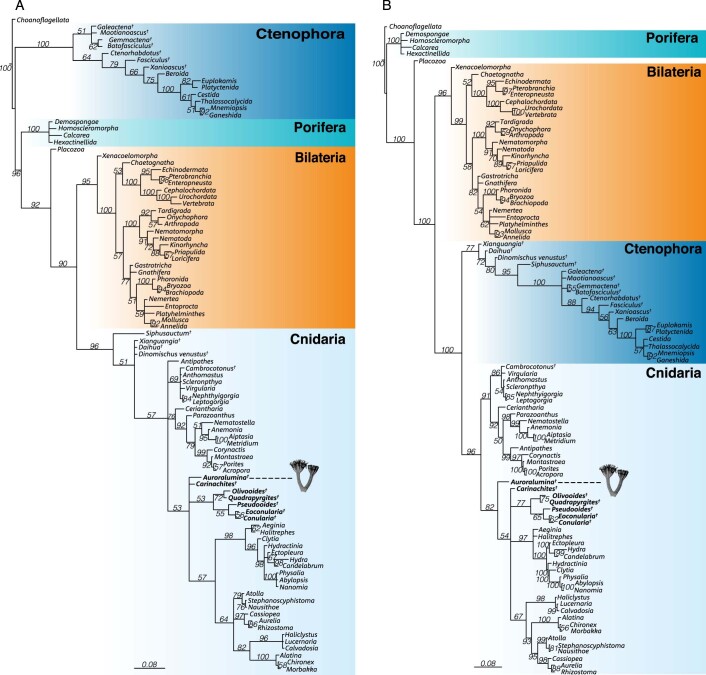
Constrained phylogenetic topologies. (**a**) ‘Ctenosis’ (ctenophores as sister to all other animals) constrained. (**b**) Living cnidarian inter-relationships constrained against recent molecular phylogenies. All fossils were allowed to fully explore treespace under both set of constraints. *Auroralumina* is recovered as a cnidarian in both cases. Fossil cnidarians are shown in bold and the position of *Auroralumina* highted with with a silhouette. Scale bar for branch lengths is in units of expected number of substitutions per site.

Our phylogenetic analysis recovers a topology that agrees with most modern molecular studies (for example, ref. ^12^) and places *Auroralumina* in the medusozoan stem group (Fig. 4b and Extended Data Fig. 1). We recover olivooids, *Pseudooides* and conulariids as stem-group medusozoans, which differs from recent analyses that have resolved them as crown-group scyphozoans (for example, ref. ^13^). Together, these data reconstruct the medusozoan ancestor as being broadly scyphozoan-like, with a polyp-encasing periderm (Fig. 4b). Our results are stable when ctenophores are constrained as the sister to all other animals (Extended Data Fig. 2a) and when the specific inter-relationships of the Cnidaria are fixed to match recent molecular phylogenies (Extended Data Fig. 2b).

We investigated morphospace occupation of tubular fossils (those with an external tubular skeleton within which an animal resided) across the Ediacaran–Cambrian transition as a mechanism to determine whether *Auroralumina* is significantly different from other Ediacaran tubular fossils and whether it is more similar to those fossils found in the early Cambrian period. As disparity analyses are phylogenetically independent, we incorporated a large suite of Ediacaran tubular taxa including those that are controversial and may or may not represent ancient cnidarians. The disparity matrix used in our analyses was based on characters published previously (refs. ^14,15^ and other publications; see Supplementary Data 2 for a full list) which compared various phenotypic features of tubular, exoskeletal fossils across the Ediacaran and early Cambrian periods.

**Fig. 5:**
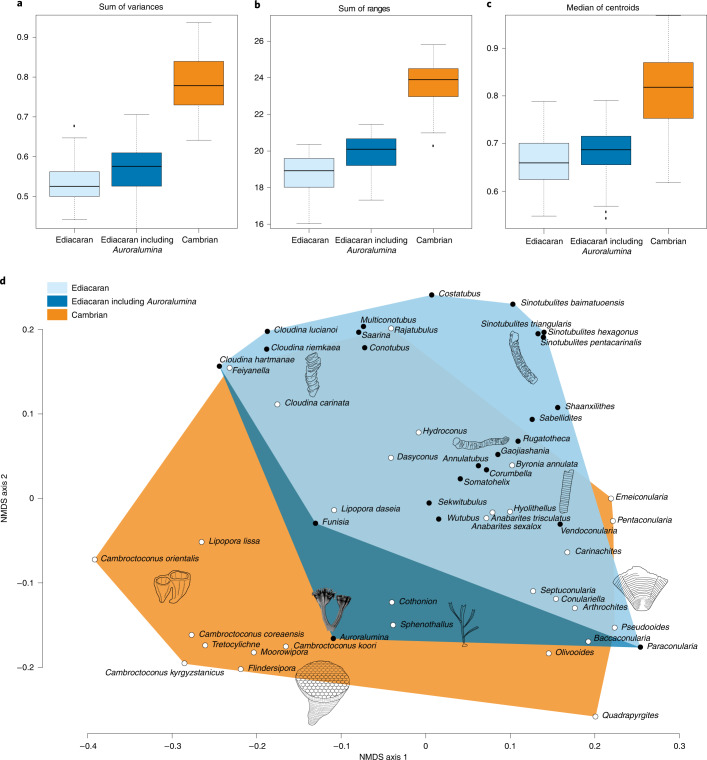
Tubular morphospace occupation across the Ediacaran–Cambrian transition. **a**–**c**, The sum of variances (**a**), sum of ranges (**b**) and the median of centroids (**c**) for tubular morphospace occupation. The sum of variances examines the evenness of morphospace occupation, the sum of ranges examines the extent of morphospace occupation in all computed dimensions and the median of centroids measures the clustering of taxa around a central point. Adding *Auroralumina* increases the sum of variances, ranges and (marginally) the median of centroids compared to Ediacaran morphospace excluding *Auroralumina*. The boxes represent the interquartile range, with black line showing the median. The whiskers indicate minimum (Q1 − 1.5 × IQR) and maximum (Q3 + 1.5 × IQR), excluding outliers. Outliers are shown in black squares. **d**, Morphospace occupation with convex hulls showing Ediacaran morphospace occupation with and without *Auroralumina* and Cambrian morphospace occupation. Black circles represent Ediacaran taxa and white circles represent Cambrian taxa.

Calculating the non-metric multidimensional scaling (NMDS) with four axes produced a fair fit (stress value <0.1), so was used as the basis for further analysis. Inclusion of *Auroralumina* in the Ediacaran tube morphospace increased all aspects of disparity measured here (Fig. 5).

*Auroralumina* has a major impact on the extent of Ediacaran tube morphospace and brings the Ediacaran tube morphospace closer in position and size to that of the Cambrian. The variance and extent of tubular morphospace occupation is comparatively low in the Ediacaran, indicating that tubular anatomies were not highly distinct, despite an increase in the abundance of tube-forming group(s) at this time^14^. *Auroralumina*’s location in the morphospace confirms that its anatomy is distinct from all other known Ediacaran tubular fossils and it is nested within Cambrian cnidarians, between presumed anthozoan and medusozoan taxa. Overall, morphospace variance increases into the Cambrian for all metrics we analysed, as tubular body fossils become more distinct and disparate and the distinctive Ediacaran nested tube-in-tube morphology^14^ declines. Analysis of variance of disparity by group shows that the morphospace occupied by Ediacaran tubular taxa without *Auroralumina* is significantly different to the Cambrian morphospace (*R*^2^ Pr(>*F*) < 0.001) but, when *Auroralumina* is added, the Ediacaran and Cambrian morphospaces are not stastistically distinguishable (*R*^2^ Pr(>*F*) = 0.586), while the Ediacaran without *Auroralumina* is significantly different from Ediacaran with *Auroralumina* (*R*^2^ Pr(>*F*) = 0.045). This further supports the greater similarity of *Auroralumina* to Cambrian rather than other Ediacaran taxa.

## Discussion

Molecular clocks recover a Precambrian divergence between the cnidarian lineages, with subsequent radiations through the early Palaeozoic^16,17^ and there have been many claims of Ediacaran fossils with cnidarian affinity, perhaps most notably *Haootia, Corumbella* and *Cloudina*.

*Haootia quadriformis* is a polypoid organism from the Fermeuse Formation of the Bonavisata Peninsula, Newfoundland, that has been described as a total-group cnidarian^18^ but has been broadly discussed as a stem-group medusozoan or as staurozoan-like, primarily based on the presence of interpreted tetradial symmetry and an open calyx. A stem-group medusozoan hypothesis for *Haootia* is contingent on the placement of Staurozoa as sister to all other medusozoans and their anatomy consequently being plesiomorphic for the group as a whole. However, this topology has not been recovered by recent phylogenies of either molecular or morphological data^12,19^ nor is it recovered here. This means that it is likely that their medusan anatomy—the major life stage—is derived and does not represent the plesiomorphic condition for Medusozoa. While a staurozoan affinity has never been formally proposed, clear similarities in the reconstructed muscle anatomy of *Haootia* and stauromedusans have invited comparison. However, the arrangement of muscle fibres is contested^20,21^ and the currently described muscle arrangement is not compatible with the feeding mode of known staurozoans or any extant cnidarian. Because of confusion over the life history stage of *Haootia*, we elected not to include it in our matrix.

There are also a number of latest Ediacaran skeletal fossils that are potential early cnidarians*. Corumbella wernerii* was likened to the conulariids on the basis of an externally annulated skeleton and tetraradial symmetry^22^. However, more recent work on three-dimensional specimens from the Tamengo Formation, Brazil^23^, has challenged this, arguing that *Corumbella* is circular in outline and lacks a pyramidal shape, carinae, straight facets or corner grooves, all of which are incompatible with a conulariid affinity. Previous authors^23^ go on to suggest that the surface ornamentation of *Corumbella* is much more like another Ediacaran genus, *Sinotubulites*. However, the annulated, elongate, tapering tube (with approximately circular cross-section) of *Corumbella* is compatible with a cnidarian affinity and does bear notable similarity to extant coronate scyphozoan dwelling tubes^24^.

The late Ediacaran genus *Cloudina* is one of a number of Ediacaran tubular taxa that possess a distinctive funnel-in-funnel morphology. The affinities of *Cloudina* and similar taxa are controversial, with some authors arguing for an annelid affinity while others compare them with non-bilaterians, chiefly cnidarians. Proponents of an annelid affinity for *Cloudina* have argued that the putative presence of direct development excludes a placement in Cnidaria^25^; however, there are several Cambrian, skeletonizing fossil cnidarian taxa known to undergo direct development (see below). Furthermore, the annelids with which *Cloudina* has been closely compared (Serpulidae^26^ and Siboglinidae^25^) both go through indirect development via a trochophore larva^27,28^, a feature common to many marine annelids and their close relatives. The tube microstructures in *Cloudina* that are comparable with those of annelids have evolved many times (for example, in Alvinellidae and Siboglinidae^25^), while the granular tube microstructure of *Cloudina* is found in living cnidarians but is absent in calcareous tube-forming annelids^29^, along with polytomous branching^30^, a lack of attachment structures and a closed tube base (except in individuals that have undergone damage)^29^. Further evidence for a total-group bilaterian affinity was provided by the discovery of fossilized soft tissues, interpreted as a through gut^31^. The proposed gut morphology was used as evidence against a cnidarian affinity due to the absence of features characteristic of anthozoans, such as an actinopharynx, and longitudinal septa are also absent from the skeleton. However, these features are not present in medusozoan polyps^32^ with many medusozoans having a gut gross morphology that is broadly comparable with that observed in the soft tissues of cloudinomomorphs. Furthermore, there are a variety of annelid-mimicking bilaterian groups known from the Palaeozoic era, although these mostly first appear from the Ordovician period onwards^33^. While recent discoveries have provided critical insights into the tube ultrastructure, growth and soft-tissue structures of cloudiniids, placing *Cloudina* in the total group of any animal phylum may be premature and we chose not to consider it in our phylogenetic analysis.

Embryonic and post-embryonic fossils from the earliest Cambrian, alongside small shelly fossil remains, provide the most compelling evidence for a diverse cnidarian fauna by this time and are the most character-rich of any proposed cnidarian fossils so far covered here. These fossils are likely to represent at least a grade of organization and are sometimes considered a clade, sister to the coronate Scyphozoa. *Olivooides*^8^ and *Quadrapyrgites*^34^ possess angular, ornamented periderm and an aperture constructed of subtriangular lappets. Intriguingly, stacked pentameral ephyrae have been found in association with *Olivooides*, suggesting that it underwent polydisc strobilation and probably produced medusae. Similarly, hexanguloconulariids are direct developing polypoid organisms^13^ that possess a tubular periderm with angular faces, different levels of external ornamentation and peridermal teeth/ridges but they lack the apertural lobes of olivooids. *Carinachites*^3^ possesses an *Olivooides-*like apical aperture but, where the periderm of olivooids and hexangulaconulariids is marked by angular faces, *Carinachites* is marked by deep-corner sulci, much like the conulariids. In addition, conulariids themselves may possess apertural lobes and peridermal teeth/ridges^4,35^. There is some evidence to suggest that conulariids may have undergone strobilation^36^ but this is contested^37^. Unlike olivooids, carincachitids and hexangulaconulariids, conulariids possess majority tetraradial symmetry. Conulariids are the longest-ranging of these groups, extending from the latest Ediacaran period (with the occurrence of *Paraconularia* in the Tamengo Formation) to the Late Triassic period (for example, ref. ^4^). Much has been made of the similarities not just between olivooids, hexangulaconulariids, carinachitids and conulariids but between these fossil groups and extant coronate scyphozoans (for example, ref. ^38^). There are, however, several differences between these Palaeozoic fossil groups and living scyphozoan polyps. The fossil groups can exhibit deep-corner septa in their periderm, or at least the periderm is constructed of smooth faces and the ornamentation across these polyps is regular. This is unlike scyphozoan polyps, which exhibit a smooth, conical periderm, often with irregular ornamentation^24,39^. Furthermore, scyphozoan ephyrae present a number of characters, including velar and rhopalial lappets^40^, which are lacking in the ephyrae of *Olivooides*. These characters render the morphology of the *Olivooides* ephyrae unique among medusozoans. Additionally, the interpreted direct development in many of these fossils is unlike living scyphozoans^8,13^.

*Auroralumina* presents a suite of characters in common with these early Cambrian forms: deep-corner sulci and a polyhedral, probably tetraradial, tubicolous periderm. However, unlike these other groups, the periderm is not ornamented and is not tapering: it is split into two distinct regions, the stalk and the cup. However, the fossil is incomplete, raising the possibility that *Auroralumina* may only have been annulated over the most proximal part of its body. This is a condition observed in some living hydrozoans^41^, where annulation is often less prevalent^42,43^ and where tubicolous periderm can additionally form a stalk and cup^32^. However, a non-annulated periderm also warrants comparison with the skeleton of certain anthozoan cnidarians including cerianthids, anemones and certain octocorals. Cerianthids produce organic tubes with adhered sediment grains. These tubes are circular in outline and can show either irregular concentric annulations or longitudinal striations. This is not compatible with the anatomy of *Auroralumina* which is polyhedral in outline, has no evidence of adhered sediment grains (evidenced by the smooth nature of the periderm) and is additionally fundamentally inconsistent with burrowing. Furthermore, the presence of corner sulci is distinct from the longitudinal striations seen in cerianthids^44^. Some anthozoans, including the anemone *Edwardsiella*^45^ and the octocoral genus *Cornularia*^46^, are described as exhibiting periderm. In anemones, this is circular in outline and restricted to one region in the midpolyp (Scarpus), where it shows an irregular outline^47^ and is therefore not comparable to the condition in *Auroralumina*. The octocoral *Cornularia* has been described as showing periderm and was recently recovered as the sister to all other octocorals^46^ but this condition is unique amongst octocorals, requiring it to be lost in all other octocorals and hexacorals to be plesiomorphic for anthozoans; this is a scenario we consider unlikely. Rather, members of the polyphyletic grouping Stolonifera, to which *Cornularia* belongs, show differing levels of thickening surrounding the anthostele—the basal part of the polyp which is connected to other polyps in the colony—and around the stolons^48^. It is possible that the periderm of *Cornularia* represents a derived condition within the group, derived from the chitinous covering of the stolons which, as the plesiomorphic condition of crown-group anthozoans, medusozoans or cnidarians, is not inferred to have been colonial^49^. On the basis of these data, we consider it most likely that the periderm of some anthozoans is the result of convergence, rather than shared ancestry with medusozoan periderm, and we argue that *Auroralumina* exhibits a periderm that is homologous with those of medusozoan cnidarians given the additional shared characters.

**Extended Data Fig. 3:**
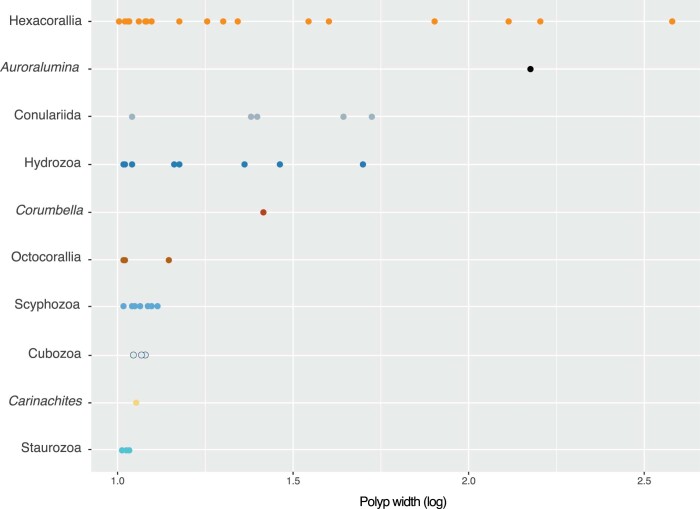
Maximum polyp width in cnidarians. Maximum polyp width plotted for extant cnidarian Classes and fossil groups. *Auroralumina* has a much larger polyp width than any other sampled medusozoan. Maximum conulariid polyp width is also larger than any sampled living medusozoan. Source data available with manuscript.

Living members of Staurozoa, Cubozoa and Scyphozoa, which all produce medusae via transformation of their polyp apex, possess polyps that are an order of magnitude smaller than those of *Auroralumina* (Fig. 4b and Extended Data Fig. 3). Hydrozoan polyps show far greater variation but do not all produce medusae; those that do produce medusae use a strategy of budding laterally via an entocodon, derived as per our ancestral state reconstructions (Fig. 4b). The small polyp size in living scyphozoans, staurozoans and cubozoans (Fig. 4b and Extended Data Fig. 3) could be the result of close phylogenetic relationship but it may also be a feature of their medusa-formation strategy. Living hydrozoans are not so constrained (though they are still smaller than *Auroralumina*), despite close phylogenetic relationship, and do not use a strategy that is so obviously contingent on polyp apex size. It is, at present, impossible to say when the medusa stage evolved along the medusozoan stem and it is not known whether there is a maximum polyp size after which medusa formation is impossible. However, the large size of *Auroralumina* in comparison to living medusozoans and our reconstruction of apical/oral medusa formation as plesiomorphic for the group, does raise the possibility that *Auroralumina* was not able to produce medusae. Refinement of the phylogenetic inter-relationships of stem-group medusozoans may shed further light on the position of *Auroralumina* in the stem and on the timing of the evolution of medusa formation, which is currently constrained as minimally earliest Cambrian^50^.

*Auroralumina* displays a distinctive combination of characters that is not present in other fossil taxa and which helps to better resolve the phylogenetic affinities of several extinct medusozoans, shedding light on the early evolution of a number of key cnidarian traits. We infer the presence of a tubicolous periderm in the ancestor of both the total-group and crown-group Medusozoa, implying its independent loss/reduction in living staurozoans, cubozoans and some hydrozoans (Fig. 4b) and, together, our data suggest a scyphozoan-like ancestor for the crown group of Medusozoa. Additionally, *Auroralumina* has the greatest polyp width of any medusozoan we are aware of (Fig. 4b and Extended Data Fig. 3) and is at least an order of magnitude larger than living staurozoans, cubozoans and scyphozoans. There is substantial variability in the size of hydrozoan polyps, with some solitary polyps (for example, *Branchiocerianthus imperator*^51^) reaching metre-scale sizes but these are outliers from the largely colonial and miniature group. Conulariid and *Corumbella* polyp widths are also larger than any living member of these groups, suggesting that small polyp size is a feature of the crown group of the medusozoan lineages. *Auroralumina* possessed a polyhedral, probably tetraradial periderm with deep-corner sulci, allying it with other fossil medusozoans. A tubicolous, sulcate periderm clearly differentiates early medusozoans from early anthozoans, which we infer to have been naked, anemone-like, polyps (sensu^49^). A naked anemone-like polyp would have a reduced preservational potential under most settings as compared to a peridermal polyp^52^, perhaps going some way to explain the preponderance of stem-group medusozoans in the fossil record to the exclusion of clear stem-group anthozoans.

*Auroralumina* confirms the presence of crown-group cnidarians coeval with the oldest assemblage of the Ediacaran macrobiota and is the most ancient fossil that can be reliably ascribed to the crown group of any living animal phylum. Living cnidarians (other than derived, parasitic groups) use their tentacles to catch food and the presence of a dense tentacular crown would support a similar life-habit for *Auroralumina*. *Auroralumina* may have fed on diversifying phytoplankton^53^ or protists^54^ but, additionally, may have consumed an emerging zooplankton. The presence of a stem-group medusozoan necessitates the presence of other cnidarians, as well as other early-diverging animal lineages known to have planktonic phases in their life cycle (poriferans and ctenophores). Additionally, the cosmopolitan distribution of rangeomorph taxa has been used to infer the presence of a water-borne planktonic propagular stage^55^ on which *Auroralumina* may have fed.

*Auroralumina* is a thecate cnidarian with a polyhedrally symmetrical periderm and extends the fossil record of crown-group cnidarians by ~25 million years, deep into the Ediacaran period. Animal body plans are widely assumed to have become fixed during the Cambrian Explosion but *Auroralumina* demonstrates that at least one crown-group cnidarian body plan had already been established tens of millions of years previously.

## Methods

The specimen was imaged at the British Geological Survey using RTI^56^. Images were derived either from the RTI or from photography using low-angled light.

We analysed our morphological data matrix in MrBayes 3.2.7, under the Mki with gamma model, with correction for use of only informative characters. Our matrix was based on a previously published dataset^13,19,57^ which was expanded to include additional taxa and fossil organisms. The R package Claddis was used to perform safe taxonomic reduction and prune uninformative taxa^58^. Analyses were set to run for 20 million generations, with a requested stop value meaning that the analyses stopped when the deviation of split frequencies dropped below 0.01. Convergence was assessed by checking that the effective sample size was >200 and that the potential scale-reduction factor was ~1. Our topology recovers a monophyletic Anthozoa and reciprocal monophyly of the two scyphozoan clades in a polytomy with Cubozoa and Scyphozoa (which are monophyletic). This tree differs in some ways from recent molecular phylogenies. Therefore, we constrained the lineages in our tree to conform to molecular results^12,49^ but allowed all fossils to wander. Additionally, we constrained ctenophores as sister-group to all other animals^59^ to test the sensitivity of our results to recovering a monophyletic Coelenterata. Finally, we performed ancestral state reconstructions incorporating phylogenetic uncertainty on characters of interest and these were also assessed for convergence using the above parameters. See Supplementary Information for full description.

All disparity analyses were performed in R. The distance matrix used in the disparity analyses was computed using Gower’s similarity metric, as this allows for handling of nominal, ordinal, multistate and (a)symmetric binary data^60^. The multidimensional space was then constructed using NMDS, through the vegan package^61^. NMDS is a non-eigenvector-based multivariate method that does not assume linear relationships between the variables and allows for large amounts of missing data. The number of axes used in the calculation of the multidimensional space was determined through visual assessment of a stress scree plot and calculation of the stress values for set numbers of axes. Stress values of <0.1 were taken as a good fit and <0.05 as an excellent fit, following ref. ^62^. The data were then analysed using the dispRity package^63^. This package allows for useful visualization of the morphospace, as well as allowing the user to define the metrics by which to analyse the data. It also includes functionality for analysing subsets of the data within the morphospace and for non-parametric analysis of variance (NPMANOVA). Several metrics were used herein to gain a complete picture of how disparity changes through time (following ref. ^64^). Each metric was run on a bootstrapped matrix output for each data subset. For details on disparity matrix construction, see Supplementary Information.

We expanded that dataset to include *Auroralumina* and a number of additional Cambrian genera with tubular skeletons that are inferred to represent ancient cnidarians: *Byronia*^65^, *Sphenothallus*^66^, *Olivooides*^8^, *Quadrapyrgites*^34^, *Pseudooides*^13^, *Arthrochites*^67^, *Carinachites*^3^, *Septaconularia*^67^, *Anabarites*^68^, *Conulariella*^4,69^, *Paraconularia*^38^ and *Baccaconularia*^4,37^. We also separated the genus *Cloudina* and scored two individual species—*Cloudina carinata*^70^ and *Cloudina hartmannae*^71^—but did not include any additional species because distinguishing characters were too specific for the purposes of this study (assessed in ref. ^72^). We also separated the genus *Sinotubulites* into four individual species—*S. triangularis, S. pentacarinalis* and *S. hexagonus* from ref. ^7^, alongside *S. baimatuoensis*^7^. Additionally, we rescored the symmetry state of *Corumbella* from ref. ^14^ after ref. ^23^, which reports new data suggesting a circular cross-section and not a polyhedral cross-section. Finally, we added taxa forming the CLT clade of Park et al.^15^ and additional corralomorph taxa (see Supplementary Information).

Our list of included Cambrian tubular fossils is by no means exhaustive but we have included major early Cambrian tubular fossils that are of proposed cnidarian affinity. Our list of included Ediacaran tubular fossils after ref. ^14^, does include all described forms to at least genus level.

The sum of variance metric calculates the sum of variance of each computed axis of each matrix subset—higher variance indicates less even occupation of the matrix^64,73^. Adding *Auroralumina* increases both the mean and interquartile range (IQR) of the sum of variances of each axis occupied by the morphospace but these are still lower than that of the Cambrian tube morphospace. There is overlap between the IQRs of the Ediacaran and the Ediacaran including *Auroralumina* and between the IQRs of the Ediacaran including *Auroralumina* and the Cambrian. Adding *Auroralumina* means that the Ediacaran morphospace is occupied less evenly but not as unevenly as the Cambrian morphospace. This is also clear from the NMDS plot—all Ediacaran tubes excluding *Auroralumina* plot in the same region of space, while *Auroralumina* occupies a different area on the NMDS axis 2. In the Cambrian morphospace, each group occupies a distinct region of the space, with *Cothonion* and *Sphenothallus* close to *Auroralumina* in this elevation, with most Cambrian groups showing high positive values of NMDS axis 2 but with members occupying the full span of NMDS axis 1 (Fig. 5d).

The sum of ranges calculates the ranges of each axis occupied by the matrix subset and indicates the extent of the morphospace occupied by the subset^64,73^. Adding *Auroralumina* increases the mean sum of ranges of the Ediacaran tube morphospace to above that of the Cambrian tube morphospace (though IQRs of the Cambrian and Ediacaran including *Auroralumina* overlap). Again, this is also apparent from the NMDS plot, given that the position of *Auroralumina* lies far from the main occupation of Ediacaran tube morphospace (Fig. 5).

The median of centroids calculates the median of the distances between each row in the matrix (taxa) and the centroid of the matrix or subset—this indicates how tightly species are clustered around the centroid^74^. Adding *Auroralumina* slightly increases the mean median of the Ediacaran tube morphospace centroids, meaning that species are slightly less clustered around the centroid. This makes sense again given the position of *Auroralumina* far from the main group. In Cambrian morphospace, individuals are more clustered around the centroid—this is evident again from the NMDS plot as Cambrian taxa occur across the morphospace. The wider IQR of Cambrian morphospace is because the corners of the morphospace are in places occupied by only one or two taxa (for example, removal of *Feiyanella*, *Cambroctoconus orientalis* or *Quadrapyrgites* would all serve to contract the morphospace and shift the position of the centroid; Fig. 5).

NPMANOVA analyses can test the null hypothesis that the centroids and dispersions of groups are equal^75^. When conducted on these three subsets, the null hypothesis was statistically rejected for Cambrian versus Ediacaran (*P* = 0.45) and for Cambrian versus Ediacaran including *Auroralumina* versus Ediacaran subsets (*P* = 0.001). The null hypothesis was not rejected for Cambrian versus Ediacaran including *Auroralumina* (*P* = 0.75).

### Reporting summary

Further information on research design is available in the Nature Research Reporting Summary linked to this article.

## Supplementary information

Supplementary Information Supplementary discussion including methods and character information on the morphospace and phylogenetic analyses presented in the main text.

Reporting Summary

Supplementary Data 1 Nexus file containing morphological phylogenetic dataset.

Supplementary Data 2 CSV file with tube morphospace dataset and polyp size data.

Supplementary Data 3 R script used to analyse tube morphospace.

## Data Availability

All data analysed in this paper are available as part of the article, Extended Data Figs. 1–3 or Supplementary Data 1, 2 and 3. An RTI of the fossil is available at 10.5285/4c2f9f34-184d-43db-97e0-eecb13918375 (ref. ^76^). This published work and the nomenclatural acts it contains have been registered in ZooBank, the proposed online registration system for the International Code of Zoological Nomenclature. The ZooBank LSIDs (Life Science Identifiers) can be resolved and the associated information viewed through any standard web browser by appending the LSID to the prefix ‘http://zoobank.org/’. The LSIDs for this publication are: urn:lsid:zoobank.org:act:869AF2A2-FB6B-44DF-88A1-D266B3D101F4

## Code availability

The phylogenetic dataset, commands and constraints needed to run all analyses are included as Supplementary Data 1, 2 and 3 or part of the Supplementary Information. The data required to run the tube morphospace disparity analyses and the polyp size data are included in tabular form as Supplementary Data 1 and 2. The R code required to run the tube morphospace disparity analyses is included as a Supplementary Data 3.

## References

[1] Wilby PR, Carney JN, Howe MP (2011). A rich Ediacaran assemblage from eastern Avalonia: evidence of early widespread diversity in the deep ocean. Geology.

[2] Noble S (2015). Age and global context of the Ediacaran fossils of Charnwood Forest, Leicestershire, UK. Geol. Soc. Am. Bull..

[3] Han J (2018). *Olivooides*-like tube aperture in early Cambrian carinachitids (Medusozoa, Cnidaria). J. Paleontol..

[4] De Moraes Leme J, Guimarães Simões M, Carlos Marques A, Van Iten H (2008). Cladistic analysis of the suborder Conulariina Miller and Gurley, 1896 (Cnidaria, Scyphozoa; Vendian–Triassic). Palaeontology.

[5] Morris SC, Menge C (1992). Carinachitids, hexangulaconulariids, and Punctatus: problematic metazoans from the Early Cambrian of South China. J. Paleontol..

[6] Kouchinsky A, Bengtson S, Feng W, Kutygin R, Val'kov A (2009). The Lower Cambrian fossil anabaritids: affinities, occurrences and systematics. J. Syst. Palaeontol..

[7] Cai Y, Xiao S, Hua H, Yuan X (2015). New material of the biomineralizing tubular fossil *Sinotubulites* from the late Ediacaran Dengying Formation, South China. Precambrian Res..

[8] Dong X-P (2013). Embryos, polyps and medusae of the Early Cambrian scyphozoan *Olivooides*. Proc. R. Soc. B.

[9] Kenchington CG, Dunn FS, Wilby PR (2018). Modularity and overcompensatory growth in Ediacaran rangeomorphs demonstrate early adaptations for coping with environmental pressures. Curr. Biol..

[10] Gehling JG (1999). Microbial mats in terminal Proterozoic siliciclastics; Ediacaran death masks. Palaios.

[11] Kenchington, C. & Wilby, P. R. *Of Time and Taphonomy: Preservation in the Ediacaran* (Geological Society of America, 2014).

[12] Zapata, F. et al. Phylogenomic analyses support traditional relationships within Cnidaria. *PLoS ONE* **10**, e0139068 (2015). 10.1371/journal.pone.0139068 PMC4605497 26465609

[13] Duan B (2017). The early Cambrian fossil embryo *Pseudooides* is a direct-developing cnidarian, not an early ecdysozoan. Proc. R. Soc. B.

[14] Selly T (2020). A new cloudinid fossil assemblage from the terminal Ediacaran of Nevada, USA. J. Syst. Palaeontol..

[15] Park T-YS (2021). Enduring evolutionary embellishment of cloudinids in the Cambrian. R. Soc. Open Sci..

[16] dos Reis M (2015). Uncertainty in the timing of origin of animals and the limits of precision in molecular timescales. Curr. Biol..

[17] Park, T.-y. et al. A stem-group cnidarian described from the mid-Cambrian of China and its significance for cnidarian evolution. *Nat. Commun.* **2**, 442 (2011). 10.1038/ncomms1457 PMC3265377 21863009

[18] Liu, A. G., Matthews, J. J., Menon, L. R., McIlroy, D. & Brasier, M. D. *Haootia quadriformis* n. gen., n. sp., interpreted as a muscular cnidarian impression from the Late Ediacaran period (approx. 560 Ma). *Proc. R. Soc. B*10.1098/rspb.2014.1202 (2014). 10.1098/rspb.2014.1202 PMC4173675 25165764

[19] Zhao Y (2019). Cambrian sessile, suspension feeding stem-group ctenophores and evolution of the comb jelly body plan. Curr. Biol..

[20] Miranda L, Collins A, Marques A (2015). Is *Haootia quadriformis* related to extant Staurozoa (Cnidaria)? Evidence from the muscular system reconsidered. Proc. R. Soc. B.

[21] Liu AG, Matthews JJ, Menon LR, McIlroy D, Brasier MD (2015). The arrangement of possible muscle fibres in the Ediacaran taxon *Haootia quadriformis*. Proc. R. Soc. B.

[22] Pacheco MLF (2015). Insights into the skeletonization, lifestyle, and affinity of the unusual Ediacaran fossil *Corumbella*. PLoS ONE.

[23] Walde DH-G, Weber B, Erdtmann B-D, Steiner M (2019). Taphonomy of *Corumbella*
*werneri* from the Ediacaran of Brazil: sinotubulitid tube or conulariid test?. Alcheringa Australas. J. Palaeontol..

[24] Morandini A (2010). Identification of coronate polyps from the Arctic Ocean: *Nausithoe werneri* Jarms, 1990 (Cnidaria, Scyphozoa, Coronatae), with notes on its biology. Steenstrupia.

[25] Yang, B. et al. Ultrastructure of Ediacaran cloudinids suggests diverse taphonomic histories and affinities with non-biomineralized annelids. *Sci. Rep.* **10**, 535 (2020). 10.1038/s41598-019-56317-x PMC6968996 31953458

[26] Hua H, Chen Z, Yuan X, Zhang L, Xiao S (2005). Skeletogenesis and asexual reproduction in the earliest biomineralizing animal *Cloudina*. Geology.

[27] Bright M, Eichinger I, von Salvini-Plawen L (2013). The metatrochophore of a deep-sea hydrothermal vent vestimentiferan (Polychaeta: Siboglinidae). Org. Divers. Evol..

[28] Rouse GW (2000). Bias? What bias? The evolution of downstream larval‐feeding in animals. Zoologica Scr..

[29] Vinn, O. & Zaton, M. Inconsistencies in proposed annelid affinities of early biomineralized organism *Cloudina* (Ediacaran): structural and ontogenetic evidences. *Carnets Géol.* **3**, 39–47 (2012).

[30] Shore A, Wood R, Curtis A, Bowyer F (2020). Multiple branching and attachment structures in cloudinomorphs, Nama Group, Namibia. Geology.

[31] Schiffbauer, J. D. et al. Discovery of bilaterian-type through-guts in cloudinomorphs from the terminal Ediacaran Period. *Nat. Commun.* **11**, 205 (2020). 10.1038/s41467-019-13882-z PMC6954273 31924764

[32] Hyman, L. H. *Protozoa through Ctenophora* (McGraw Hill, 1940).

[33] Vinn O, Mutvei H (2009). Calcareous tubeworms of the Phanerozoic. Estonian J. Earth Sci..

[34] Liu Y (2014). *Quadrapyrgites* from the lower Cambrian of South China: growth pattern, post-embryonic development, and affinity. Chin. Sci. Bull..

[35] Jerre F (1994). Anatomy and phylogenetic significance of *Eoconularia loculata*, a conulariid from the Silurian of Gotland. Lethaia.

[36] Simonetta, A. M. & Conway Morris, S. *Early Evolution of Metazoa and the Significance of Problematic Taxa* (Cambridge Univ. Press, 1991).

[37] Hughes NC, Gunderson GO, Weedon MJ (2000). Late Cambrian conulariids from Wisconsin and Minnesota. J. Paleontol..

[38] Van Iten H (2014). Origin and early diversification of the phylum Cnidaria Verrill: major developments in the analysis of the taxon’s Proterozoic-Cambrian history. Palaeontology.

[39] Chapman D, Werner B (1972). Structure of a solitary and a colonial species of *Stephanoscyphus* (Scyphozoa, Coronatae) with observations on periderm repair. Helgol. Meeresunters..

[40] Purcell, J. E. & Angel, D. L. *Jellyfish Blooms: New Problems and Solutions* Vol. 212 (Springer, 2015).

[41] Rees, J. T. A pandeid hydrozoan, *Amphinema* sp., new and probably introduced to central California: life history, morphology, distribution, and systematics. *Sci. Mar.* **64**, 165–172 (2000).

[42] Mendoza-Becerril MA, Marian JEA, Migotto AE, Marques AC (2017). Exoskeletons of Bougainvilliidae and other Hydroidolina (Cnidaria, Hydrozoa): structure and composition. PeerJ.

[43] Mendoza-Becerril MA (2016). An evolutionary comparative analysis of the medusozoan (Cnidaria) exoskeleton. Zool. J. Linn. Soc..

[44] Bayer, F. M., Boschma, H. & Harrington, H. J. *Treatise on Invertebrate Paleontology, Part F: Coelenterata* (Geological Society of America, 1956).

[45] Daly M, Rack F, Zook R (2013). *Edwardsiella andrillae*, a new species of sea anemone from Antarctic Ice. PLoS ONE.

[46] Benayahu Y, McFadden CS, Shoham E (2017). Search for mesophotic octocorals (Cnidaria, Anthozoa) and their phylogeny: I. A new sclerite-free genus from Eilat, northern Red Sea. ZooKeys.

[47] Manuel RL (1977). A redescription of *Edwardsia beautempsi* and *E. timida* (Actiniaria: Edwardsidae). Cah. Biol. Mar..

[48] López-González P, Ocaña O, García-Gómez J, Núñez J (1995). North-eastern Atlantic and Mediterranean species of Cornulariidae Dana, 1846 (Anthozoa: Stolonifera) with the description of a new genus. Zool. Meded..

[49] Kayal E (2018). Phylogenomics provides a robust topology of the major cnidarian lineages and insights on the origins of key organismal traits. BMC Evol. Biol..

[50] Benton MJ (2015). Constraints on the timescale of animal evolutionary history. Palaeontol. Electron..

[51] Omori M, Vervoort W (1986). Observations on a living specimen of the giant hydroid *Branchiocerianthus imperator*. Zool. Meded..

[52] McMahon S, Tarhan LG, Briggs DEG (2017). Decay of the sea anemone *Metridium* (Actinaria): implications for the preservation of cnidarian polyps and other soft-bodied diploblast-grade animals. Palaios.

[53] Brocks JJ (2017). The rise of algae in Cryogenian oceans and the emergence of animals. Nature.

[54] Cohen PA, Riedman LA (2018). It’s a protist-eat-protist world: recalcitrance, predation, and evolution in the Tonian–Cryogenian ocean. Emerg. Top. Life Sci..

[55] Darroch SA, Laflamme M, Clapham ME (2013). Population structure of the oldest known macroscopic communities from Mistaken Point, Newfoundland. Paleobiology.

[56] Kenchington CG, Harris SJ, Vixseboxse PB, Pickup C, Wilby PR (2018). The Ediacaran fossils of Charnwood Forest: shining new light on a major biological revolution. Proc. Geol. Assoc..

[57] Ou Q (2017). Three Cambrian fossils assembled into an extinct body plan of cnidarian affinity. Proc. Natl Acad. Sci. USA.

[58] Lloyd GT (2016). Estimating morphological diversity and tempo with discrete character–taxon matrices: implementation, challenges, progress, and future directions. Biol. J. Linn. Soc..

[59] Whelan NV (2017). Ctenophore relationships and their placement as the sister group to all other animals. Nat. Ecol. Evol..

[60] Gower, J. C. A general coefficient of similarity and some of its properties. *Biometrics* **27**, 857–871 (1971).

[61] Dixon P (2003). VEGAN, a package of R functions for community ecology. J. Veg. Sci..

[62] Clarke KR (1993). Non‐parametric multivariate analyses of changes in community structure. Aust. J. Ecol..

[63] Guillerme T (2018). dispRity: a modular R package for measuring disparity. Methods Ecol. Evol..

[64] Guillerme T, Cooper N (2018). Time for a rethink: time sub‐sampling methods in disparity‐through‐time analyses. Palaeontology.

[65] Vinn O, Kirsimäe K, Parry LA, Toom U (2016). A new Byronia species from the Late Ordovician of Estonia. Estonian J. Earth Sci..

[66] Vinn O, Kirsimäe K (2014). Alleged cnidarian *Sphenothallus* in the Late Ordovician of Baltica, its mineral composition and microstructure. Acta Palaeontol. Pol..

[67] Guo J (2020). A fourteen-faced hexangulaconulariid from the early Cambrian (stage 2) Yanjiahe Formation, South China. J. Paleontol..

[68] Junyuan C, Qingqing P (2005). An Early Cambrian problematic organism Anabarites and its possible affinity. Acta Palaeontol. Sinica.

[69] Van Iten H, Muir LA, Botting JP, Zhang Y, Lin J-P (2013). Conulariids and Sphenothallus (Cnidaria, Medusozoa) from the Tonggao Formation (Lower Ordovician, China). Bull. Geosci..

[70] Cortijo I, Mus MM, Jensen S, Palacios T (2010). A new species of *Cloudina* from the terminal Ediacaran of Spain. Precambrian Res..

[71] Germs GJ (1972). New shelly fossils from Nama Group, south west Africa. Am. J. Sci..

[72] Cai Y, Cortijo I, Schiffbauer JD, Hua H (2017). Taxonomy of the late Ediacaran index fossil *Cloudina* and a new similar taxon from South China. Precambrian Res..

[73] Wills, M. A. in *Fossils, Phylogeny, and Form*: *An Analytical Approach* (eds Adrain, J. M. et al.) 55–144 (Springer, 2001).

[74] Laliberté E, Legendre P (2010). A distance‐based framework for measuring functional diversity from multiple traits. Ecology.

[75] Anderson MJ (2001). A new method for non‐parametric multivariate analysis of variance. Austral Ecol..

[76] Harris, S. *Reflectance Transformation Image of Cast GSM106352 Showing Ediacaran (Pre-Cambrian) Fossils from Charnwood Forest, UK* (NERC EDS National Geoscience Data Centre, 2022); https://webapps.bgs.ac.uk/services/ngdc/accessions/index.html#item173231

