# Supplementary material for: A crown-group cnidarian from the Ediacaran of Charnwood Forest, UK
Source: Nat Ecol Evol. 2022 Jul 25;6(8):1095–104. doi: 10.1038/s41559-022-01807-x (PMC9349040; doi:10.1038/s41559-022-01807-x)
Supplement: Supplementary file 1 — Supplementary discussion including methods and character information on the morphospace and phylogenetic analyses presented in the main text. [file 41559_2022_1807_MOESM1_ESM.pdf]

nature portfolio

https://doi.org/10.1038/s41559-022-01807-x

Supplementary information

A crown-group cnidarian from the Ediacaran of Charnwood Forest, UK

In the format provided by the authors and unedited

A crown-group cnidarian from the Ediacaran of Charnwood Forest, UK.

F. S. Dunn[1*], C.G Kenchington[2], L.A. Parry[3], J.W. Clark[4], R. S. Kendall[5] and P.R. Wilby[6,7]

Included in this document:

Supplementary Discussion| Word file including a history of Ediacaran and Cambrian cnidarians and methods and character information on the morphospace and phylogenetic analyses presented in the main text.

Not Included in this document:

Extended data Figure 1| Unconstrained topologies presented in full. (A) Excluding the fossil taxa *Namacalathus* and *Eolympia*, *Antipathes* and those taxa that posses uninformative character states after safe taxonomic reduction (B) including all taxa. *Auroralumina* is recovered as a cnidarian in both trees. Fossil cnidarians are shown in bold and the position of *Auroralumina* is highlighted with a silhouette. Scale bar for branch lengths is in units of expected number of substitutions per site.

Extended data Figure 2| Constrained topologies. (A) Living cnidarian inter-relationships constrained against recent molecular phylogenies. B) 'Ctenosis' (ctenophores as sister to all other animals) constrained. All fossils were allowed to fully explore treespace under both set of constraints. *Auroralumina* is recovered as a cnidarian in both cases. Fossil cnidarians are shown in bold and the position of *Auroralumina* highted with a silhouette. Scale bar for branch lengths is in units of expected number of substitutions per site.

Extended data Figure 3| Maximum polyp width plotted for extant cnidarian Classes and fossil groups. *Auroralumina* has a much larger polyp width than any other sampled medusozoan. Maximum conulariid polyp width is also larger than any sampled living medusozoan. Source data available with manuscript.

Supplementary data file 1| Nexus file containing morphological phylogenetic dataset (Supplementary data file 1.nex)

Supplementary data file 2| CSV file with Tube morphospace dataset & polyp size data (Supplementary data file 2.csv)

Supplementary data file 3| R script used to analyse tube morphospace (Supplementary data file 3.r)

Supplementary discussion – other Ediacaran potential cnidarians:

Ediacaran frondose fossils - e.g. the rangeomorphs - were historically considered to be related to living sea pens, while isolated holdfast structures (e.g. *Aspidella*, *Cyclomedusa*) were sometimes interpreted as medusae (e.g.[1]). These claims fell out of favour as more information about the growth and anatomy of these fossils came to light[2], but some recent work has suggested once again that rangeomorphs may belong to the total-group Cnidaria on the basis of their functional anatomy[3]. However, cnidarian - or even crown-group eumetazoan - characters (e.g. muscles, digestive cavities) have not been identified in rangeomorphs, with further developmental data from rangeomorphs suggesting they were not able to gastrulate as per crown-group eumetazoans[4], and so are unlikely to represent cnidarian fossils.

Trace fossils from ~565-560 million year old strata have been interpreted to be of actinian/cnidarian grade[5,6] and remain credible evidence for cnidarian-grade organisms. While recent work showing that abiogenic processes may generate similar surface traces casts doubt on the veracity of some of these claims[7], the vertical traces remain uncontested and so we consider them reasonable evidence for cnidarian-grade organisms at this time. Like the majority of trace fossil evidence, however, these fossils cannot be placed confidently in a phylogeny or be used to inform character-state evolution. A recent paper suggested a polypoid ancestor for ctenophores[8], increasing the number of possible trace makers for ichnofossils whose morphology is consistent with a polypoid tracemaker.

Lantianella[9] is described from the Ediacaran-aged Member II of the Lantian Formation, China, in strata older than the Shuram Excursion[9], and consists of a conical body with longitudinal filaments/ribs, with or without transverse banding and showing a crown of unbranched 'tentacle'-like structures. Similarity between *Lantianella* and another fossil, *Flabellophyton*, which has previously been interpreted as an alga[10] on the basis of longitudinal filaments, has led some authors to suggest that *Flabellophyton* may represent a taphomorph of *Lantianella,* which does not preserve tantaculate features. An alternative explanation of it representing an earlier ontogenetic stage of *Lantianella*, perhaps with retracted tentacles[9,11], appears unlikely because the margins of the tentacles are continuous with the cone and do not emanate from within the cone as one might expect for feeding tentacles. Wan and colleagues[9] describe phylogenetically diverse algal taxa which may possess a crown of blades, providing another explanation for such an anatomy, though they were not able to exclude a cnidarian affinity. Van Iten *et al.*[12] interpreted one specimen of *Lantianella* as a possible

conulariid on the basis of what was interpreted as a steeply pyramidal periderm with sub-triangular lappets. However, these structures haven't been observed in other specimens of *Lantianella*[9], which is variable in overall form. Given this uncertainty and lack of diagnostic characters, we do not include *Lantianella* in our analyses nor do we consider it a likely candidate for an Ediacaran cnidarian body fossil.

Two additional Ediacaran-age fossils have drawn comparison to conulariids: *Vendoconularia*[13] and *Paraconularia*[14]. *Vendoconularia* has recently been proposed to belong to the Family Protechiuridae[15], including two other late Ediacaran genera: *Protechiurus* and *Vendoglossus*, with supposed affinities to the coronate scyphozoans via the conulariids and the anabaritiids. However, *Protechiurus* tapers at both ends, which would be incompatible with a cnidarian affinity, and casts doubt on the affinity of *Vendoconularia. Paraconularia* from the Tamengo Formation is described as a conulariid, with a tetraradial, elaborately ornamented tapering periderm, but it is only known - as with *Corumbella* - from close to the Ediacaran-Cambrian boundary and is therefore substantially younger (~15Ma) than *Auroralumina.*

Supplementary discussion - Morphospace and phylogenetic methods and information:

Our morphospace analysis includes 54 taxa 52 characters. Some characters are taken from either the datamatrix of Selly *et al.* 2020[16] or Park *et al.* 2021[17], though note that we split multistate characters into multiple discrete characters. Characters are described below and primary references from which morphological data was acquired are listed in a table. Unless otherwise specified, type species were used.

**Taxon name**	**Key references**
Cloudina carinata	Cortijo *et al.* 2010[18]<br>Park *et al.* 2021[17]
Cloudina hartmanae	Germs 1972[19]<br>Park *et al.* 2021[17]
Cloudina riemkaea	Germs 1972[19]<br>Park *et al.* 2021[17]
Cloudina lucianoi	Adorno *et al.* 2017[20]<br>Park *et al.* 2021[17]

Conotubus	Selly *et al.* 2020[16]<br>Cai *et al.* 2011[21]
Costatubus	Selly *et al.* 2020[16]
Feiyanella	Selly *et al.* 2020[16]<br>Han *et al.* 2017[22]
Multiconotubus	Cai *et al.* 2017[23]<br>Min *et al.* 2019[24]<br>Selly *et al.* 2020[16]
Rajatubulus	Yang *et al.* 2016[25]<br>Selly *et al.* 2020[16]
Saarina hagadorni	Selly *et al.* 2020[16]
Annulatubus	Carbone *et al.* 2015[26]<br>Selly *et al.* 2020[16]
Corumbella	Walde *et al* 2019[27]
Funisia	Droser *et al.* 2008[28]<br>Surprenant *et al.* 2020[29]
Gaojiashania	Selly *et al.* 2020[16]<br>Cai *et al.* 2013[30]
Rugatotheca	Selly *et al.* 2020[16]<br>Yang *et al.* 2016[25]
Sabellidites	Selly *et al.* 2020[16]<br>Ebbestad *et al.* 2021[31]
Sekwitubus	Carbone *et al.* 2015[26]<br>Selly *et al.* 2020[16]
Shaanxilithes	Selly *et al.* 2020[16]<br>Wang *et al.* 2021[32]
Sinotubulites baimatuoensis	Cai *et al.* 2015[33]
Sinotubulites triangularis	Cai *et al.* 2015[33]
Sinotubulites pentacarinalis	Cai *et al.* 2015[33]
Sinotubulites hexagonus	Cai *et al.* 2015[33]
Somatohelix	Sappenfield *et al.* 2011[34]<br>Selly *et al.* 2020[16]
Vendoconularia	Ivantsov and Fedonkin 2002[13]

Wutubus	Chen *et al. 2014*[35]<br>Selly *et al.* 2020[16]
Olivooides	Dong *et al.* 2013[36]
Arthrochites	Conway Morris and Chen 1992[37]
Septuconularia	Guo *et al.* 2020[38]
Pseudooides	Duan *et al.* 2017[39]
Quadrapyrgites	Liu *et al.* 2014[40]
Conulariella	Leme *et al.* 2008[41]<br>Van Iten *et al.* 2013[42]
Paraconularia (Tamengo Formation)	Van Iten *et al.* 2014[43]
Baccaconularia	Hughes *et al.* 2000[44]
Emeiconularia	Yunhuan *et al.* 2005[45]<br>Han *et al.* 2018[46]
Carinachites	Conway Morris and Chen 1992[37]<br>Han *et al.* 2018[46]
Pentaconularia	Liu *et al.* 2011[47]<br>Han *et al.* 2018[46]
Sphenothallus taijiangensis	Dzik *et al.* 2016[48]<br>Zhu *et al.* 2000[49]
Byronia	Zhu *et al.* 2000[49]
Anabarites tripartitus	Junyuan *et al.* 2005[50]<br>Shao *et al.* 2015[51]
Anabarites sexalox	Junyuan *et al.* 2005[50]<br>Shao *et al.* 2015[51]
Hyolithellus	Skovsted and Peel 2011[52]
Cambrocotonus koori	Peel 2017[53]<br>Park *et al.* 2021[17]
Cambroctoconus krygyzstanicus	Geyer *et al.* 2014[54]<br>Park *et al.* 2021[17]
Cambroctoconus coreaensis	Park *et al.* 2016[55]<br>Park *et al.* 2021[17]
Cambroctoconus orientalis	Park *et al.* 2011[56]<br>Park *et al.* 2021[17]

Lipopora lissa	Park *et al.* 2021[17]
Lipopora daseia	Park *et al.* 2021[17]
Tretocylichne	Park *et al.* 2021[17]
Dasyconus	Korde 1964[57]<br>Zhuravlev, Debrenne and Lefuste 1993[58]
Hydroconus	Korde 1964[57]<br>Zhuravlev, Debrenne and Lefuste 1993[58]
Cothonion	Jell and Jell 1976[59]<br>Peel 2011[60]
Moorowipora	Fuller and Jenkins 2007[61]<br>Scrutton 1997[62]
Flindersipora	Fuller and Jenkins 2007[61]<br>Scrutton 1997[62]

Character list

1. Biomineral. Whether a tube is biomineralised or not. *Cloudina, Feiyanella, Multiconotubus, Rajatubulus, Rugatotheca, Sinotubulites,* the conulariids *Conulariella and Baccaconularia,* all carinachitids, hexangulaconulariids, *Byronia, Sphenothallus Anabarites, Hyolithellus, Cambroctoconus, Lipopora, Tretocylichne,* hydroconozoans, *Cothonion* and tabulconids were all scored as present for biomineral skeletons. *Conotubus, Costatubus, Saarina, Gaojiashania, Sabellidites, Shaanxilithes, Somatohelix, Vendoconularia, Paraconularia* (from the Tamengo Formation) and *Auroralumina* were scored as absent. All other taxa were unknown.

    0 absent

    1 present

2. Mineral type. Of the taxa scored present for biomineralisation, the type of mineral was not known in *Multiconotubus, Feiyanella* and *Rajatubulus.* Data on mineral type was harvested from key references and from[63].

    0 phosphate

    1 calcite

    2 aragonite

3. Adapical end cylindrical. The adapical end of all species of *Cloudina* apart from *C. carinata* was scored as cylindrical, *Conotubus, Costatubus, Feiyanella, Multiconotubus, Rajatubulus, Saarina, Annulatubus, Corumbella, Funisia, Gaojianshani, Rugantotheca, Sabellidites, Sekwitubulus, Sinotubulites baimatuoensis, Wutubus, Byronia, Hyolithellus, Dasyconus, Hydroconus, Cothonion, Moorowipora* and *Flindersipora* were all scored as having cylindrical adapical ends. All other taxa were scored as absent.

    0 absent

    1 present

4. Adapical end polyhedral. The adapical end of *Cloudina carinata* is polyhedral, as is the adapical end of olivooids, carinachitids, hexangulaconulariids, conulariids, anabaritids, *Auroralumina* and the tabuloconids. All other taxa were scored as absent.

    0 absent

    1 present

5. Adaptical end flattened. *Shaanxilithes* and *Sphenothallus* are the only taxa scored as present for this character, all others are absent. *Shaanxilithes* was scored from Selly *et al.* 2020 where they term the morphology ‘ribbon-like’.

    0 absent

    1 present

6. Biradial tube. *Shaanxilithes, Arthrochites, Septuconularia, Pseudooides, Sphenothallus* and *Conulariella* were scored as being biradial. *Auroralumina* was scored as ?.

    0 absent

    1 present

7. Tetraradial tube. *Quadrapyrgites, Paraconularia* (from the Tamengo Formation), *Baccaconularia. Auroralumina* was scored as ‘?’ and all other taxa were scored as absent.

    0 absent

    1 present

8. Triradial tube. *Sinotubulites triangularis, Vendoconularia, Emeiconularia* and *Anabarites tripartitus* are both scored as having triradial symmetry, but all other taxa are scored as absent.

0 absent

1 present

9. Pentaradial tube. *Sinotubulites pentacarinalis, Olivooides* and *Pentaconularia* are scored as haing pentaradial symmetry. All other taxa are scored as absent.

    0 absent

    1 present

10. Hexaradial tube. *Sinotubulites hexagonus* and *Anabarites sexalox* are both heaxradial.

    0 absent

    1 present

11. Octoradial tube. *Cloudina carinata* is scored as octoradial, all species of *Cambroctoconus, Lipopora* and *Tretocylichne* were also scored as having octoradial symmetry, following[17]. *Cloudina lucianoi* is '?'.

    0 absent

    1 present

12. Tube tapering. Does the tube decrease in width along its length. All species of *Cloudina, Rajatubulus, Annulatubus, Corumbella, Sekwitubus, Vendoconularia, Wutubus*, olivooids, hexangulaconulariids, conulariids, anabaritiids, *Byronia, Sphenothallus, Hyolithellus,* hydroconozoans and *Cothonion* are all scored as showing a tapering tube. All other taxa are scored as absent.

    0 absent

    1 present

13. Tube taper steep. Some tubes have a noticebly steeper incline and were scored as present for a steep taper. These include the hexangulaconulariids, conulariids and *Cothonion.* All other taxa that were scored as possessing a tapering tube were absent for this character.

    0 absent

    1 present

14. Funnel in funnel structure. Describing the nested tubular anatomy of a number of Ediacaran-Cambrian tubes. All cloudinomorphs (*Cloudina*, *Conotubus*, *Costatubus*, *Feiyanella,*

Multiconotubus, Rajatubulus and *Saarina*) were scored as present for this character, as were *Sinotubulites, Cambroctoconus orientalis and Lipopora. Dasyconus* and *Hydroconus* are scored as '?' because it is not possible to assess whether they show a funnel-in-funnel structure from the published figures. All other taxa were considered absent and were therefore scored as innaplicable for the following four characters.

0 absent

1 present

15. Funnels are true funnels. *Cloudina, Conotubus, Rajatubulus* and *Saarina* have funnels. Funnels are shorter than cones, following[16]

0 absent

1 present

16. Funnels are barrels. *Costatubus, Sinotubulites* and *Lipopora* have barrels.

0 absent

1 present

17. Funnels are cones. *Feiyanella, Multiconotubus* and *Cambroctoconus orientalis* have cones. Cones are longer than funnels, following[16].

0 absent

1 present

18. Degree of overlapping funnel-in-funnel structure. *Cloudina hartmanae* and *Cloudina lucianoi* both overlap more than half an element, as do *Feiyanella* and *Multiconotubus.* All other funnel-in-funnel taxa scored as absent. This character was adapted from Park *et al.* 2021[17].

0 below half of element

1 over half of element

19. Transverse ridges across majority of exterior. *Annulatubus, Corumbella, Funisia, Gaojiashania, Rugatotheca, Sabellidites, Sekwitubulus, Shaanxilithes, Sinotubulites, Somatohelix, Vendoconularia, Wutubus,* olivooids, hexangulaconulariids, conulariids, carinachitids, *Byronia*, *Sphenothallus*, *Hyolithellus*, *Lipopora* are scored as present. Hydroconozoans are scored as ‘?’ because it is not clear whether they exhibit true ridges or

the ridges are an expression of funnel-in-funnel architecture. The following two characters are contingent on the presence of this character.

0 absent

1 present

20. Ridges make up entire external tube morphology or not. Ridges may either account for the entire tube (e.g. *Wutubus*) or may be present overtop a tube structure (e.g. conulariids). Bulbous ridges are found in *Annulatubus, Funisia, Sekwitubulus, Somatohelix, Wutubus, Olivooides* and *Quadrapyrgites.* All other taxa show non-bulbous ridges. If ridges make up entire tube then character 21 is innaplicable.

0 entire tube

1 overlain

21. Continuation of transverse ornament across entire circumference. Some taxa show breaks in the transverse ornament and this may be either assocaited with a longitudinal structure (e.g. sulcus) or not. Taxa scored as showing continuation of the transverse ornament are: *Corumbella, Gaojiashania, Rugatotheca, Sabellidites, Shaanxilithes, Sinotubulites, Pseudooides, Paraconularia* (based on the genus), *Byronia, Sphenothallus, Hyolithellus, Lipopora.* All other taxa are scored as absent apart from *Baccaconularia* where the available images are not sufficient to be able to say with certainty, so we leave it as '?'.

0 absent

1 present

22. Tubercles on exterior. Tubercles may be any shape and associated (or not) with any structure. *Pseudooides, Quadrapyrgites, Paraconularia* and *Baccaconularia* are scored as present. All other taxa are considered absent and so are scored as innaplicable for the following two characters.

0 absent

1 present

23. Tubercles associated with transverse ribs. Only *Paraconularia* and *Baccaconularia* are scored present for this character.

0 absent

1 present

24. Tubercles are thorn-like projections. Only *Quadrapyrgites* displays tubercles as thorny projections.

    0 absent

    1 present

25. Longitudinal ornament. This character does not include sulci. *Olivooides, Arthrochites, Quadrapyrgites,* carinachitids and *Byronia* are scored as present. All other taxa are absent for this character.

    0 absent

    1 present

26. Closed terminal apex. For taxa with an incomplete apex - *Auroralumina* and the carinachitids – this was left as '?', and so too for taxa where it is not possible to assess the apex of individual tubes from published figures (*Morrowipora* and *Flindersipora*). *Cloudina carinata, Cloudina riemkaea, Cloudina lucianoi, Conotubus, Feiyanella, Multiconotubus, Saarina, Corumbella, Funisia, Sekwitubulus, Somatohelix, Vendoconularia, Wutubus,* olivooids, hexangulaconulariids, conulariids, *Sphenothallus, Byronia, Anabarites, Cambroctoconus, Lipopora, Tetrocylichne, Dasyconus* and *Cothonion* were scored as present for this character and all others were scored absent. The following two characters are contingent on the presence of a closed apex.

    0 absent

    1 present

27. Apical boss with different ornament. The difference in ornament may be either in the presence of dimples or the presence of tighter transverse ornament. Olivoiods, hexangulaconulariids and *Conulariella* are scored as present.

    0 absent

    1 present

28. Holdfast (attachment disc). *Cloudina hartmanae, Funisia, Sekwitubulus, Sphenothallus, Cambroctoconus, Lipopora, Tetroclichine, Dasyconus* and *Cothonion* are scored as present, while the remaining three species of *Cloudina, Costatubus, Rajatubulus, Saarina,* all

conulariids, carinachitids and *Auroralumina* are scored as unknown. All other taxa are scored as absent.

0 absent

1 present

29. Oral lobes at aperture. This is scored as presnet in *Olivooides, Quadrapyrgites* and the carinachitids. It is absent in all other taxa.

0 absent

1 present

30. Operculum. This is present in *Cothonion* and absent in all other taxa.

0 absent

1 present

31. Tube separated into distinct zones. This may be at any height along the tube and is demonstrated by rapid transitions in the width of the tube. It is present in *Auroralumina* and the hydroconozoans, but absent in all other taxa.

0 absent

1 present

32. Tubes have faces. Distinct faces are present in *Cloudina carinata, Sinotubulites triangularis, Sinotubulites pentacarinalis, Sinotubulites hexagonus, Vendoconularia,* olivooids, carinachitids, hexangulaconulariids, *conulariids, Sphenothallus, Auroralumina, Anabarites, Cambroctoconus, Lipopora* and *Tretocylichne,* but are absent in all other taxa.

0 absent

1 present

33. Tubes have external midline(s). Midline(s) are present in the hexangulaconulariids and *Paraconularia*, but are absent in all other taxa.

0 absent

1 present

34. Midlines between tubes. This is contingent on a colonial lifestyle. It is scored as present in *Moorowipora* and *Flindersipora*, absent in *Cloudina carinata, Cloudina hartmanae, Cloudina riemkaea*, *Feiyanella*, *Funisia*, *Sphenothallus*, *Auroralumina*, *Cambroctoconus*,

Lipopora lissa, Tretocylichne are all scored as absent as they either are clearly colonial or exhibit branching. All other taxa are scored as innaplicable.

0 absent

1 present

35. Tubes have sulci. As sulci impart a polyhedral symmetry on the tube, taxa which lack polyhedral symmetry - *Sinotubulites baimatuoensis* and the hydroconozoans – are scored as innaplicable. *Conulariella, Paraconularia,* the carinachitids, *Auroralumina, Anabarites* and *Cothonion* are scored as present and all others absent.

0 absent

1 present

36. Tubes are aproximately straight. Many tubes exhibit some curvature (e.g. *Anabarites*), but some are markedly bent or twisted (e.g. *Somatohelix*). This is the morphology we intend to pick out with this character. *Cloudina hartmanae, Sinotubulites, Vendoconularia, Wutubus,* olovooids, hxangulaconulariids, conulariids, carinachitids, *Auroralumina, Byronia, Sphenothallus, Hyolithellus, Lipopora,* hydroconozoans, *Cothonion, Moorowipora* and Flindersipora are all scored as showing straight tubes and all others as absent for this character.

0 absent

1 present

37. Branching observed. Branching has been observed in: *Cloudina, Feiyanella, Funisia, Sphenothallus, Auroralumina, Cambroctoconus krygyzstanics, Cambroctoconus coreaensis, Cambroctoconus orientalis, Lipopora lissa, Tretocylichne, Morrowipora* and *Flindersipora* but is absent in all other taxa.

0 absent

1 present

38. Ceroid growth. A type of colony formation with many tubes, each retaining their own tube wall. Tubes are packed together and so branching as observed in *Cloudina* or *Auroralumina* does not represent this growth form. This is scored as present in *Morrowipora* and *Flindersipora* only.

0 absent

1 present

39. Parricidal increase. Describing a form of colony growth which can only be scored in taxa which show branching. It is scored as present in *Moorowipora* and *Flindersipora* and absent in all other taxa.

    0 absent

    1 present

40. Di/Trichotomous branching. This is scored as present in *Cloudina carinata, Cloudina hartmanae, Cloudina riemkaea, Feiyanella, Funisia, Auroralumina, Lipopora lissa, Morrowipora and Flindersipora* and absent in all other branching taxa.

    0 absent

    1 presen

    t

41. Budding from outer surface of skeleton. This is scored as innaplicable in all taxa in which branching is not observed. *Sphenothallus, Cambroctoconus kyrgyzstanicus, Cambroctoconus coreaensis, Cambroctoconus orientalis, Lipopora lissa* and *Tretocylichne* are all scored as present and other taxa are scored as absent. *Cambroctoconus koori* is left '?', following Park *et al.* 2021[17].

    0 absent

    1 present

42. Budding from inner surface of skeleton. This is not contingent on an outwardly branching anatomy because it may not result in an outwardly branched morphology. This is scored as present in *Cloudina lucianoi, Cambroctoconus kyrgyzstanicus, Cambroctoconus coreaensi* and *Cambroctoconus orientalis* and absent in other taxa. It is scored as unknown in txa where the preservational style mean the internal anatomy is not known: *Annulatubus, Funisia, Sekwitubulus, Somatohellix, Vendoconularia, Gaojiashania* and *Wutubus.* The hydroconozoans and *Auroralumina* are also scored as '?' because small specimen size means that the available material is not a reliable test and *Cambroctoconus koori* is left '?', following Park *et al.* 2021[17].

    0 absent

    1 present

43. Internal septa. We use the term septa in the broadest sense, to mean invaginations from the tube which do not reach the other tube wall, but separate this from sulci which are folds in the wall. Olivooids, *Hyolithellus, Cambroctoconus, Lipopora, Tretocylichne,* hydroconozoans, *Cothonion, Flindersipora* and *Moorowipora* are scored as present, and all other taxa are absent except for *Auroralumina, Sphenothallus, Baccaconularia, Paraconularia, Conulariella,* hexangulaconulariids, *Wutubus, Sekwitubus, Gaojiashania, Funisia* and *Annulatubus,* which are scored as unknown because we do not have access to their internal anatomy. The following two characters are contingent on the presence of septa.

    0 absent

    1 present

44. Number of septa is 8 or multiple of 8. This is present in *Cambroctoconus koori, Cambroctoconus krygzstanicus, Cambroctoconus orientalis, Lipopora lissa* and *Tretocylichne* and absent in all other septate tubes. There are no consitent septal patterns in any other group of sampled tubes so we refrain from errecting further characters.

    0 absent

    1 present

45. Septa paired. This is presence in olivooids, *Cambroctoconus krygzstanicus, Cambroctoconus orientalis* and *Tretocylichne,* but absent in all other septate tubes.

    0 absent

    1 present

46. Horizontal internal elements. This refers to features like tabulae and is scored as present in *Cambroctoconus krygzstanicus, Cambroctoconus orientalis, Moorowiopora* and *Flindersipora.* It is scored as unknown – as per internal septa – in *Auroralumina, Sphenothallus, Baccaconularia, Paraconularia, Conulariella,* hexangulaconulariids, *Wutubus, Sekwitubus, Gaojiashania, Funisia* and *Annulatubus.* All other taxa are scored as absent.

    0 absent

    1 present

47. Smooth inner walls. This is scored as present in *Cloudina carinata, Cloudina riemkaea, Cloudina lucianoi, Conotubus, Costatubus, Multiconotubus, Saarina, Sabellidites,*

Shaanxilithes, Sinotubulites and *Anabarites,* carinachitids and unknown in the same taxa as per internal septa: *Auroralumina, Sphenothallus, Baccaconularia, Paraconularia, Conulariella,* hexangulaconulariids, *Wutubus, Sekwitubus, Gaojiashania, Funisia* and *Annulatubus.* All other taxa are scored as absent.

0 absent

1 present

48. Tubes multi-layered. This does not include overlapping funnel-in-funnel tubes. This is scored as present in *Feiyanella, Multiconotubus, Shaanxilithes, Sinotubulites* and *Dasyconus* and unknown in taxa preserved by cast and mould (*Funisia, Somatohelix* and *Auroralumina*), but absent in all other taxa.

0 absent

1 present

49. Perforations in the skeletal wall. This is scored as present in *Cambroctoconus, Lipopora, Tretocylichne, Hydroconus, Moorowipora* and *Flindersipora.* It is scored as unknown in *Dasyconus,* where only one specimen is known through thin section and absent in all other taxa. The next character is contingent on the presence of this character.

0 absent

1 present

50. Longitudinal canals opening to exterior. This is present in *Hydroconus* only and unknown in *Dasyconus.*

0 absent

1 present

Phylogenetic methods:

We conducted our analyses using Bayesian Inference, implemented in MrBayes 3.2.6, since probabilistic approaches are considered to provide more accurate results when analysing discrete morphological data[64,65]. We followed the multistate coding strategy of Brazeau (2011)[66]. Our matrix was based on Zhao *et al.* (2019[8]), which focuses on cnidarians and ctenophores. We have extended the taxon sample to include the extant cubozoan *Morbakka*[67] and the fossil taxa *Eoconularia*[68], *Conularia*[69], *Carinachites*[46] and *Auroralumina.* Although retained in our matrix, the taxa *Agalma, Keratoisidinae, Thaumactena, Trigooides* and *Craseoa* were removed as they did not possess

informative character combinations, and *Antipathes* was removed because it collapsed the Anthozoan crown-group node. The fossil taxa *Eolympia* and *Namacalathus* were not included in the topology presented in main text Fig. 4 because they have been omitted from previous studies because of very large amounts of missing data and equivocal taxonomic status[8].

Our presented topology is run without any topological constraints. However, as the inter-relationships of some of our recovered clades conflicts with recent molecular phylogenies[70,71], we conducted additional analyses where we constrained our tree topology to that of molecular studies. Additionally, to ensure our recovered topology was not the direct result of our recovery of a sister relationship between the Cnidaria and the Ctenophora, we conduced an analysis constraining ctenophores as the sister to all other animals (Ctenosis), as is recovered by some molecular phylogenies[72]. Topological constraints are presented below.

Topological constraints:

Partial constraints were used to allow all fossil taxa to wander.

Cnidarian inter-relationships following recent molecular phylogenies:

constraint node1 partial = Nematostella Anemonia Aiptasia Metridium Antipathes Ceriantharia Corynactis Montastraea Porites Acropora Parazoanthus Anthomastus Leptogorgia Virgularia Haliclystus Lucernaria Calvadosia Alatina Chironex Morbakka Atolla Stephanoscyphus Nausithoe Aurelia Cassiopea Rhizostoma Hydra Candelabrum Hydractinia Ectopleura Clytia Obelia Physalia Craseoa Abylopsis Agalma Nanomia Aeginia Halitrephes : Choanoflagellata Demospongae Homoscleromorpha Calcarea Hexactinellida Placozoa Euplokamis Mnemiopsis Beroida Platyctenida Cestida Ganeshida Thalassocalycida Bryozoa Brachiopoda Phoronida Mollusca Annelida Echinodermata Pterobranchia Chaetognatha Onychophora Arthropoda Nemertea Enteropneusta Urochordata Cephalochordata Vertebrata Entoprocta Nematoda Nematomorpha Priapulida Gastrotricha Tardigrada Kinorhyncha Loricifera Gnathifera Xenacoelomorpha Platyhelminthes ;

constraint node2 partial = Nematostella Anemonia Aiptasia Metridium Antipathes Ceriantharia Corynactis Montastraea Porites Acropora Parazoanthus Anthomastus Leptogorgia Virgularia : Haliclystus Lucernaria Calvadosia Alatina Chironex Morbakka Atolla Stephanoscyphus Nausithoe Aurelia Cassiopea Rhizostoma Hydra Candelabrum Hydractinia Ectopleura Clytia Obelia Physalia Craseoa Abylopsis Agalma Nanomia Aeginia Halitrephes Choanoflagellata Demospongae

Homoscleromorpha Calcarea Hexactinellida Placozoa Euplokamis Mnemiopsis Beroida Platyctenida Cestida Ganeshida Thalassocalycida Bryozoa Brachiopoda Phoronida Mollusca Annelida Echinodermata Pterobranchia Chaetognatha Onychophora Arthropoda Nemertea Enteropneusta Urochordata Cephalochordata Vertebrata Entoprocta Nematoda Nematomorpha Priapulida Gastrotricha Tardigrada Kinorhyncha Loricifera Gnathifera Xenacoelomorpha Platyhelminthes ;

constraint node3 partial = Anthomastus Leptogorgia Virgularia : Nematostella Anemonia Aiptasia Metridium Antipathes Ceriantharia Corynactis Montastraea Porites Acropora Parazoanthus Haliclystus Lucernaria Calvadosia Alatina Chironex Morbakka Atolla Stephanoscyphus Nausithoe Aurelia Cassiopea Rhizostoma Hydra Candelabrum Hydractinia Ectopleura Clytia Obelia Physalia Craseoa Abylopsis Agalma Nanomia Aeginia Halitrephes Choanoflagellata Demospongae Homoscleromorpha Calcarea Hexactinellida Placozoa Euplokamis Mnemiopsis Beroida Platyctenida Cestida Ganeshida Thalassocalycida Bryozoa Brachiopoda Phoronida Mollusca Annelida Echinodermata Pterobranchia Chaetognatha Onychophora Arthropoda Nemertea Enteropneusta Urochordata Cephalochordata Vertebrata Entoprocta Nematoda Nematomorpha Priapulida Gastrotricha Tardigrada Kinorhyncha Loricifera Gnathifera Xenacoelomorpha Platyhelminthes ;

constraint node4 partial = Nematostella Anemonia Aiptasia Metridium Antipathes Ceriantharia Corynactis Montastraea Porites Acropora Parazoanthus : Anthomastus Leptogorgia Virgularia Haliclystus Lucernaria Calvadosia Alatina Chironex Morbakka Atolla Stephanoscyphus Nausithoe Aurelia Cassiopea Rhizostoma Hydra Candelabrum Hydractinia Ectopleura Clytia Obelia Physalia Craseoa Abylopsis Agalma Nanomia Aeginia Halitrephes Choanoflagellata Demospongae Homoscleromorpha Calcarea Hexactinellida Placozoa Euplokamis Mnemiopsis Beroida Platyctenida Cestida Ganeshida Thalassocalycida Bryozoa Brachiopoda Phoronida Mollusca Annelida Echinodermata Pterobranchia Chaetognatha Onychophora Arthropoda Nemertea Enteropneusta Urochordata Cephalochordata Vertebrata Entoprocta Nematoda Nematomorpha Priapulida Gastrotricha Tardigrada Kinorhyncha Loricifera Gnathifera Xenacoelomorpha Platyhelminthes ;

constraint node5 partial = Nematostella Anemonia Aiptasia Metridium Parazoanthus : Antipathes Ceriantharia Corynactis Montastraea Porites Acropora Anthomastus Leptogorgia Virgularia Haliclystus Lucernaria Calvadosia Alatina Chironex Morbakka Atolla Stephanoscyphus Nausithoe Aurelia Cassiopea Rhizostoma Hydra Candelabrum Hydractinia Ectopleura Clytia Obelia Physalia Craseoa Abylopsis Agalma Nanomia Aeginia Halitrephes Choanoflagellata Demospongae Homoscleromorpha Calcarea Hexactinellida Placozoa Euplokamis Mnemiopsis Beroida Platyctenida

Cestida Ganeshida Thalassocalycida Bryozoa Brachiopoda Phoronida Mollusca Annelida Echinodermata Pterobranchia Chaetognatha Onychophora Arthropoda Nemertea Enteropneusta Urochordata Cephalochordata Vertebrata Entoprocta Nematoda Nematomorpha Priapulida Gastrotricha Tardigrada Kinorhyncha Loricifera Gnathifera Xenacoelomorpha Platyhelminthes ;

constraint node6 partial = Nematostella Anemonia Aiptasia Metridium : Parazoanthus Antipathes Ceriantharia Corynactis Montastraea Porites Acropora Anthomastus Leptogorgia Virgularia Haliclystus Lucernaria Calvadosia Alatina Chironex Morbakka Atolla Stephanoscyphus Nausithoe Aurelia Cassiopea Rhizostoma Hydra Candelabrum Hydractinia Ectopleura Clytia Obelia Physalia Craseoa Abylopsis Agalma Nanomia Aeginia Halitrephes Choanoflagellata Demospongae Homoscleromorpha Calcarea Hexactinellida Placozoa Euplokamis Mnemiopsis Beroida Platyctenida Cestida Ganeshida Thalassocalycida Bryozoa Brachiopoda Phoronida Mollusca Annelida Echinodermata Pterobranchia Chaetognatha Onychophora Arthropoda Nemertea Enteropneusta Urochordata Cephalochordata Vertebrata Entoprocta Nematoda Nematomorpha Priapulida Gastrotricha Tardigrada Kinorhyncha Loricifera Gnathifera Xenacoelomorpha Platyhelminthes ;

constraint node7 partial = Anemonia Aiptasia Metridium : Nematostella Parazoanthus Antipathes Ceriantharia Corynactis Montastraea Porites Acropora Anthomastus Leptogorgia Virgularia Haliclystus Lucernaria Calvadosia Alatina Chironex Morbakka Atolla Stephanoscyphus Nausithoe Aurelia Cassiopea Rhizostoma Hydra Candelabrum Hydractinia Ectopleura Clytia Obelia Physalia Craseoa Abylopsis Agalma Nanomia Aeginia Halitrephes Choanoflagellata Demospongae Homoscleromorpha Calcarea Hexactinellida Placozoa Euplokamis Mnemiopsis Beroida Platyctenida Cestida Ganeshida Thalassocalycida Bryozoa Brachiopoda Phoronida Mollusca Annelida Echinodermata Pterobranchia Chaetognatha Onychophora Arthropoda Nemertea Enteropneusta Urochordata Cephalochordata Vertebrata Entoprocta Nematoda Nematomorpha Priapulida Gastrotricha Tardigrada Kinorhyncha Loricifera Gnathifera Xenacoelomorpha Platyhelminthes ;

constraint node8 partial = Aiptasia Metridium : Anemonia Antipathes Nematostella Parazoanthus Ceriantharia Corynactis Montastraea Porites Acropora Anthomastus Leptogorgia Virgularia Haliclystus Lucernaria Calvadosia Alatina Chironex Morbakka Atolla Stephanoscyphus Nausithoe Aurelia Cassiopea Rhizostoma Hydra Candelabrum Hydractinia Ectopleura Clytia Obelia Physalia Craseoa Abylopsis Agalma Nanomia Aeginia Halitrephes Choanoflagellata Demospongae Homoscleromorpha Calcarea Hexactinellida Placozoa Euplokamis Mnemiopsis Beroida Platyctenida Cestida Ganeshida Thalassocalycida Bryozoa Brachiopoda Phoronida Mollusca Annelida

Echinodermata Pterobranchia Chaetognatha Onychophora Arthropoda Nemertea Enteropneusta Urochordata Cephalochordata Vertebrata Entoprocta Nematoda Nematomorpha Priapulida Gastrotricha Tardigrada Kinorhyncha Loricifera Gnathifera Xenacoelomorpha Platyhelminthes ;

constraint node9 partial = Corynactis Montastraea Porites Acropora Antipathes : Nematostella Anemonia Aiptasia Metridium Ceriantharia Parazoanthus Anthomastus Leptogorgia Virgularia Haliclystus Lucernaria Calvadosia Alatina Chironex Morbakka Atolla Stephanoscyphus Nausithoe Aurelia Cassiopea Rhizostoma Hydra Candelabrum Hydractinia Ectopleura Clytia Obelia Physalia Craseoa Abylopsis Agalma Nanomia Aeginia Halitrephes Choanoflagellata Demospongae Homoscleromorpha Calcarea Hexactinellida Placozoa Euplokamis Mnemiopsis Beroida Platyctenida Cestida Ganeshida Thalassocalycida Bryozoa Brachiopoda Phoronida Mollusca Annelida Echinodermata Pterobranchia Chaetognatha Onychophora Arthropoda Nemertea Enteropneusta Urochordata Cephalochordata Vertebrata Entoprocta Nematoda Nematomorpha Priapulida Gastrotricha Tardigrada Kinorhyncha Loricifera Gnathifera Xenacoelomorpha Platyhelminthes ;

constraint node10 partial = Montastraea Porites Acropora : Antipathes Corynactis Nematostella Anemonia Aiptasia Metridium Ceriantharia Parazoanthus Anthomastus Leptogorgia Virgularia Haliclystus Lucernaria Calvadosia Alatina Chironex Morbakka Atolla Stephanoscyphus Nausithoe Aurelia Cassiopea Rhizostoma Hydra Candelabrum Hydractinia Ectopleura Clytia Obelia Physalia Craseoa Abylopsis Agalma Nanomia Aeginia Halitrephes Choanoflagellata Demospongae Homoscleromorpha Calcarea Hexactinellida Placozoa Euplokamis Mnemiopsis Beroida Platyctenida Cestida Ganeshida Thalassocalycida Bryozoa Brachiopoda Phoronida Mollusca Annelida Echinodermata Pterobranchia Chaetognatha Onychophora Arthropoda Nemertea Enteropneusta Urochordata Cephalochordata Vertebrata Entoprocta Nematoda Nematomorpha Priapulida Gastrotricha Tardigrada Kinorhyncha Loricifera Gnathifera Xenacoelomorpha Platyhelminthes ;

constraint node11 partial = Porites Acropora : Nematostella Anemonia Aiptasia Metridium Antipathes Ceriantharia Corynactis Montastraea Parazoanthus Anthomastus Leptogorgia Virgularia Haliclystus Lucernaria Calvadosia Alatina Chironex Morbakka Atolla Stephanoscyphus Nausithoe Aurelia Cassiopea Rhizostoma Hydra Candelabrum Hydractinia Ectopleura Clytia Obelia Physalia Craseoa Abylopsis Agalma Nanomia Aeginia Halitrephes Choanoflagellata Demospongae Homoscleromorpha Calcarea Hexactinellida Placozoa Euplokamis Mnemiopsis Beroida Platyctenida Cestida Ganeshida Thalassocalycida Bryozoa Brachiopoda Phoronida Mollusca Annelida Echinodermata Pterobranchia Chaetognatha Onychophora Arthropoda Nemertea Enteropneusta Urochordata Cephalochordata

Vertebrata Entoprocta Nematoda Nematomorpha Priapulida Gastrotricha Tardigrada Kinorhyncha Loricifera Gnathifera Xenacoelomorpha Platyhelminthes ;

constraint node12 partial = Haliclystus Lucernaria Calvadosia Alatina Chironex Morbakka Atolla Stephanoscyphus Nausithoe Aurelia Cassiopea Rhizostoma Hydra Candelabrum Hydractinia Ectopleura Clytia Obelia Physalia Craseoa Abylopsis Agalma Nanomia Aeginia Halitrephes : Nematostella Anemonia Aiptasia Metridium Antipathes Ceriantharia Corynactis Montastraea Porites Acropora Parazoanthus Anthomastus Leptogorgia Virgularia Choanoflagellata Demospongae Homoscleromorpha Calcarea Hexactinellida Placozoa Euplokamis Mnemiopsis Beroida Platyctenida Cestida Ganeshida Thalassocalycida Bryozoa Brachiopoda Phoronida Mollusca Annelida Echinodermata Pterobranchia Chaetognatha Onychophora Arthropoda Nemertea Enteropneusta Urochordata Cephalochordata Vertebrata Entoprocta Nematoda Nematomorpha Priapulida Gastrotricha Tardigrada Kinorhyncha Loricifera Gnathifera Xenacoelomorpha Platyhelminthes ;

constraint node13 partial = Haliclystus Lucernaria Calvadosia Alatina Chironex Morbakka Atolla Stephanoscyphus Nausithoe Aurelia Cassiopea Rhizostoma : Hydra Candelabrum Hydractinia Ectopleura Clytia Obelia Physalia Craseoa Abylopsis Agalma Nanomia Aeginia Halitrephes Nematostella Anemonia Aiptasia Metridium Antipathes Ceriantharia Corynactis Montastraea Porites Acropora Parazoanthus Anthomastus Leptogorgia Virgularia Choanoflagellata Demospongae Homoscleromorpha Calcarea Hexactinellida Placozoa Euplokamis Mnemiopsis Beroida Platyctenida Cestida Ganeshida Thalassocalycida Bryozoa Brachiopoda Phoronida Mollusca Annelida Echinodermata Pterobranchia Chaetognatha Onychophora Arthropoda Nemertea Enteropneusta Urochordata Cephalochordata Vertebrata Entoprocta Nematoda Nematomorpha Priapulida Gastrotricha Tardigrada Kinorhyncha Loricifera Gnathifera Xenacoelomorpha Platyhelminthes ;

constraint node14 partial = Alatina Chironex Morbakka Atolla Stephanoscyphus Nausithoe Aurelia Cassiopea Rhizostoma : Haliclystus Lucernaria Calvadosia Hydra Candelabrum Hydractinia Ectopleura Clytia Obelia Physalia Craseoa Abylopsis Agalma Nanomia Aeginia Halitrephes Nematostella Anemonia Aiptasia Metridium Antipathes Ceriantharia Corynactis Montastraea Porites Acropora Parazoanthus Anthomastus Leptogorgia Virgularia Choanoflagellata Demospongae Homoscleromorpha Calcarea Hexactinellida Placozoa Euplokamis Mnemiopsis Beroida Platyctenida Cestida Ganeshida Thalassocalycida Bryozoa Brachiopoda Phoronida Mollusca Annelida Echinodermata Pterobranchia Chaetognatha Onychophora Arthropoda Nemertea Enteropneusta

Urochordata Cephalochordata Vertebrata Entoprocta Nematoda Nematomorpha Priapulida Gastrotricha Tardigrada Kinorhyncha Loricifera Gnathifera Xenacoelomorpha Platyhelminthes ;

constraint node15 partial = Haliclystus Lucernaria Calvadosia : Alatina Chironex Morbakka Atolla Stephanoscyphus Nausithoe Aurelia Cassiopea Rhizostoma Hydra Candelabrum Hydractinia Ectopleura Clytia Obelia Physalia Craseoa Abylopsis Agalma Nanomia Aeginia Halitrephes Nematostella Anemonia Aiptasia Metridium Antipathes Ceriantharia Corynactis Montastraea Porites Acropora Parazoanthus Anthomastus Leptogorgia Virgularia Choanoflagellata Demospongae Homoscleromorpha Calcarea Hexactinellida Placozoa Euplokamis Mnemiopsis Beroida Platyctenida Cestida Ganeshida Thalassocalycida Bryozoa Brachiopoda Phoronida Mollusca Annelida Echinodermata Pterobranchia Chaetognatha Onychophora Arthropoda Nemertea Enteropneusta Urochordata Cephalochordata Vertebrata Entoprocta Nematoda Nematomorpha Priapulida Gastrotricha Tardigrada Kinorhyncha Loricifera Gnathifera Xenacoelomorpha Platyhelminthes ;

constraint node16 partial = Lucernaria Calvadosia : Haliclystus Alatina Chironex Morbakka Atolla Stephanoscyphus Nausithoe Aurelia Cassiopea Rhizostoma Hydra Candelabrum Hydractinia Ectopleura Clytia Obelia Physalia Craseoa Abylopsis Agalma Nanomia Aeginia Halitrephes Nematostella Anemonia Aiptasia Metridium Antipathes Ceriantharia Corynactis Montastraea Porites Acropora Parazoanthus Anthomastus Leptogorgia Virgularia Choanoflagellata Demospongae Homoscleromorpha Calcarea Hexactinellida Placozoa Euplokamis Mnemiopsis Beroida Platyctenida Cestida Ganeshida Thalassocalycida Bryozoa Brachiopoda Phoronida Mollusca Annelida Echinodermata Pterobranchia Chaetognatha Onychophora Arthropoda Nemertea Enteropneusta Urochordata Cephalochordata Vertebrata Entoprocta Nematoda Nematomorpha Priapulida Gastrotricha Tardigrada Kinorhyncha Loricifera Gnathifera Xenacoelomorpha Platyhelminthes ;

constraint node17 partial = Alatina Chironex Morbakka : Haliclystus Lucernaria Calvadosia Atolla Stephanoscyphus Nausithoe Aurelia Cassiopea Rhizostoma Hydra Candelabrum Hydractinia Ectopleura Clytia Obelia Physalia Craseoa Abylopsis Agalma Nanomia Aeginia Halitrephes Nematostella Anemonia Aiptasia Metridium Antipathes Ceriantharia Corynactis Montastraea Porites Acropora Parazoanthus Anthomastus Leptogorgia Virgularia Choanoflagellata Demospongae Homoscleromorpha Calcarea Hexactinellida Placozoa Euplokamis Mnemiopsis Beroida Platyctenida Cestida Ganeshida Thalassocalycida Bryozoa Brachiopoda Phoronida Mollusca Annelida Echinodermata Pterobranchia Chaetognatha Onychophora Arthropoda Nemertea Enteropneusta

Urochordata Cephalochordata Vertebrata Entoprocta Nematoda Nematomorpha Priapulida Gastrotricha Tardigrada Kinorhyncha Loricifera Gnathifera Xenacoelomorpha Platyhelminthes ;

constraint node18 partial = Atolla Stephanoscyphus Nausithoe Aurelia Cassiopea Rhizostoma : Haliclystus Lucernaria Calvadosia Alatina Chironex Morbakka Hydra Candelabrum Hydractinia Ectopleura Clytia Obelia Physalia Craseoa Abylopsis Agalma Nanomia Aeginia Halitrephes Nematostella Anemonia Aiptasia Metridium Antipathes Ceriantharia Corynactis Montastraea Porites Acropora Parazoanthus Anthomastus Leptogorgia Virgularia Choanoflagellata Demospongae Homoscleromorpha Calcarea Hexactinellida Placozoa Euplokamis Mnemiopsis Beroida Platyctenida Cestida Ganeshida Thalassocalycida Bryozoa Brachiopoda Phoronida Mollusca Annelida Echinodermata Pterobranchia Chaetognatha Onychophora Arthropoda Nemertea Enteropneusta Urochordata Cephalochordata Vertebrata Entoprocta Nematoda Nematomorpha Priapulida Gastrotricha Tardigrada Kinorhyncha Loricifera Gnathifera Xenacoelomorpha Platyhelminthes ;

constraint node19 partial = Atolla Stephanoscyphus Nausithoe : Aurelia Cassiopea Rhizostoma Haliclystus Lucernaria Calvadosia Alatina Chironex Morbakka Hydra Candelabrum Hydractinia Ectopleura Clytia Obelia Physalia Craseoa Abylopsis Agalma Nanomia Aeginia Halitrephes Nematostella Anemonia Aiptasia Metridium Antipathes Ceriantharia Corynactis Montastraea Porites Acropora Parazoanthus Anthomastus Leptogorgia Virgularia Choanoflagellata Demospongae Homoscleromorpha Calcarea Hexactinellida Placozoa Euplokamis Mnemiopsis Beroida Platyctenida Cestida Ganeshida Thalassocalycida Bryozoa Brachiopoda Phoronida Mollusca Annelida Echinodermata Pterobranchia Chaetognatha Onychophora Arthropoda Nemertea Enteropneusta Urochordata Cephalochordata Vertebrata Entoprocta Nematoda Nematomorpha Priapulida Gastrotricha Tardigrada Kinorhyncha Loricifera Gnathifera Xenacoelomorpha Platyhelminthes ;

constraint node20 partial = Aurelia Cassiopea Rhizostoma : Atolla Stephanoscyphus Nausithoe Haliclystus Lucernaria Calvadosia Alatina Chironex Morbakka Hydra Candelabrum Hydractinia Ectopleura Clytia Obelia Physalia Craseoa Abylopsis Agalma Nanomia Aeginia Halitrephes Nematostella Anemonia Aiptasia Metridium Antipathes Ceriantharia Corynactis Montastraea Porites Acropora Parazoanthus Anthomastus Leptogorgia Virgularia Choanoflagellata Demospongae Homoscleromorpha Calcarea Hexactinellida Placozoa Euplokamis Mnemiopsis Beroida Platyctenida Cestida Ganeshida Thalassocalycida Bryozoa Brachiopoda Phoronida Mollusca Annelida Echinodermata Pterobranchia Chaetognatha Onychophora Arthropoda Nemertea Enteropneusta

Urochordata Cephalochordata Vertebrata Entoprocta Nematoda Nematomorpha Priapulida Gastrotricha Tardigrada Kinorhyncha Loricifera Gnathifera Xenacoelomorpha Platyhelminthes ;

constraint node21 partial = Hydra Candelabrum Hydractinia Ectopleura Clytia Obelia Physalia Craseoa Abylopsis Agalma Nanomia Aeginia Halitrephes : Haliclystus Lucernaria Calvadosia Alatina Chironex Morbakka Atolla Stephanoscyphus Nausithoe Aurelia Cassiopea Rhizostoma Nematostella Anemonia Aiptasia Metridium Antipathes Ceriantharia Corynactis Montastraea Porites Acropora Parazoanthus Anthomastus Leptogorgia Virgularia Choanoflagellata Demospongae Homoscleromorpha Calcarea Hexactinellida Placozoa Euplokamis Mnemiopsis Beroida Platyctenida Cestida Ganeshida Thalassocalycida Bryozoa Brachiopoda Phoronida Mollusca Annelida Echinodermata Pterobranchia Chaetognatha Onychophora Arthropoda Nemertea Enteropneusta Urochordata Cephalochordata Vertebrata Entoprocta Nematoda Nematomorpha Priapulida Gastrotricha Tardigrada Kinorhyncha Loricifera Gnathifera Xenacoelomorpha Platyhelminthes ;

constraint node22 partial = Hydra Candelabrum Hydractinia Ectopleura Clytia Obelia Physalia Craseoa Abylopsis Agalma Nanomia : Aeginia Halitrephes Haliclystus Lucernaria Calvadosia Alatina Chironex Morbakka Atolla Stephanoscyphus Nausithoe Aurelia Cassiopea Rhizostoma Nematostella Anemonia Aiptasia Metridium Antipathes Ceriantharia Corynactis Montastraea Porites Acropora Parazoanthus Anthomastus Leptogorgia Virgularia Choanoflagellata Demospongae Homoscleromorpha Calcarea Hexactinellida Placozoa Euplokamis Mnemiopsis Beroida Platyctenida Cestida Ganeshida Thalassocalycida Bryozoa Brachiopoda Phoronida Mollusca Annelida Echinodermata Pterobranchia Chaetognatha Onychophora Arthropoda Nemertea Enteropneusta Urochordata Cephalochordata Vertebrata Entoprocta Nematoda Nematomorpha Priapulida Gastrotricha Tardigrada Kinorhyncha Loricifera Gnathifera Xenacoelomorpha Platyhelminthes ;

constraint node23 partial = Hydra Ectopleura Candelabrum : Clytia Obelia Physalia Craseoa Abylopsis Agalma Nanomia HydractiniaAeginia Halitrephes Haliclystus Lucernaria Calvadosia Alatina Chironex Morbakka Atolla Stephanoscyphus Nausithoe Aurelia Cassiopea Rhizostoma Nematostella Anemonia Aiptasia Metridium Antipathes Ceriantharia Corynactis Montastraea Porites Acropora Parazoanthus Anthomastus Leptogorgia Virgularia Choanoflagellata Demospongae Homoscleromorpha Calcarea Hexactinellida Placozoa Euplokamis Mnemiopsis Beroida Platyctenida Cestida Ganeshida Thalassocalycida Bryozoa Brachiopoda Phoronida Mollusca Annelida Echinodermata Pterobranchia Chaetognatha Onychophora Arthropoda Nemertea Enteropneusta

Urochordata Cephalochordata Vertebrata Entoprocta Nematoda Nematomorpha Priapulida Gastrotricha Tardigrada Kinorhyncha Loricifera Gnathifera Xenacoelomorpha Platyhelminthes ;

constraint node24 partial = Hydractinia Clytia Obelia Physalia Craseoa Abylopsis Agalma Nanomia : Hydra Ectopleura Candelabrum Aeginia Halitrephes Haliclystus Lucernaria Calvadosia Alatina Chironex Morbakka Atolla Stephanoscyphus Nausithoe Aurelia Cassiopea Rhizostoma Nematostella Anemonia Aiptasia Metridium Antipathes Ceriantharia Corynactis Montastraea Porites Acropora Parazoanthus Anthomastus Leptogorgia Virgularia Choanoflagellata Demospongae Homoscleromorpha Calcarea Hexactinellida Placozoa Euplokamis Mnemiopsis Beroida Platyctenida Cestida Ganeshida Thalassocalycida Bryozoa Brachiopoda Phoronida Mollusca Annelida Echinodermata Pterobranchia Chaetognatha Onychophora Arthropoda Nemertea Enteropneusta Urochordata Cephalochordata Vertebrata Entoprocta Nematoda Nematomorpha Priapulida Gastrotricha Tardigrada Kinorhyncha Loricifera Gnathifera Xenacoelomorpha Platyhelminthes ;

constraint node25 partial = Clytia Obelia Physalia Craseoa Abylopsis Agalma Nanomia : Hydractinia Hydra Ectopleura Candelabrum Aeginia Halitrephes Haliclystus Lucernaria Calvadosia Alatina Chironex Morbakka Atolla Stephanoscyphus Nausithoe Aurelia Cassiopea Rhizostoma Nematostella Anemonia Aiptasia Metridium Antipathes Ceriantharia Corynactis Montastraea Porites Acropora Parazoanthus Anthomastus Leptogorgia Virgularia Choanoflagellata Demospongae Homoscleromorpha Calcarea Hexactinellida Placozoa Euplokamis Mnemiopsis Beroida Platyctenida Cestida Ganeshida Thalassocalycida Bryozoa Brachiopoda Phoronida Mollusca Annelida Echinodermata Pterobranchia Chaetognatha Onychophora Arthropoda Nemertea Enteropneusta Urochordata Cephalochordata Vertebrata Entoprocta Nematoda Nematomorpha Priapulida Gastrotricha Tardigrada Kinorhyncha Loricifera Gnathifera Xenacoelomorpha Platyhelminthes ;

constraint node26 partial = Physalia Craseoa Abylopsis Agalma Nanomia : Clytia Obelia Hydractinia Hydra Ectopleura Candelabrum Aeginia Halitrephes Haliclystus Lucernaria Calvadosia Alatina Chironex Morbakka Atolla Stephanoscyphus Nausithoe Aurelia Cassiopea Rhizostoma Nematostella Anemonia Aiptasia Metridium Antipathes Ceriantharia Corynactis Montastraea Porites Acropora Parazoanthus Anthomastus Leptogorgia Virgularia Choanoflagellata Demospongae Homoscleromorpha Calcarea Hexactinellida Placozoa Euplokamis Mnemiopsis Beroida Platyctenida Cestida Ganeshida Thalassocalycida Bryozoa Brachiopoda Phoronida Mollusca Annelida Echinodermata Pterobranchia Chaetognatha Onychophora Arthropoda Nemertea Enteropneusta

Urochordata Cephalochordata Vertebrata Entoprocta Nematoda Nematomorpha Priapulida Gastrotricha Tardigrada Kinorhyncha Loricifera Gnathifera Xenacoelomorpha Platyhelminthes ;

constraint node27 partial = Craseoa Abylopsis Agalma Nanomia : Physalia Clytia Obelia Hydractinia Hydra Ectopleura Candelabrum Aeginia Halitrephes Haliclystus Lucernaria Calvadosia Alatina Chironex Morbakka Atolla Stephanoscyphus Nausithoe Aurelia Cassiopea Rhizostoma Nematostella Anemonia Aiptasia Metridium Antipathes Ceriantharia Corynactis Montastraea Porites Acropora Parazoanthus Anthomastus Leptogorgia Virgularia Choanoflagellata Demospongae Homoscleromorpha Calcarea Hexactinellida Placozoa Euplokamis Mnemiopsis Beroida Platyctenida Cestida Ganeshida Thalassocalycida Bryozoa Brachiopoda Phoronida Mollusca Annelida Echinodermata Pterobranchia Chaetognatha Onychophora Arthropoda Nemertea Enteropneusta Urochordata Cephalochordata Vertebrata Entoprocta Nematoda Nematomorpha Priapulida Gastrotricha Tardigrada Kinorhyncha Loricifera Gnathifera Xenacoelomorpha Platyhelminthes ;

constraint node28 partial = Craseoa Abylopsis : Agalma Nanomia Physalia Clytia Obelia Hydractinia Hydra Ectopleura Candelabrum Aeginia Halitrephes Haliclystus Lucernaria Calvadosia Alatina Chironex Morbakka Atolla Stephanoscyphus Nausithoe Aurelia Cassiopea Rhizostoma Nematostella Anemonia Aiptasia Metridium Antipathes Ceriantharia Corynactis Montastraea Porites Acropora Parazoanthus Anthomastus Leptogorgia Virgularia Choanoflagellata Demospongae Homoscleromorpha Calcarea Hexactinellida Placozoa Euplokamis Mnemiopsis Beroida Platyctenida Cestida Ganeshida Thalassocalycida Bryozoa Brachiopoda Phoronida Mollusca Annelida Echinodermata Pterobranchia Chaetognatha Onychophora Arthropoda Nemertea Enteropneusta Urochordata Cephalochordata Vertebrata Entoprocta Nematoda Nematomorpha Priapulida Gastrotricha Tardigrada Kinorhyncha Loricifera Gnathifera Xenacoelomorpha Platyhelminthes ;

constraint node29 partial = Agalma Nanomia : Craseoa Abylopsis Physalia Clytia Obelia Hydractinia Hydra Ectopleura Candelabrum Aeginia Halitrephes Haliclystus Lucernaria Calvadosia Alatina Chironex Morbakka Atolla Stephanoscyphus Nausithoe Aurelia Cassiopea Rhizostoma Nematostella Anemonia Aiptasia Metridium Antipathes Ceriantharia Corynactis Montastraea Porites Acropora Parazoanthus Anthomastus Leptogorgia Virgularia Choanoflagellata Demospongae Homoscleromorpha Calcarea Hexactinellida Placozoa Euplokamis Mnemiopsis Beroida Platyctenida Cestida Ganeshida Thalassocalycida Bryozoa Brachiopoda Phoronida Mollusca Annelida Echinodermata Pterobranchia Chaetognatha Onychophora Arthropoda Nemertea Enteropneusta

Urochordata Cephalochordata Vertebrata Entoprocta Nematoda Nematomorpha Priapulida Gastrotricha Tardigrada Kinorhyncha Loricifera Gnathifera Xenacoelomorpha Platyhelminthes ;

Ctenosis (Ctenophore sister to all other animals):

constraint Ctenosis partial = Nematostella Anemonia Aiptasia Metridium Antipathes Ceriantharia Corynactis Montastraea Porites Acropora Parazoanthus Anthomastus Leptogorgia Virgularia Haliclystus Lucernaria Calvadosia Alatina Chironex Morbakka Atolla Stephanoscyphus Nausithoe Aurelia Cassiopea Rhizostoma Hydra Candelabrum Hydractinia Ectopleura Clytia Obelia Physalia Craseoa Abylopsis Agalma Nanomia Aeginia Halitrephes Demospongae Homoscleromorpha Calcarea Hexactinellida Placozoa Bryozoa Brachiopoda Phoronida Mollusca Annelida Echinodermata Pterobranchia Chaetognatha Onychophora Arthropoda Nemertea Enteropneusta Urochordata Cephalochordata Vertebrata Entoprocta Nematoda Nematomorpha Priapulida Gastrotricha Tardigrada Kinorhyncha Loricifera Gnathifera Xenacoelomorpha Platyhelminthes : Choanoflagellata Euplokamis Mnemiopsis Beroida Platyctenida Cestida Ganeshida Thalassocalycida;

Character list:

New characters or characters where we have changed the scoring strategy are indicted with an asterisk. Changes to previously existing characters might include corrected mistakes or changes in character contingencies.

1. Collar complex

    0 - absent

    1 - present

2. Multicellularity with extracellular matrix

    0 - absent

    1 - present

3. Septate junctions

0 - absent

1 - present

4. Tight junctions

0 - absent

1 - present

5. Gap junctions

0 - absent

1 - present

6. Adherens junctions

0 - absent

1 - present

7. Hemidesmosomes

0 - absent

1 - present

8. Epithelia* - Cell associations which line the exterior surface of an organism and are underlain by collagenous sheets. Sponges, while not typically possessing the sealing cell junctions found in the epithelia of other animals do possess cell junctions (characters 2-6) and so we score this character as being present in all animals.

0 - absent

1 - present

9. Differentiation of two epithelial layers* - This is present in all animals with a true epithelium except homoscleromorph sponges, which do not differentiate two epithelial layers[73].

0 - absent

1 - present

10. Basal laminae - We score this as present in absent in all sponges except homoscleromorphs[73]. All sponge Classes can possess epithelia underlain by collagenous sheets, but this is not true epithelia because they do not possess basal laminae. This is not the case in homoscleromorph sponges, which do possess epithelia with basal laminae, and so we consider them to possess a true epithelium.

    0 - absent

    1 - present

11. Aquiferous system with ostia and osculae* - A connected system of water channels found exclusively in sponges consisting of inhalent and exhalent openings, or ostia and osculae, respectively[74].

    0 - absent

    1 - present

12. Pinacocytes* - Cells[74] which characterise the external surface of a sponge.

    0 - absent

    1 - present

13. Archeocytes* - Amoeboid cells[74] found in sponges which retain totipotent capacity.

    0 - absent

    1 - present

14. Siliceous spicules produced by Silicateins* - Many metazoan groups possess spicule systems but amongst the animals, only sponges can produce siliceous spicules via the enzyme Silicatein. Crown-group calcareans do not produce siliceous spicules and silicateins have not been detected in homoscleromorph sponges[75].

    0 - absent

    1 - present

15. Spongin* - Derived from collagen, spongin is only found in demosponges[74].

0 - absent

1 - present

16. Collagen

0 - absent

1 - present

17. Nerve cells

0 - absent

1 - present

18. Acetylcholine used as a neurotransmitter

0 - absent

1 - present

19. Diffuse nervous system

0 - absent

1 - present

20. Giant axons associated with ciliary organs

0 - absent

1 - present

21. HOX/Para-HOX genes

0 - absent

1 - present

22. Epidermis with pulsatile bodies

0 - absent

1 - present

23. Xenacoelomorph cilia

0 - absent

1 - present

24. Diploblasts made of two cell layers

0 - absent

1 - present

25. Triploblasts made of three cell layers

0 - absent

1 - present

26. Striated ciliary rootlets

0 - absent

1 - present

27. Spiral cleavage with 4d mesoderm

0 - absent

1 - present

28. Colloblasts

0 - absent

1 - present

29. Coelenteron (gastrovascular cavity)

0 - absent

1 - present

30. Through-gut

0 - absent

1 - present

31. U-shaped gut

0 - absent

1 - present

32. Protonephridia (or homologous)

0 - absent

1 - present

33. Fate of blastopore

0 - protostomy

1 - deuterostomy

2 - amphistomy

34. Body cuticle with chitin

0 - absent

1 - present

35. Body cuticle with ?-chitin

0 - absent

1 - present

36. Body cuticle molted

0 - absent

1 - present

37. Segmented body with jointed limbs

0 - absent

1 - present

38. Lobopods

0 - absent

1 - present

39. Slime papillae

0 - absent

1 - present

40. Telescoping mouth cone with protrudable stylets

0 - absent

1 - present

41. Respiration via metameric trachaea and spiracles

0 - absent

1 - present

42. Mixocoel (haemocoel) surrounded by segmented mesoderm

0 - absent

1 - present

43. Teloblastic segmentation

0 - absent

1 - present

44. Longitudinal ventral nerve cord(s)

0 - absent

1 - present

45. Circum-pharyngeal, collar-shaped brain with anterior and posterior rings of perikarya separated by a ring-shaped neuropil

0 - absent

1 - present

46. Introvert with scalid rings

0 - absent

1 - present

47. Flosculi

0 - absent

1 - present

48. Immunoreactivity of horseradish peroxidase (HRP)

0 - absent

1 - present

49. Feeding strategy in tentaculate metazoans

0 - predominantly micro

1 - predominantly macro

50. Trochophores

0 - absent

1 - present

51. Segmental metanephridia sacculus

0 - absent

1 - present

52. Chitinous microvillar appendages

0 - absent

1 - present

53. Parapodia with dorsal and ventral branches terminated by ?-chitinous chaetae

0 - absent

1 - present

54. Radula

0 - absent

1 - present

55. Eversible proboscis surrounded by rhynchocoel

0 - absent

1 - present

56. Complex jaw apparatus in pharynx

0 - absent

1 - present

57. Origin of mesoderm

0 - from the blastopore lips and as ectomesoderm

1 - from the walls of the archenteron or neural crest

58. Radial cleavage

0 - absent

1 - present

59. Coelom formation

0 - schizocoely

1 - enterocoely

60. Trimeric coelom

0 - absent

1 - present

61. Pharyngeal slits

0 - absent

1 - present

62. Endostyle (or homologous)

0 - absent

1 - present

63. Notochord

0 - absent

1 - present

64. Stomochord

0 - absent

1 - present

65. Haemal system with axial complex

0 - absent

1 - present

66. Calcareous endoskeleton composed of separate ossicles

0 - absent

1 - present

67. Tornaria type larva

0 - absent

1 - present

68. Longitudinal dorsal nerve cord

0 - absent

1 - present

69. Zig zag myomeres

0 - absent

1 - present

70. Endothelium that lines the inner wall of blood vessels

0 - absent

1 - present

71. Neural crest

    0 - absent

    1 - present

72. Neurogenic placodes

    0 - absent

    1 - present

73. Dorsoventral axis

    0 - absent

    1 - present

74. Anterior posterior axis

    0 - absent

    1 - present

75. Number of principal body axes* - There are concerns about body axis homology between animal lineages, and we are unable to observe particular body axes (which are developmentally defined) in fossil taxa, but sponges and placozoans have one principal body axes, cnidarians and ctenophores have two and bilaterians three. We can assess fossils against these character states.

    0 - one

    1 - two

    2 - three

76. Compression in pharyngeal plane

    0 - absent

    1 - present

77. Symmetry

0 - bilateral

1 - biradial

2 - triradial

3 - tetraradial

4 - pentaradial

5 - hexaradial

78. Compression in oral aboral axis

0 - absent

1 - present

79. Compression in tentacular plane

0 - absent

1 - present

80. Mesoglea - Mesoglea, as found in cnidarians and ctenophores is scored as homologous to the mesohyl of sponges (following[39] but not Zhao *et al.*[8]

0 - absent

1 - present

81. Cells in mesoglea - Cells, including skeletal elements, fibrils and amoebocytes, are found in the mesoglea of sponges, ctenophores and some cnidarians (anthozoans and scyphozoans) (following[39])

0 - absent

1 - present

82. Cydippid larvae

0 - absent

1 - present

83. Ciliary rosettes

0 - absent

1 - present

84. Lophophore

0 - absent

1 - present

85. Radially-arranged outgrowths from the interface between the oral and aboral regions

0 - absent

1 - present

86. Radial outgrowths fixed in globular configuration

0 - absent

1 - present

87. Radial outgrowths tentacular

0 - absent

1 - present

88. Outgrowths with pinnules

0 - absent

1 - present

89. Outer sheaths on external surface of radial outgrowths

0 - absent

1 - present

90. Outgrowths with ciliary rows

0 - absent

1 - present

91. Cushion rings or polster cells

0 - absent

1 - present

92. Cushion rings paired

0 - paired

1 - unpaired

93. Ciliary rows paired

0 - paired

1 - unpaired

94. Large compound cilia

0 - absent

1 - present

95. Large cilia fused to form locomotory plate

0 - absent

1 - present

96. Orientation of ciliary rows

0 - adaxial

1 - abaxial

97. Uniformity of ciliary rows

0 - uniform

1 - non-uniform

98. Number of ciliary rows

0 - eight

1 - eighteen

2 - six

3 - twenty four

4 - more than twenty four

5 - sixteen

99. Extension of the oral surface to form oral cone

0 - absent

1 - present

100. Aboral region represented only by apical organ

0 - absent

1 - present

101. Apical organ forming narrow pointed extension

0 - absent

1 - present

102. Gut extending into aboralmost body region

0 - absent

1 - present

103. Stalk extending from apical/aboral region in polyp

0 - absent

1 - present

104. Oral macrocilia

0 - absent

1 - present

105. Oral lobes

0 - absent

1 - present

106. Morphology of ip of oral extension

0 - narrow

1 - voluminous

2 - manubrium like

3 - as margin of creeping sole

107. Mouth as margin of creeping sole

0 - absent

1 - present

108. Pharyngeal ridges

0 - absent

1 - present

109. Sealing ridges of pharynx

0 - absent

1 - present

110. Macrocilia on pharynx lining inside of mouth

0 - absent

1 - present

111. Ciliary dome

0 - absent

1 - present

112. Statolith

0 - absent

1 - present

113. Balancers

0 - absent

1 - present

114. Pole plates

0 - absent

1 - present

115. Aboral papillae

0 - absent

1 - present

116. Ciliary grooves

0 - absent

1 - present

117. Apical capsule

0 - absent

1 - present

118. Trifurcating ciliary grooves

0 - absent

1 - present

119. Interplate ciliary groove (ICG)

0 - absent

1 - present

120. Pharyngeal canals

0 - absent

1 - present

121. Tentacular canals

0 - absent

1 - present

122. Meridional canals

0 - absent

1 - present

123. Diverticula of meridional canals

0 - absent

1 - present

124. Circumoral ring canal

0 - absent

1 - present

125. Termination of meridional and pharyngeal canals

0 - both terminate blindly

1 - branch to form complex network

2 - united with circumoral ring canal

126. Interradial canals

0 - absent

1 - present

127. Number of interradial canals

0 - two

1 - four

128. Adradial canals

0 - Directly branch from the infundibulum

1 - fringing along elongate oral margin

129. Aboral canals

0 - absent

1 - present

130. Anal canals and pores - This character was split in[8] but we consider the presence of anal pores as contingent on the presence of anal canals and so have combined it.

0 - absent

1 - present

131. Paired ctenophore tentacles

0 - absent

1 - present

132. Tentilla

0 - absent

1 - present

133. Disposition of tentilla

0 - fringing along tentacles

1 - fringing along elongate oral margin

134. Tentacle sheaths

0 - absent

1 - present

135. Opening position of tentacles

0 - absent

1 - present

136. Auricles

0 - absent

1 - present

137. Brood chambers

0 - absent

1 - present

138. Skeletal elements (sclerotised arms and calyx)

0 - absent

1 - present

139. Skeletonisation restricted to aboral organ

0 - absent

1 - present

140. Tentacles extending beyond skeletal rods and outer sheaths

0 - absent

1 - present

141. Medial structures of skeletal elements

0 - absent

1 - present

142. Kinked spokes

0 - absent

1 - present

143. Spinose spokes

0 - absent

1 - present

144. Radiating flaps/lobes

0 - absent

1 - present

145. Circumferential oral constriction

0 - absent

1 - present

146. Constriction type

0 - skirt or bell

1 - lappets

147. Extracellular digestion

0 - absent

1 - present

148. Ostia with porocytes

0 - absent

1 - present

149. Embryonic development

0 - absent

1 - present

150. Cnidae

0 - absent

1 - present

151. Cnidae in gastrodermis

0 - absent

1 - present

152. Cnidocil

0 - immobile

1 - mobile

153. Structure of mitochondrial DNA

0 - circular

1 - linear

154. Scyphopharynx* - muscular pharynx as an extension of the oral cone present in Scyphozoa and Hydrozoa. Hydrozoans are often described as possessing a hypostome, but this is muscular[76] and the muscle forms a sphincter, which is unlike the true scyphopharynx (e.g.[77]) in which muscle is restricted and does not extend to the oral disc or form a sphincter. The actinopharynx is part of the digestive system and is not an extension of the oral cone and so these structures are not homologous. Cubozoans do not possess a pharynx[78] and it is not clear if staurozoans possess a muscular pharynx. There is some data showing the presence of a hypostome[79], but we are not able to establish whether this is muscular or not. Coronate scyphozoans do not appear to have a scyphopharynx[80,81].

0 - absent

1 - present

155. Hypostome* - This is an extension of the oral cone that functions as a muscular pharynx. This character is present in hydrozoans[76] and may be present in staurozoans but we were not able to establish whether the hypostome is muscular or not[79].

156. Actinopharynx - *Ceriantharia* was previously scored as absent for this character[8,39], but is here scored as present following its identification in many studies, e.g.[82]. This character was previously scored as absent in poriferans and placozoans, but we have changed this to non-applicable because the possession of an actinopharynx is contingent on possession of a gut.

0 - absent

1 - present

157. Siphonoglyph (sulcus) - *Ceriantharia* was scored as absent for this character[8,39], but is here scored as present following its identification in many studies, e.g.[82]

0 - absent

1 - present

158. Circular depression on basal disc

0 - absent

1 - present

159. Giant fiber nerve net (GFNN) in medusae - This character was duplicated in Zhao *et al.*[8] and so we have deleted the alternate version.

0 - absent

1 - present

160. Stenoteles

0 - absent

1 - present

161. Euryteles

0 - absent

1 - present

162. Desomonemes

0 - absent

1 - present

163. Mastigophores

0 - absent

1 - present

164. Basitrichousisorhizas

0 - absent

1 - present

165. Apotrichousisorhizas

0 - absent

1 - present

166. Isorhizas

0 - absent

1 - present

167. Heterotrichousanisorhizas

0 - absent

1 - present

168. Birhopaloids

0 - absent

1 - present

169. Rhopalonemes

0 - absent

1 - present

170. Spirocyst

0 - absent

1 - present

171. Ptychocyst

0 - absent

1 - present

172. Polyp tentacles* - Tentacles take different forms between anthozoan and medusozoan cnidarians. Anthozoan tentacles are generally short and stout, while medusozoan tentacles are long and filiform[83].

0 - filiform

1 - thick

173. Two tentacle polyp stage* - Cubozoa[67] and some Staurozoa[79,84] go through a two-tentacle polyp stage . This is not a feature seen in either scyphozoan or hydrozoan polyps and is not known to us in anthozoan polyps (e.g.[85])

0 - absent

1 - present

174. Periderm - Previously this character only encompassed chitinous skeletons, but the presence of chitins and proteins may vary independently of one another[86] and so we have rescored this character to include *Hydra*.

0 - absent

1 - present

175. Ridges or peridermal teeth - This character is contingent on producing a tube-encasing periderm. Living scyphozoans all produce peridermal teeth, as does *Eoconularia, Olivooides* and *Quadrapyrgites*[36,40,69]. Additionally, this was previously mis-scored as absent in taxa which lack a periderm.

0 - absent

1 - present

176. Ridges or peridermal teeth style* - Living coronates have continuous peridermal teeth, but this character is more variable in fossil groups. Many conulariids do not have peridermal teeth at all[41], but *Eoconularia* displays intermittent/irregular teeth[69] while *Olivooides*[36] and *Quadrapyrgites*[40] have one ring of teeth each. This character is altered from Leme *et al.* (2008)[41], who label the first state as 'single pair', but we change this to 'Single ring'.

0 - Single ring

1 - intermittent

2 - continuous

177. Bicuspid or paired peridermal teeth* - This character is present in the fossil genera *Olivooides*[36] and *Quadrapyrgites*[40], but is absent in conulariids and living scyphozoans.

0 - absent

1 - present

178. Cuticle layers in periderm

0 - absent

1 - present

179. Chitin expression

0 - restricted to basal area or to podocysts

1 - forming a tube

2 - forming a thin cuticle

180. Tube extension

0 - tube encases polyp

1 - tube does not encase polyp

181. Periderm type

0 - corneous

1 - coriaceous

2 - fibrous

182. Periderm with chitin - This character is now scored following Mendoza-Becerril *et al.* (2016)[86]

0 - absent

1 - present

183. Periderm with proteins - This character is now scored following Mendoza-Becerril *et al.* (2016)[86]

0 - absent

1 - present

184. Periderm externally annulated - This character is present in living scyphozoans, all sampled conulariids, *Olivooides*, *Pseudooides*, *Quadrapyrgites* and *Carinachites* and the hydrozoans *Hydractinia*, *Clytia* and *Obelia* (unlike previous studies which have scored Hydrozoa as uniformly absent for this character), which show limited apical annulation[83]. The fossil of *Auroralumina* is incomplete proximally, and so we score this as unknown.

0 - absent

1 - present

185. External annulation confined to one area of widespread* - This character defines the annulation type of the annulated hydrozoans, which do not show annulation across the entire hydroid[83] and is unlike living or fossil medusozoans which - apart from *Auroralumina* - are annulated across their entire surface.

0 - absent

1 - present

186. Annulation type* - This is contingent on external annulation of periderm and captures the variation between living hydrozoans, scyphozoans and fossil taxa. *Olivooides, Quadrapyrgites* and *Carinachites* are scored as having vase-like annulation, following the reconstructions of Duan *et al.* (2017)[36], Liu *et al.* (2014)[40] and Han *et al.* 2018)[46], along with annulated hydrozoans. All conulariids and *Pseudooides* (following Duan *et al.* 2017[39]) are scored as having ribs, as with all living scyphozoans. The fossil of *Auroralumina* is incomplete and so we have to score this character as unknown. The distinction is based on the presence of clear interspace regions (ribs) or not (vase-like).

0 - ribs

1 - vase-like

187. Vase-like annulation smooth or angular* - Annulation may either reach a pointed apex, or appear as smooth semi-circles. *Olivooides* and *Quadrapyrgites* (following Dong *et al.* 2013[36] and Liu *et al* 2014[40]) are angular, like the hydrozoan *Ectopleura*, while other hydrozoans - *Clytia* and *Obelia* - have a smooth ornamentation.

0 - angular

1 - smooth

188. Ribbing as straight or arcuate* - Living scyphozoans and most ribbed fossil groups have straight ribbing, but *Carinachites* (following Han *et al.* 2018[46]) has arcuate ribs, which dip basally down the periderm.

0 - straight

1 - arcuate

189. Regularity of ribbing* - Conulariids (e.g.[68,69]) and hexangulaconulariids (e.g. [39]) have regular external ribbing, while *Carinachites*[46] have an approximately regular ribbing, though it is clearly not as organised as in the conulariids and hexangulaconulariids. This is in contrast to living scyphozoans, which have an irregularly ornamented periderm[87,88].

0 - irregular

1 - approaching regular

2 - regular

190. Nodes present as ornamentation?* - This character is derived from a more inclusive character in the matrix of Leme *et al.* (2008)[41], which consider the presence of nodes, tubercles and ribs on multistate character. We choose to split these states because it is not clear they are derived from the same original ornamentation and are, therefore, homologous. Nodes are present in the conulariid *Conularia*.

0 - absent

1 - present

191. Thorn-like projections?* - This is scored as present in *Carinachites,* following the reconstruction of Han *et al.* 2018[46], and as absent in all other peridermal organisms

0 - absent

1 - present

192. Periderm with faces* - This character is present in the fossil taxa *Olivooides*[36], *Pseudooides*[39], *Quadrapyrgites*[40], *Carinachites*[46], all sampled conulariids and *Auroralumina.* It is not found in extant cnidarians with peridermal tubes.

0 - absent

1 - present

193. Corner sulci* - Longitudinal invaginations in a tubicolous periderm. These are absent in living tubicolous medusazoans, but are present in *Auroralumina,* conulariids[68,69] and *Carinachites*[46].

0 - absent

1 - present

194. Deep corner sulci* - *Carinachites*[46], unlike conualriids and *Auroralumina*, has corner sulci that are deeper than the central area of the periderm is wide. This character is unknown in *Auroralumina* because the raised central ridge in the periderm only represents a minimum estimate of sulcus height.

0 - absent

1 - present

195. Periderm with faces additional to corners - Hexangulaconulariids may have many faces additional to corners. *Pseudooides* has six faces, but *Septuconularia* is described as possessing fourteen faces[38], while conulariids have interadii marking a midline between corner sulci.

0 - absent

1 - present

196. Inflection (adapertural arching) of the transverse ornament at non-sulcate face margins* - Character adapted from and following[41]. *Olivooides*[36], *Quadrapyrgites*[40] and *Pseudooides*[39] are all scored as absent. This character is not applicable for fossil taxa either lacking external peridermal ornament altogether, or for those which lack transverse ornament.

0 - absent

1 - present

197. Continuation of the transverse facial ornament across the sulcate faces* - Character adapted from scored following Leme *et al.* (2008)[41]. This is scored as absent in *Auroralumina,* and non-applicable in those groups which lack sulcate corners.

0 - absent

1 - present

198. External peridermal ornamentation at the non-sulcate corners* - Character adapted from Leme *et al.* (2008)[41] and scored accordingly. This is scored as unknown in *Pseudooides* and inapplicable in other fossil forms as they do not have non-sulcate corners.

0 - absent

1 - present

199. Ornament regular* - This is a new character which describes the external ornament of included fossil taxa to the exclusion of extant scyphozoans which have an irregular external ornament, which corresponds to their more irregular shape, e.g.[89].

0 - absent

1 - present

200. Transverse cross section of the corner sulcus* - Character from Leme *et al.* (2008)[41] and scored accordingly. *Auroralumina* and *Carinachites*[46] are additionally scored as having angulated corner sulci.

0 - rounded

1 - angulated

2 - angulated with fold

201. Crests - longitudinal ridges in peridermal corrugated interspaces* - Character from Leme *et al.* (2008)[41] and scored accordingly. This character is additionally scored as absent in, *Olivooides*[36] , *Quadrapyrgites*[40] and *Pseudooides*[39].

0 - absent

1 - present

202. Periderm with lobate aperature* - aperture marked by sub-triangular oral lobes are present in *Conularia*[69], *Carinachites*[46], *Olivooides*[36] and *Quadrapyrgites*[40]. They are absent in living scyphozoans and hydrozoans.

0 - absent

1 - present

203. Periderm with differentiation into stalk and cup* - This is a new character and defines the anatomy of *Auroralumina.* All other sampled animals with peridermal tubes have a single tube with no distinction between zones.

0 - absent

1 - present

204. Periderm tube tapering* - This is a new character and is found in living coronate scyphozoans as well as the fossil conulariids[41], *Olivooides*[36], *Quadrapyrgites*[40], *Pseudooides*[39] and. It is absent in living hydrozoans and the fossils *Carinachites* and *Auroralumina*.

0 - absent

1 - present

205. Steeply pyramidal taper* - This is present in conulariids[41] and *Pseudooides*[39], but is absent in *Olivooides* and *Quadrapyrgites*.

0 - absent

1 - present

206. Transverse cross section of the oral region of the polyp* - Character from and following Leme *et al.* (2008)[41]. This is also scored as polyhedral in all sampled fossil groups and circular in both living coronate scyphozoans and hydrozoans.

0 - circular

1 - polyhedral

207. Location of medusa formation - We score *Olivooides* as apical or oral here because of the associated presence of ephyae[36], and have all other fossils as unknown because here is no compelling case to say whether any of these fossil groups produce medusae.

0 - lateral budding from an entocodon

1 - apical or oral

2 - direct development without polyp stage

208. Type of apical medusa formation* - Conulariids have been changed from ‘0, strobilating’, as was scored in Zhao *et al.* (2019)[8], to ‘?’ to reflect uncertainty in the strength of the evidence for strobilation[44] and, indeed, uncertainty as to whether conulariids produced medusae at all. *Olivooides* is scored as present because ephyrae as known in this taxon (following Dong *et al.* 2013[36]).

0 - Strobilation

1 - Without transverse fission

209. Strobilation type* - The cubozoan *Morbakka*, added in this manuscript, displays monodisc stroblation[90]. The presence of two fused ephyae associated with *Olivooides* suggests the presence of polydisk strobilation[36].

0 - polydisk

1 - monodisk

210. Polyp propagation through lateral budding

0 - absent

1 - present

211. Oocyte development

0 - oocytes develop without accessory cells

1 - oocytes develop with accessory cells

2 - oocytes develop within follicles

3 - oocytes develop from uptake of somatic or other germ line cells

212. Nectosome

0 - absent

1 - present

213. Pneumatophore

0 - absent

1 - present

214. Planula

0 - absent

1 - present

215. Feeding planula

0 - absent

1 - present

216. Planula ciliation

0 - absent

1 - present

217. Number of ectodermal cells on the planula

0 - absent

1 - present

218. Glandular cells in the planula

0 - absent

1 - present

219. Nervous cells in the planula

0 - absent

1 - present

220. Relationship between axes of planula and adult

0 - oral-aboral axis in the adult derived from the longitudinal axis of the planula

1 - oral-aboral axis in the adult derived from the transverse axis of the planula

221. Polypoid phase - nb. *Halitrephes* and *Aegina* which do not have polypoid stages were previously scored as present for some characters contingent on the presence of a polypoid phase. These characters have been changed so all polyp-contingent characters in *Halitrephes* and *Aegina* are scored as inapplicable.

0 - absent

1 - present

222. Polyp size class* - new character to characterise the difference between medusozoan polyps and anthozoans polyps. Sampled medusozoan polyps are on the order of a few mm, with the exclusion of the larger hydrozoans. There are reports of giant deep sea hydrozoan polyps which can reach meters in height, but these seem to be highly derived as compared to the rest of the group. Conulariids are on the order of a few cm, while *Carinachites* is a few mm. Given the nature of the fossil material it is not possible to discern the total height of *Olivooides, Quadrapyrgites* or *Pseudooides. Auroralumina* is an obvious outlier, with a polyp height of tens of cm. These data are represented in Extended data Figure 7.

0 - a few mm

1 - a few cm

2 - tens of cm

223. Polyp organisation

0 - solitary

1 - colonial

224. Colony type* - new character to characterise the differences in colony morphology. *Auroralumina* and all colonial anthozoans (e.g. gorgonians) are arborescent while there is greater variability in medusozoans. Scyphozoans are scored as having arborescent and random colony organisation[87,88], while hydrozoans show arborescent (*Candelabrum*[91]) creeping (e.g. *Hydractinia*[92]) and siphonophore-type colonies.

0 - arborescent

1 - creeping

2 - siphonophore-type

3 - random

225. Oral disc in polyp* - New character defining a rounded oral structure surrounded by tentacles and opening into a pharynx. This is present in the polypoid fossils *Xianguangia* and *Daihua*[8] as well as in all anthozoans, scyphozoans, cubozoans and hydrozoans (excluding siphonophores which have reduced polyps).

0 - absent

1 - present

226. Coenenchyme* - this is a new character. Coenenchyme is mesoglea that has been penetrated by solenia (tubes) and is common in binding together colonial anthozoans. This is scored as present in colonial anthozoans and *Cambroctoconus* (following Peel 2017) and non-applicable in all solitary cnidarians and colonial medusozoan polyps.

0 - absent

1 - present

227. Mineralised coenenchyme* - This is present in *Cambroctoconus* (following Peel 2017[53]), but is absent in living anthozoans.

0 - absent

1 - present

228. Polymorphic polyps

0 - absent

1 - present

229. Number of tentaculate whorls

0 - one

1 - two or more

230. Gut partitioned by septa

0 - absent

1 - present

231. Septa in polyp - This was previously miscored as present in cubozoans[8], when they are absent[93].

0 - absent

1 - present

232. Septa in medusa

0 - absent

1 - present

233. Mesoglea in polyp septa

0 - absent

1 - present

234. Eightfold organisation of septa* - This is present in *Cambrotoconus*[53,94] and living octocorals.

0 - absent

1 - present

235. Only four gastric septa present* - this is a character that defines living medusozoans[90,93,95] and is present in *Eoconularia* after Jerre (1994)[69].

0 - absent

1 - present

236. Pairing of mesenteries?* - this is now scored as absent in *Xianguangia* on the strength of data presented in Ou *et al.* (2017)[96] and Zhao *et al.* (2019)[8].

0 - absent

1 - present

237. Pairing of mesenteries

0 - paired

1 - coupled

238. Pair morphology

0 - members same size

1 - members different size

239. Paired secondary cycle - this is now scored as inapplicable in cubozoans (rather than absent) as they are absent for paired mesenteries (character 236).

0 - absent

1 - present

240. Mesenterial fusion - this is now scored as absent in scyphozoans, rather than inapplicable, because they possess gastric septa considered homologous to mesenteries in this dataset.

0 - absent

1 - present

241. Number of perfect mesenteries

0 - eight

1 - ten

2 - more than twelve

242. Types of mesentery

0 - only perfect mesenteries

1 - perfect mesenteries not divisible

2 - perfect mesenteries and imperfect mesenteries divisible

243. Directive mesentery - this is now scored as absent in scyphozoans, rather than inapplicable, because they possess gastric septa considered homologous to mesenteries in this dataset.

0 - absent

1 - present

244. Number of directive mesentery pairs

0 - one pair

1 - two pairs

245. Mesentery formation - This is changed from inapplicable in all medusozoans to unknown in staurozoans and scyphozoans as we assume gastric septa to be homologous to mesenteries.

0 - only primary tentacles

1 - new septa arise in the exocoel to either side of the ventral directive

2 - from ventral

3 - between directive and transverse septa

4 - anywhere around the circumference

246. Structure of polyp tentacles

0 - hollow

1 - solid

247. Type of tentacles

0 - one type

1 - two types

248. Clustered tentacles

0 - absent

1 - present

249. Two-tentacle polyp stage* - This character defines a developmental stage that living cubozoans[79] and staurozoans[90] go through, but living scyphozoans and hydrozoans and anthozoans do not. Previous iterations of this matrix only scored cubozoans as going through a two-tentacle polyp stage.

0 - absent

1 - present

250. Siphonoglyph count

0 - one

1 - more than one

251. Mesenteric filament

0 - absent

1 - present

252. Mesenteric filament type

0 - two strips

1 - three strips

2 - one strip

253. Embryonic stage retained in the tube morphology

0 - absent

1 - present

254. Tentacles retractile

0 - non retractile

1 - retractile

255. Tentacle/coelenteron relationship

0 - one tentacle per endocoel and per exocoel

1 - one tentacle per exocoel, multiple per endocoel

256. Catch tentacles

0 - absent

1 - present

257. Arrangement of tentacles - This character – from Zhao *et al.* (2019)[8] - has been split so that is now has a subordinate character (259) dealing with the number of cycles.

0 - scattered

1 - in cycles

258. Number of cycles

0 - one

1 - more than one

259. Acrospheres

0 - absent

1 - present

260. Number of tentacles in polyp

0 - six

1 - eight

2 - twelve

3 - sixteen

4 - eighteen

5 - more than twenty

261. Marginal spherules

0 - absent

1 - holotrichous

262. Acontia

0 - absent

1 - present

263. Gonads on mesenteries of 1st cycle

0 - absent

1 - present

264. Gonads on mesenteries on 2nd and subsequent cycles

0 - absent

1 - present

265. Mesogleal sphincter

0 - absent

1 - present

266. Ectodermal longitudinal muscle

0 - absent

1 - present

267. Ectodermal longitudinal muscle location

0 - tentacles and oral disc only

1 - whole body

268. Basilar musculature

0 - absent

1 - present

269. Retractor muscle

0 - weak

1 - defined

270. Parietal muscle

0 - absent

1 - present

271. Mesogleal lacunae

0 - absent

1 - present

272. Acrorhagi

0 - absent

1 - present

273. Pedal disk

0 - absent

1 - present

274. Ciliated tract on mesenteric filament

0 - absent

1 - present

275. Ephyrae

0 - absent

1 - present

276. Rhopalial lappets in ephyrae* - In many living scyphozoans, lappets flank the developing rhopalia[97], though this is absent in some forms such as *Nausithoe*[97]. Rhopalial lappets are absent in *Olivooides*, following the illustrations of Dong *et al.* (2013)[36].

0 - absent

1 - present

277. Number of marginal lappets* - This is a new character. In extant scyphozoans ephyrae display a number of lappets (which may house rhopalial lappets). The numbers vary in living scyphozonans, but the ephyrum of *Olivooides* has a unique condition of five marginal lappets[36] (Dong *et al.* 2013), presumably reflecting the pentaradial symmetry of the polyp stage[97].

0 - five

1 - eight

2 - ten

3 - sixteen

4 - seventeen or more

278. Velar lappets* - This is a new character. Velar lappets develop between rhopalial lappets in some scyphozoans, some coronates, e.g. *Nausithoe*, and all rhizostomes[97]. Velar lappets are absent in *Olivooides*, following the illustrations of Dong *et al.* (2013)[36].

0 - absent

1 - present

279. Gastro-vascular star-like in shape* - This is a new character and is present in all known living scyphozoan ephyrae[97], but it's unclear whether this character is present or absent in *Olivooides*.

0 - absent

1 - present

280. Actinula

0 - absent

1 - present

281. Organisation of nervous system

0 - not in bunches

1 - organised in bunches of gastrodermic origin

2 - organised in bunches of ectodermic origin

282. Complex ganglia associated with sensory structures in absence of CNS

0 - absent

1 - present

283. Canal system in polyp

0 - absent

1 - present

284. Gastrodermic musculature

0 - absent

1 - present

285. Medusoid phase

0 - absent

1 - present

286. Arms with cluster of secondary tentacles* - This is a new character defining the anatomy of the staurozoan bodyplan: the polyp and stauromedusae.

0 - absent

1 - present

287. Arms bifurcating* - this is a new character and defines the anatomy of the arms of *Calvadosia* to the exclusion of *Haliclystus* and *Lucernaria*[95].

0 - absent

1 - present

288. Primary tentacles modified to anchors* - this is a new character and is present in all three sampled staurozoans[95].

0 - absent

1 - present

289. Claustra* - This is a new character. This character defines a tissue in the gastrovascular cavity that is surrounded by mesoglea and gastrodermis and does not contact epidermis. Historically, cubozoans were described as having a claustrum, but recent work has shown that the 'claustrum' of cubozoans is actually a valve of the gastric ostium[98] and so we do not consider these structures homologous. Of our sampled taxa, only the staurozoan *Calvadosia* has a claustrum[95].

0 - absent

1 - present

290. Gonads in medusa calyx* - this is a new character which is present in *Haliclystus* and *Lucernaria* to the exclusion of *Calvadosia*[95] and other medusozoans, which do not have a calyx in the medusa stage.

0 - absent

1 - present

291. Longitudinal muscles in peduncle* - This a new character and is present in *Calvadosia* to the exclusion of other sampled staurozoans[95]. No other medusozoans have a peduncle and so are scores as inapplicable.

0 - absent

1 - present

292. Number of chambers in peduncle* - This is a new character, defining variability of peduncle type in living staurozoans. Both *Haliclystus* and *Lucernaria* have four chambers, while *Calvadosia* has one chamber[95]. No other medusozoans have a peduncle and so are scores as inapplicable.

0 - one

1 - four

293. Pad-like adhesive structures on tentacles derived from arms* - This is a new character, exploring tentacle variation in living staurozoans. Pad-like adhesives are present on the tentacles of *Haliclystus* and *Lucernaria*, but not *Calvadosia*[95]. No other medusozoans are known to have this character.

0 - absent

1 - present

294. Pedalium of coronate type

0 - absent

1 - present

295. Rhopalia - Staurozoans are rescored as absent for rhopalia and not non-applicable because they possess rhopaliods which are not true rhopalia[99].

0 - absent

1 - present

296. Complex eyes in rhopalia

0 - absent

1 - present

297. Nerve ring in medusa

0 - absent

1 - present

298. Number of rings

0 - one

1 - two

299. Gastric filaments

0 - absent

1 - present

300. Coronal muscle

0 - well developed

1 - marginal and tiny

301. Pedalium of the cubozoan type

0 - absent

1 - present

302. Velum

0 - absent

1 - present

303. Umbrellar margin

0 - smooth and continuous

1 - lobed

304. Tentacles in medusa

0 - absent

1 - present

305. Tentacular bulbs

0 - absent

1 - present

306. Statolith composition

0 - MgCaPO4

1 - CaSO4

307. Septal shape in medusa

0 - straight

1 - Y-shaped

308. Radial canals

0 - absent

1 - present

309. Circular canal

0 - absent

1 - present

310. Circular canal partial

0 - absent

1 - present

311. Velarium

0 - absent

1 - present

312. Coronal furrow

0 - absent

1 - present

313. Gonadal location

0 - manubrium

1 - radial canals

314. Statocysts

0 - absent

1 - present

315. Statocyst location

0 - endodermic

1 - ectodermic

316. Perradial mesenteries

0 - absent

1 - present

317. Adult medusoid shape

0 - bell

1 - pyramidal

2 - cubic

3 - actinuloid

318. Shape of horizontal cross-section of the medusa

0 - circular

1 – four-part symmetry

319. Urticant rings

0 - absent

1 - present

320. Oral arms with suctorial mouths

0 - absent

1 - present

321. Tentacular insertion

0 - umbrellar margin

1 - away from margin

322. Manubrium

0 - absent

1 - present

323. Nervous system organisation

0 - GFNN absent

1 - GFNN present

324. Structure of medusa tentacles

0 - hollow

1 - solid

325. Tentacular morphology

0 - straight

1 - straight with angular inflection

326. Peronia

0 - absent

1 - present

327. Ocelli in medusa

0 - absent

1 - present

328. Peripheral system

0 - absent

1 - present

329. Umbrellar furrow

0 - absent

1 - present

330. Development of the umbrella

0 - fully developed

1 - aboral cone

331. Number of tentacular whorls

0 - one whorl

1 - two whorls

332. Velar canals

0 - absent

1 - present

333. Frenulae

0 - absent

1 - present

334. Shape of medusa tentacles

0 - filliform

1 - capitate

335. Zooxanthellae

0 - absent

1 - present

336. Ectodermal skeleton

0 - absent

1 - present

337. Composition of ectodermal skeleton

0 - proteinaceous

1 - calcitic

338. Mesogleal skeleton

0 - absent

1 - present

339. Columella

0 - absent

1 - present

340. Costae

0 - absent

1 - present

341. Octocorallian spicules

0 - absent

1 - present

342. Spicules in tentacle

0 - absent

1 - present

343. Gorgonin

0 - absent

1 - present

344. Desmocytes

0 - absent

1 - present

345. Anthozoan desmocytes* - Desmocytes are specialised cells which bind soft tissues and skeletal structures in cnidarians, but there are ultrastructural variations between democytes in different cnidarian groups. Anthozoan desmocytes have a pectinate margin, ‘scyphozoan’ desmocytes form a recessed pit (character 346), and hydrozoan desmocytes (character 347) become embedded in secretory products of adjacent cells[100]. Tibdall (1982) only finds analogy between desmocytes, because of significant anatomical and developmental differences. However, the junctions they mediate (e.g. hemidesmosomes) are only found in cnidarians, so we view it as likely that they arose from a common ancestor. Therefore, we score these types of desmocytes as independent characters, but inapplicable in all non-cnidarian lineages.

0 - absent

1 - present

346. ‘Scyphozoan’ desmocytes

0 - absent

1 - present

347. Hydrozoan desmocytes

0 - absent

1 – present

#NEXUS

Begin DATA;

Dimensions ntax=104 nchar=347;

Format datatype=standard interleave=yes gap=- missing=?;

MATRIX

Choanoflagellata 10---------000000---0-0--0------------------------0-------------------
000------00----------0----------------0000-------0-0--?---0------0-----0--0--00-0-------------------0-
------------------?----------------?---------0--0-
---------------------------??-------------------0-0--0----??

Demospongae 1100010100111(0 1)110---0000-0000--0----00000-0--000-
0000000-0---000000-----0?0----110000--------0---------------0000-------0-0-0----0----000-----
00-01100-0-------------------0------------------?---------------?--0------0----------------------------
---------------------------------0---------------------------??------------------0-0--0----??

Homoscleromorpha 11000101011110010---0000-0000--0----00000-0--000-
0000000-0---000000-----0?0----110000--------0---------------0000-------0-0-0----0----000-----
00-01100-0-------------------0------------------?---------------?--0------0----------------------------
---------------------------------0---------------------------??------------------0-0--0----??

Calcarea 11100101001110010---0000-0000--0----00000-0--000-0000000-0--
-000000-----0?0----110000--------0----------------0000-------0-0-0----0----000-----00-01100-1--
------------------0-----------------------------------?--0------0---
------------------0---------------------------??--------------------0-0--0------

Hexactinellida 11000101001111010---0000-0000--0----00000-0--000-0000000-
0---000000-----0?0----110000--------0----------------0000-------0-0-0----0----000-----00-01100-
0--------------------0-----------------------------------?--0------0---
---------------------0---------------------------??--------------------0-0--0------

Placozoa 01?00101100000010---1000-1000--0-0--00000-0--000-0000000-0--
-000000-----0?0----??0000--------0---------00-----0000-----0-0-0-0----0----000-----0--1000--0--
------------------0-----------------------------------?---------0---
----------------0---------------------------??--------------------0-0--0------

Xianguangia ??????????0??0?????1???????010-?????00000?0??0000??00000-
???0?0000??0???0?1???-?????1011?10-01000100-10?0-0?????????0???-?????????0----
0?10100000-10?0--?001?1?????????????1?0---------------------------------??0??????????1?0-1--
0011??000---
???????0?0????0??10?4????????????0??
?????????????????????????

Daihua ??????????0??0?????1????????10-?????00000?0??00?0??00000-
???0?0000??0???0?1?0?-?????1011111001000100-11?0-
0??????0??0?????????????0????0?10100000-10?0???001???????????????1?0------------------
--------------??0??????????1?0-1--
0011??0????????????0?0????0??10?4????????????0??????????????????????????????????????
???????????????????????????????????????

Dinomischus_venustus ??????????0??0?????1???????010-
?????00000?0??00?0??00000-???0?0000??0???0?1???-?????1011111001000110-
11?000?????????0???-?????????0----0?10?00000-10?0???000???????????????1?0--------------
------------------??0??????????1?0-0--

0011??0????????????0?0????0??10?4????????????0?????????????????????????????????????
?????????????????????????????????????

Siphusauctum ??????????0??0?????????????010-?????00000?0??00?0??00000-
???0?0000??0???0?1?5?-?????110111001000210-01?000?????????0???-
?????????0????0?0?----10-10?0???000?????????????????0--------------------------------
??0???????????1?0-0--
0000???????????????0?0????0??10?0????????????0????????????????????????????????????
?????????????????????????????????????

Namacalathus
??????????0??0???0????????1?5???????
???????????????0-
?1??????????????????????????????????10??00??????????????????????????????0---------------
----
??1??????????1??0??????????????????
???

Eolympia
??????????0??0??????????????10??????00?0??0????????00????????????0????????1?0??????
?100???????????00-?1??-??????????0??????????????0?---
??????????????????001?????????????????0--------------------------------??????????????1?0-0--
0011?????????0?????0?0???????10?4????????????0?????????????????????????????????????
?????????????????????????????????????

Fasciculus ??????????0??0?????1???????0????????00000?0??00????00000-
???0?0000??0???0?1?1?-?????11000?11???1141??00?0?0?????????????-?????????0----??0---
--00-???????????-?????????????--0--------------------------------??-
???????????????????????????????????--????????????-
????????????0??
???

Xanioascus ??????????0??0?????????????0????????00000?0??00????00000-???0?0000??0???0?1?1?-?????11000?11???10311000?0?0??????10?00??-?????????0----??0-----00-???????????-?????????????--0---------------------------------??-???????????????????????????????????--????????????-????????????0???

Ctenorhabdotus ??????????0??0?????????????0????????00000?0??00????00000-???0?0000??0???0?1?1?-?????11000?11???11311000?010???0??00111??-?????????0----0?11----010???????????-?????????????--0---------------------------------??-???????????????????????????????????--????????????-????????????0???

Gemmactena ??????????0??0?????????????????????00000?0??00????00000-???0?0000??0?????1???-?????11000?1?????00111?0?0?0???0?????0???-????????00----0?100110111???????????-?????????????--0---------------------------------??-???????????????????????????????????--????????????-????????????0???

Thaumactena ??????????0??0?????????????0????????00000?0??00????00000-???0?0000??0?????1???-?????11000?10????00111?0?0?0???0?????0???-?????????0----0?100?00110???????????-?????????????--0---------------------------------??-???????????????????????????????????--????????????-????????????0???

Galeactena ??????????0??0?????1???????0????????00000?0??00????00000-???0?0000??0???0?1???-?????11000?10????0011110?010???0?????0???-?????????0----0?100?00110???????????-?????????????--0---------------------------------??-???????????????????????????????????--????????????-

????????????0???
???

Batofasciculus ??????????0??0??????????????????????00000?0??00????00000-???0?0000??0?????1???-?????1100???0???100111?0?0?0???0?????0???-?????????0----0?100101111???????????-?????????????--0---------------------------------??-????????????????????????????????????--????????????-????????????0???
???

Maotianoascus ??????????0??0?????????????0????????00000?0??00????00000-???0?0000??0?????1???-?????11000?10????00110?0?0?0???0?????????-?????????0----0?100???110???????????-?????????????--0---------------------------------??-????????????????????????????????????--????????????-????????????0???
???

Trigoides ??????????0??0??????????????????????00000?0??00????00000-???0?0000??0?????1???-?????1100??1?????00111?0?0?0???0?????0???-?????????0----0?100110111???????????-?????????????--0---------------------------------??-????????????????????????????????????--????????????-????????????0???
???

Euplokamis 01001111110000011111000101011100-0-000000-00000010000000-0--00000000000001101011111011000111111100110000000000111101000111000101111101l000-----00-1000-?00000----------------0------------------------------------?---------0---0----0-----?---0--0-0--0------

Mnemiopsis 01001111110000011110000101011100-0-000000-00000010000000-0--0000000000000111100111101100011111111011000010000011110100111101211111111101

100-----00-1000-?00000----------------0-----------------------------------?---------0---0----0-----?--
---0---0-0--0------

Beroida 0100111111000001111000010100 1100-0-000000-
00000010000000-0--000000000000011110-
1101011000111111100110001010011111111 0001-11120-0000----000-----00-1000--00000---
-------------0---------------------------------?---------0---0----0-----?--------------------------------
--------------0---------------------------??------------------0-0--0------

Platyctenida 0100111111000001111000010101 1100-0-000000-
00000010000000-0--000000000000011111111111011000(0
1)111111?0110000000100011111110 00111(0 1)0110(0 1)1111011010-----00-1000--00000-----
-----------0-----------------------------------?---------0---0----0-----?----------------------------------
------------0---------------------------??------------------0-0--0------

Cestida 0100111111000001111000010101 1100-0-000000-
00000010000000-0--
00000000000000111100111101100011111111011000000000000111?010011110(0
1)21111111111000-----00-1000--00000----------------0-----------------------------------?---------
0---0----0-----?--0--------------------------??---------------
-----0-0--0------

Ganeshida 0100111111000001111000010101 1100-0-000000-
00000010000000-0--
00000000000000111100111101100011111110011000010000 0111?010011110(0
1)21111?11101000-----00-1000--00000----------------0-----------------------------------?---------
0---0----0-----?--0--------------------------??---------------
-----0-0--0------

Thalassocalycida 0100111111000001111000010101 1100-0-000000-
00000010000000-0--
0000000000000011110011110110001111111001100000200000111?010011110(0
1)21111?11101000-----00-1000--00000----------------0-----------------------------------?---------

0---0----0-----?--0----------------------------??---------------
-----0-0--0------

Bryozoa 0110111111000001110-10011110011100-
0000000000000000100000001000000000000011200--0-001(0 1)--------0----00-0000-
00000000000--0-0-0-0--000----000-----00-1010--00000----------------0----------------------------
--------?--0------0---0----0-----?--0---------------------------
-??--------------------0-0--0------

Brachiopoda 0110111111000001110-100111100(0 1)11(0 1)0-
0000000000000000100000001000000000000011200--0-0010--------0----00-0000-
00000000000--0-0-0-0--000----000-----00-1010--00000----------------0----------------------------
--------?--0------0---0----0-----?--0---------------------------
-??--------------------0-0--0------

Phoronida 0110111111000001110-10011110011100-
0000000000000000000000001000000000000011200--0-0010--------0----00-0000-
00000000000--0-0-0-0--000----000-----00-1010--00000----------------0----------------------------
--------?--0------0---0----0-----?--0---------------------------
-??--------------------0-0--0------

Mollusca 0110111111000001110-100111100101(0 1 2)(0
1)00000000010000110101000000000000000000011200--0-000(0 1)--------0----00-1000-
00000000000--0-0-0-0--000----000-----00-1010--00000----------------0----------------------------
--------?--0------0---0----0-----?--0---------------------------
-??--------------------0-0--0------

Annelida 0110111111000001110-100111100101(0 1 2)0-0000000110000(0
1)1011000000000000000000011200--0-0000--------0----00-1000-00000000000--0-0-0-0--
000----000-----00-1010--00000----------------0------------------------------------?--0------0---0----
0-----?--0----------------------------??--------------------0-0-
-0------

Echinodermata 0110011111000001110-10011100010110-0000000000000000000011111000111000001120 0--0-000(0 1)--------0----00-1(0 1)00-00000000000--0-0-0-0--000----000-----00-1010--00000---------------0---------------------------
--------?--0------0---0----0-----?---0--------------------------
-??--------------------0-0--0------

Pterobranchia 0110111111000001110-10011100011110-00000000100000000000011111101101000001120 0--0-0000--------0----00-1000-00000000000--0-0-0-0--000----000-----00-1010--00000---------------0---------------------------
--------?--0------0---0----0-----?---0--------------------------
-??--------------------0-0--0------

Chaetognatha 0110111111000001110-10011100010110-0000000010000-000000110010000000000000011200--0-0000--------0----00-1000-00000000000--0-0-0-0--000--
--000-----00-1000--00000---------------0-----------------------------------?---------0---0----0-----
?---0---------------------------??--------------------0-0--0----
--

Onychophora 0110111111000001110-100111000101211101101111 0001-0100000000000000000000011200--0-0000--------0----00-1000-00000000000--0-0-0-0--000--
--000-----00-1000--00000---------------0-----------------------------------?---------0---0----0-----
?---0---------------------------??--------------------0-0--0----
--

Arthropoda 0110111111000001110-100111000101(0 1)1111000(0 1)1110001-0100000000000000000000011200--0-0000--------0----00-1000-00000000000--0-0-0-0--000----000-----00-10{0 1}0--00000---------------0-----------------------------------?--0---
---0---0----0-----?---0---------------------------??------------
--------0-0--0------

Nemertea 0110111111000001110-100111100101(0 1)0-0000000010000-1000010000000000000000000011200--0-0000--------0----00-1000-00000000000--0-0-0-0--000--
--000-----00-1010--00000---------------0-----------------------------------?--0------0---0----0-----

?--0----------------------------??--------------------0-0--0----
--

Enteropneusta 0110111111000001110-10011100010110-0000000010000-0000000111111011010000011200--0-0000--------0----00-1000-00000000000--0-0-0-0--000----000-----00-1010--00000----------------0------------------------------------?--0------0---0----0-----
?--0----------------------------??--------------------0-0--0----
--

Urochordata 0111111111000001110-10011100010110-0000000000000-0000000101011100001001111200--0-0000--------0----00-1000-00000000000--0-0-0-0--000----000-----00-1010--00000----------------0------------------------------------?--0------0---0----0-----
?--0----------------------------??--------------------0-0--0----
--

Cephalochordata 0110111111000001110-10011100010110-0000000000000-0000000111011100001100011200--0-0000--------0----00-1000-00000000000--0-0-0-0--000----000-----00-1010--00000----------------0------------------------------------?--0------0---0----0-----
?--0----------------------------??--------------------0-0--0----
--

Vertebrata 0101111111000001110-10011100010110-0000000000000-00000001(0 1)101110000111111200--0-0000--------0----00-1000-00000000000--0-0-0-0--000----000-----00-1000--00000----------------0------------------------------------?---------0---0----0-----?--0----------------------------??--------------------0-0--0------

Entoprocta 0110111111000001110-100111100111010000000-0000000100000000--00000000000011200--0-0001--------0----00-1100-00000000000--0-0-0-0--000----000-----00-1010--00000----------------0------------------------------------?--0------0---0----0-----?--0----------------------------??--------------------0-0--0------

Nematoda 0110111111000001110-100111000101010100000-011101-
000000000--00000000000011200--0-0000--------0----00-1000-00000000000--0-0-0-0--000---
-000-----00-1000--00000---------------0----------------------------------?---------0---0----0-----?-
--0---------------------------??------------------0-0--0------

Nematomorpha 0110111111000001110-100111000101110100000-010101-
000000000--00000000000011200--0-0000--------0----00-1000-00000000000--0-0-0-0--000---
-000-----00-1010--00000---------------0----------------------------------?--0------0---0----0-----?-
--0---------------------------??------------------0-0--0------

Priapulida 0110111111000001110-100111000101111100000-011111-
000000000--00000000000011200--0-0000--------0----00-1000-00000000000--0-0-0-0--000---
-000-----00-1010--00000---------------0----------------------------------?--0------0---0----0-----?-
--0---------------------------??------------------0-0--0------

Gastrotricha 0110111111000001110-10011110010100-000000-010000-
000000000--00000000000011200--0-0000--------0----00-1000-00000000000--0-0-0-0--000---
-000-----00-1000--00000---------------0----------------------------------?---------0---0----0-----?-
--0---------------------------??------------------0-0--0------

Tardigrada 0110111111000001110-10011100010101110101 01?10001-
0000000000000000000000011200--0-0000--------0----00-1000-00000000000--0-0-0-0--000--
--000-----00-1000--00000---------------0----------------------------------?---------0---0----0-----
?--0---------------------------??------------------0-0--0----
--

Kinorhyncha 0110111111000001110-100111000101?10100000-111111-
000000000--00000000000011200--0-0000--------0----00-1000-00000000000--0-0-0-0--000---
-000-----00-1000--00000---------------0----------------------------------?---------0---0----0-----?-
--0---------------------------??------------------0-0--0------

Loricifera 0110111111000001110-100111000101?10100000-011111-
000000000--00000000000011200--0-0000--------0----00-1000-00000000000--0-0-0-0--000---

-000-----00-1010--00000---------------0----------------------------------?--0------0---0----0-----?-
---0-------------------------??-----------------0-0--0------

Gnathifera 0110111111000001110-10011110010100-000000-010000-000000100--00000000000011200--0-0000--------0----00-1000-00000000000--0-0-0-0--000----000-----00-1000--00000---------------0----------------------------------?---------0---0----0-----?-
---0-------------------------??-----------------0-0--0------

Xenacoelomorpha 0100?1?11(0 1)000001111-1111110000-0-0-000000-0?0000-0000000-0--00000000000011200--0-0000--------0----00-1000-00000000000--0-0-0-0--000----000-----00-1000--00000---------------0----------------------------------?---------0---0----0-----?-
---0-------------------------??-----------------0-0--0------

Platyhelminthes 0110111111000001110-1001111000-000-000000-010000-100000000--00000000000011200--0-0000-------?0----00-1000-00000000000--0-0-0-0--000----000-----00-1010--00000---------------0----------------------------------?--0------0---0----0-----
?---0-------------------------??-----------------0-0--0----
--

Auroralumina
??????????0??0????????????????????????00?0???????????????????????????????????1??0?????0
???????????????0??0?????????????0????????????????????0-----
????????????????????????????1?1????10???????????1100-0--1-?10-1?????????????1210?0-
???????????????????00???????0???5???
??

Eoconularia
??????????0??0???????????????00?????00?0?????????0????????????????????????1?30?????
???????????????0??0?????????????0????????????????????0-----
??????????????????????????????1110?101??110-0200110101010100?0111???0????------110-?-
--
?11???1???
???????????????????????????????????????

Conularia

??????????0??0???????????????00?????00?0?????????0????????????????????????1?30?????

????????????????0??0?????????????0???????????????????0-----

??????????????????????????????1????101??110-02101101010110111???0????------110-?-

--

?11???

???????????????????????????????????

Carinachites

??????????0??0???????????????00?????00?0??????????????????????????????????1?30?????

????????????????0??0?????????????0???????????????????0-----

??????????????????????????????10--?10???110-11011110-0-11-100-1??????????????100-?--

-

?11???

???????????????????????????????????

Olivoiides ??????????0??0???????????????00?-

???00?0?????????0????????????????????????1?40?????0???????????????0??0?????????????

0????????????????????0-----?????0????????????????????????1101?10???1110---010-00-01-

010101100??--0------1?0-?---

?11??????????????????????1?????????????????????1000????1?1???????????????????????????

????????????????????????????????????

Quadrapyrgites ??????????0??0???????????????00?-

???00?0?????????0?????????????????????????1?30?????0???????????????0??0?????????????

0????????????????????0-----?????0????????????????????????1101?10???1110---010-00-01-

010101?????--0------1?0-?---

?11??????????????????????1??????????????????????????????1?????????????????????????????

???????????????????????????????????

Pseudooides ??????????0??0???????????????00?-

???00?0?????????0????????????????????????1?10?????0???????????????0??0?????????????

0?????????????????????0-----?????0?????????????????????????1????10???110-020010-1?-?1-0?0111?????--0------1?0-?---

?????????????????????????1?????????????????????????????0???

Cambrocotonus

??????????0??0????????????????0??????00?0?????????0?????????????????????????1?00?????0???????????????0??0??????????????0?????????????????????0-----

??????????????????????????????0-------------------------------

??????????????1010?11??11??10???

Nematostella 0110011111000001101-1001010010-0-0-000000-00000010000000-0--000000000000011000-110001010000--0----00-1000-00000000000--0-0-0-0--000----000-----00-101111000110-0001000000101?0-----------------------------------01--1110110110-1---111-1001010021114000011l-1001103001?0100110001-----000010---------------------------??-------------------00-0--0-01001

Anemonia 0110011111000001101-1001010010-0-0-000000-00000010000000-0--000000000000011050-110001010000--0----00-1000-00000000000--0-0-0-0--000----000-----00-101111000110-0001001000101?0-----------------------------------01--1110110120-1---111-1001001021114000011l-10011051011010111011l-----000010---------------------------??-------------------10-0--0-01001

Aiptasia 0110011111000001101-1001010010-0-0-000000-00000010000000-0--000000000000011050-110001010000--0----00-1000-00000000000--0-0-0-0--000----000-----00-101111000110-0001000000101?0-----------------------------------01--1110110120-1---111-1001001021114001011l-100110501??1101110011-----000010----------------------------??-------------------10-0--0-01001

Metridium 0110011111000001101-1001010010-0-0-000000-00000010000000-0--000000000000011050-110001010000--0----00-1000-00000000000--0-0-0-0--000----000-----00-101111000110-0001000000101?0-----------------------------------01--

1110110120-1---111-10010010211140010111-111110501011101110011-----000010----------

------------------??-------------------00-0--0-01001

Antipathes 0110011111000001101-1001010010-0-0-000000-

00000010000000-0--000000000000011050-110001010000--0----00-1000-00000000000--0-

0-0-0--000----000-----00-1011110001?0-0001001000101?0----------------------------------

0100101011010101100011-10010000101130000?12-000100500??0--0000000-----000010----

------------------------??-------------------0100--0-01001

Ceriantharia 0110?11111000001101-1001010010-0-0-000000-

00000010000000-0--000000000000011000-110001010000--0----00-1000-00000000000--0-

0-0-0--000----000-----00-101111000110-0001001000111?0----------------------------------01--

1010110120-1---111-10010?00201020100011-0001105001?0110000001-----000000-----------

-----------------??-------------------00-0--0-01001

Corynactis 0110011111000001101-1001010010-0-0-000000-

00000010000000-0--000000000000011050-110001010000--0----00-1000-00000000000--0-

0-0-0--000----000-----00-101111000110-0001001000101?0----------------------------------01--

1110110100-1---111-10010010210-40000-12-110111500??0110000010-----000010------------

----------------??-------------------10-0--0-01001

Montastraea 0110011111000001101-1001010010-0-0-000000-

00000010000000-0--000000000000011050-110001010000--0----00-1000-00000000000--0-

0-0-0--000----000-----00-101111000110-0001001000101?0----------------------------------

0100111011010101100111-10010010210-40000-12-100111500??0--0000000-----000010-----

-----------------------??-------------------1110110-01001

Porites 0110011111000001101-1001010010-0-0-000000-00000010000000-

0--000000000000011050-110001010000--0----00-1000-00000000000--0-0-0-0--000----000--

---00-101111000110-0001001000101?0----------------------------------

0100111011010101100111-10010010210-40000-12-100111300??0--0000000-----000010-----

-----------------------??-------------------1110110-01001

Acropora 0110011111000001101-1001010010-0-0-000000-00000010000000-0--00000000000011050-110001010000--0----00-1000-00000000000--0-0-0-0--000----000-----00-101111000110-0001001000101?0----------------------------------010011101101010101100111-10010010210-40000-12-100111200??0--0000000-----000010-----------------------------??-------------------1110110-01001

Parazoanthus 0110011111000001101-1001010010-0-0-000000-00000010000000-0--00000000000011000-110001010000--0----00-1000-00000000000--0-0-0-0--000----000-----00-101111000110-0001001000101?0----------------------------------010010101101010101100111-10010110221110000011-100110500??0100011001-----000010---------------------------??-------------------10-0--0-01001

Anthomastus 0110011111000001101-1001010010-0-0-000000-00000010000000-0--00000000000011000-110001011000--0----00-1000-00000000000--0-0-0-0--000----000-----00-101111000110-0000001000001?0----------------------------------010010101101010101100011-11011-00001000000010-000100100??0??0000001-----000010---------------------------??-------------------00-1--1101001

Keratoisidinae 0110011111000001101-1001010010-0-0-000000-00000010000000-0--00000000000011000-110001011000--0----00-1000-00000000000--0-0-0-0--000----000-----00-101111000110-0000001000001?0----------------------------------010010101101010101100011-11011-00001000000010-000100100??0??0000001-----000010---------------------------??-------------------00-1--1101001

Nephthyigorgia 0110011111000001101-1001010010-0-0-000000-00000010000000-0--00000000000011000-110001011000--0----00-1000-00000000000--0-0-0-0--000----000-----00-101111000110-0000001000001?0----------------------------------010010101101010101100011-11011-00001000000010-000100100??0??0000001-----000010---------------------------??-------------------00-1--1111001

Leptogorgia 0110011111000001101-1001010010-0-0-000000-00000010000000-0--00000000000011000-110001011000--0----00-1000-00000000000--0-0-0-0--000----000-----00-101111000110-0000001000001?0----------------------------------

01001010110101011000111-11011-00001000000010-000100100??0??0000001-----000010---
-------------------------??-------------------00-1--1111001

Scleronepthya 0110011111000001101-1001010010-0-0-000000-00000010000000-0--000000000000011000-110001011000--0----00-1000-00000000000--0-0-0-0--000----000-----00-101111000110-0000001000001?0----------------------------------
01001010110101011000111-11011-00001000000010-000100100??0??0000001-----000010---
-------------------------??-------------------00-1--1101001

Virgularia 0110011111000001101-1001010010-0-0-000000-00000010000000-0--000000000000011000-110001011000--0----00-1100-00000000000--0-0-0-0--000----000-----00-101111000110-0000001000001?0----------------------------------
01001010110101011101011-11011-00001000000010-000100100??0??0000001-----000010---
-------------------------??-------------------00-1--0-01001

Haliclystus 0110011111000001101-1001010010-0-0-000000-00000010000000-0--000000000000011030-100001010000--0----00-1-00-00000000000--0-0-0-0--000----000-----00-10111?1??0000010000100000011---00-010-----------------------11-12--1001000110-0---01111110--00--0-?0001---00-00-0100--0??000000-0----
00?121101010110000-110001?-10000010-01100010000000000000100-0--0001100

Lucernaria 0110011111000001101-1001010010-0-0-000000-00000010000000-0--000000000000011030-100001010000--0----00-1-00-00000000000--0-0-0-0--000----000-----00-10111?1??0000010000100000011---00-010-----------------------11-12--1001000110-0---01111110--00--0-?0001---00-00-0100--0??000000-0----
00?121101110110000-110001?-10000010-01100010000000000000100-0--0001100

Calvadosia 0110011111000001101-1001010010-0-0-000000-00000010000000-0--000000000000011030-100001010000--0----00-1-00-00000000000--0-0-0-0--000----000-----00-10111?1??0000010000100000011---00-010-----------------------11-12--1001000110-0---01111110--00--0-?0001---00-00-0100--0??000000-0----
00?121111101000000-110001?-10000010-011000100-00000000100-0--0001100

Alatina 0110011111000001101-1001010010-0-0-000000-00000010000000-0--000000000000011010-100001010000--0----00-1-00-00000000000--0-0-0-0--000----000-----00-1011101--0001110100100000011---10-011----------------------11-1?--1010??0100-1---0101001----------1001---00-00-0100--0??000000-0----0111210-00---0011101010010111001011002100011000100001100-0-0--0001100

Chironex 0110011111000001101-1001010010-0-0-000000-00000010000000-0--000000000000011010-100001010000--0----00-1000-00000000000--0-0-0-0--000----000-----00-1011101--0001110100100000011---10-011----------------------10(0 1)??--1010??0100-1---0101001----------1001---00-00-0100--0??000000-0----0111210-00---001110101001011100101100210001100010000111 00-0--0001100

Morbakka 0110011111000001101-1001010010-0-0-000000-00000010000000-0--000000000000011010-100001010000--0----00-1000-00000000000--0-0-0-0--000----000-----00-1011101--0001110100100000011---10-011----------------------101??--1010??0100-1---0101001----------1001---00-00-0300--0??000000-0----0111210-00---0011101010010111001011002100011000100001110 0-0--0001100

Atolla 0110011111000001101-1001010010-0-0-000000-00000010000000-0--000000000000011030-100001010000--0----00-1000-00000000000--0-0-0-0--000----000-----00-1011101?000010100001000000???????????????-?-?????????????????10???--??????0?0???--?01111010--00--0-?1000---?0-0100500--0??000000-11??10011210-00---01100-1000110101000111000000011100100000000 0-0--0001100

Stephanoscyphistoma 0110011111000001101-1001010010-0-0-000000-00000010000000-0--000000000000011030-110001010000--0----00-1000-00000000000--0-0-0-0--000----000-----00-1011101000001010000100000001120110011110-000000-001-0-1001001001?--10????010(0 1)(0 3)1---01111010--00--0-?1000---00-0100500--0??000000-1????0011210-00---01100-10001101010001110000000111001000000000-0--0001100

Nausithoe 0110011111000001101-1001010010-0-0-000000-00000010000000-0--000000000000011030-110001010000--0----00-1000-00000000000--0-0-0-0--000----000-----00-1011101000001010000100000001120110011110-000000-001-0-

10010010010--10????010(0 1)31---01111010--00--0-?1000---00-0100500--0??000000-103?10011210-00---01100-10001101010001110000000111001000000000-0--0001100

Aurelia 0110011111000001101-1001010010-0-0-000000-00000010000000-0--0000000000000011030-110001010000--0----00-1000-00000000000--0-0-0-0--000----000-----00-1011101100001010000110000001---00-010----------------------10011--10??110100-1---01101010--00--0-?1000---00-00-0300--0??000000-111110011210-00---00100-10001101-11100110000000110001000000000-0--0001100

Cassiopea 0110011111000001101-1001010010-0-0-000000-00000010000000-0--0000000000000011030-110001010000--0----00-1000-00000000000--0-0-0-0--000----000-----00-1011101100001010000110000001---00-010----------------------1010?--10????0100-1---01101010--00--0-?1000---00-00-0500--0???00000-113110011210-00---00100-10001101-1110011000001?110001000000000-0--0001100

Rhizostoma 0110011111000001101-1001010010-0-0-000000-00000010000000-0--0000000000000011030-110001010000--0----00-1?00-00000000000--0-0-0-0--000----000-----00-1011101100001010000110000001---00-010----------------------10011--10??000100-1---01101010--00--0-?1000---00-00-0300--0??000000-111110011210-00---00100-100010-1-1110011000001-11--01000-00-00-0--0001100

Hydra 0110111111000001101-1001010010-0-0-000000-00000010000000-0--0000000000000011030-100001010000--0----00-1100-00000000000--0-0-0-0--000----000-----00-1001001010001111100100000001---22-201----------------------0--13---------100-1---110-------------?000----0-0100100--0??000000-0----000000--00---0----?----------??-----------------------00-0--0001010

Candelabrum 0110111111000001101-1001010010-0-0-000000-00000010000000-0--0000000000000011030-100001010000--0----00-1000-00000000000--0-0-0-0--000----000-----00-10010010100011111001000000010--0110110-------00-0------000-00--0300-------101010-0110-------------?000---00-00-0100--0??000000-0----000000--00---0---?----------??----------------------00-0--0001010

Hydractinia 0110111111000001101-1001010010-0-0-000000-00000010000000-0--000000000000011030-100001010000--0----00-1000-00000000000--0-0-0-0--000----000-----00-10110010100011111001000000010--0110110-------00-0------000-00--0300????110101110-1110--------------?000---00-0100300--0??000000-0----0000010-00---0??00-0--0001010

Ectopleura 0110111111000001101-1001010010-0-0-000000-00000010000000-0--000000000000011030-100001010000--0----00-1000-00000000000--0-0-0-0--000----000-----00-10010010100011111001000000010--0110111010---000-0---1--000-00--0300-------101110-01100-------------?000---00-0100500--0??000000-0----0000010-00---000-110001011--1100000-10000011?001000000000-0--0001010

Clytia 0110111111000001101-1001010010-0-0-000000-00000010000000-0--000000000000011030-100001010000--0----00-1100-00000000000--0-0-0-0--000----000-----00-10110010100011101101000000010--0100111011---000-0---1--010-00--030010??110101110-11100-------------1000---00-0100500--0??000000-0----0000010-00---000-1100010110-1100011100000011000?000000000-0--0001010

Obelia 0110111111000001101-1001010010-0-0-000000-00000010000000-0--000000000000011030-100001010000--0----00-1100-00000000000--0-0-0-0--000----000-----00-10110010100011101101000000010--0100111011---000-0---1--010-00--030010??110101110-11100-------------1000---00-0100500--0??000000-0----0000010-00---000-1100010110-1100011100000011000?000000000-0--0001010

Physalia 0110111111000001101-1001010010-0-0-000000-00000010000000-0--000000000000011030-100001010000--0----00-1000-00000000000--0-0-0-0--000----000-----00-1011001010001111100101100001---02-011----------------------0--0?0110??110101200-11100-------------0000----0-0??0?00--0??000000-0----0000010-00---000-110001001--1100000-00000-11--00000-00-00-0--0001010

Craseoa 0110111111000001101-1001010010-0-0-000000-00000010000000-0--000000000000011030-100001010000--0----00-1000-00000000000--0-0-0-0--000----000-----00-1011001010001111100101100001---02-011----------------------0--0?1110??110101200-

11100-------------0000----0-0??0?00--0??000000-0----0000010-00---000-110001001--1100000-00000-11--00000-00-00-0--0001010

Abylopsis 0110111111000001101-1001010010-0-0-000000-00000010000000-0--000000000000011030-100001010000--0----00-1000-00000000000--0-0-0-0--000----000-----00-1011001010001111100101100001---02-011-----------------------0--0?1110??110101200-11100-------------0000----0-0??0?00--0??000000-0----0000010-00---000-110001001--1100000-00000-11--00000-00-00-0--0001010

Agalma 0110111111000001101-1001010010-0-0-000000-00000010000000-0--000000000000011030-100001010000--0----00-1000-00000000000--0-0-0-0--000----000-----00-1011001010001111100101100001---02-011-----------------------0--0?1010??110101200-11100-------------0000----0-0??0?00--0??000000-0----0000010-00---000-110001001--1100000-00000-11--00000-00-00-0--0001010

Nanomia 0110111111000001101-1001010010-0-0-000000-00000010000000-0--000000000000011030-100001010000--0----00-1000-00000000000--0-0-0-0--000----000-----00-1011001010001111100101100001---02-011-----------------------0--0?1010??110101200-11100-------------0000----0-0??0?00--0??000000-0----0000010-00---000-110001001--1100000-00000-11--00000-00-00-0--0001010

Aeginia 0110111111000001101-1001010010-0-0-000000-00000010000000-0--000000000000011030-100001010000--0----00-1000-00000000000--0-0-0-0--000----000-----00-10110010100010000001100000-------------------------------------030010??0011-------01-0---1000-10-00---000-??0001010?-010000100000010?1010100000000-0--0001010

Halitrephes 0110111111000001101-1001010010-0-0-000000-00000010000000-0--000000000000011030-100001010000--0----00-1000-00000000000--0-0-0-0--000----000-----00-10110010100011100001000000--------------------------------------30010??0000-------01-0---1000-10-00---000-1100010100-11000110000100111000000000000-0--0001010;

Begin MrBayes;

```
lset rates = gamma coding=informative;

mcmc ngen=100000000 stoprule=yes stopval=0.01 app=n;

sumt contype=h ;

sump;
```

Supplementary references:

1 Hofmann, H., Fritz, W. & Narbonne, G. Ediacaran (Precambrian) fossils from the Wernecke Mountains, northwestern Canada. *Science* **221**, 455-457 (1983).

2 Antcliffe, J. B. & Brasier, M. D. *Charnia* and sea pens are poles apart. *Journal of the Geological Society* **164**, 49-51 (2007).

3 Butterfield, N. J. Constructional and functional morphology of Ediacaran rangeomorphs. *Geological Magazine* (2020).

4 Dunn, F. S. *et al.* The developmental biology of *Charnia* and the eumetazoan affinity of the Ediacaran rangeomorphs. *Science advances* **7**, eabe0291 (2021).

5 Liu, A. G. & McIlroy, D. Horizontal surface traces from the Fermeuse Formation, Ferryland (Newfoundland, Canada), and their place within the late Ediacaran ichnological revolution. *Geological Association of Canada Miscellaneous Publications* **9**, 141-156 (2015).

6 Menon, L. R., McIlroy, D. & Brasier, M. D. Evidence for Cnidaria-like behavior in ca. 560 Ma Ediacaran *Aspidella*. *Geology* **41**, 895-898 (2013).

7 Warren, L. *et al.* Microbially induced pseudotraces from a Pantanal soda lake, Brazil: Alternative interpretations for Ediacaran simple trails and their limits. *Geology* **48**, 857-861 (2020).

8 Zhao, Y. *et al.* Cambrian sessile, suspension feeding stem-group ctenophores and evolution of the comb jelly body plan. *Current Biology* **29**, 1112-1125 (2019).

9 Wan, B. *et al.* Systematic description of putative animal fossils from the early Ediacaran Lantian Formation of South China. *Palaeontology* **59**, 515-532 (2016).

10 Xunlai, Y., Jun, L. & Ruiji, C. A diverse metaphyte assemblage from the Neoproterozoic black shales of South China. *Lethaia* **32**, 143-155 (1999).

11 Wan, B. *et al.* A tale of three taphonomic modes: the Ediacaran fossil *Flabellophyton* preserved in limestone, black shale, and sandstone. *Gondwana Research* (2020).

12 Van Iten, H., Leme, J. d. M., Marques, A. C. & Simões, M. G. Alternative interpretations of some earliest Ediacaran fossils from China. *Acta Palaeontologica Polonica* **58**, 111-113 (2013).

13 Ivantsov, A. Y. & Fedonkin, M. A. Conulariid–like fossil from the Vendian of Russia: a metazoan clade across the Proterozoic/Palaeozoic boundary. *Palaeontology* **45**, 1219-1229 (2002).

14 Leme, J. *et al.* A new Ediacaran conulariid from the Tamengo Formation, Corumbá Group, Brazil, and the deep Precambrian evolutionary history of cnidarians. *The*

Neoproterozoic Paraguay Fold Belt (Brazil): glaciations, iron–manganese formation and biota, 15 (2013).

15 Ivantsov, A. Y., Vickers-Rich, P., Zakrevskaya, M. & Hall, M. Conical Thecae of Precambrian Macroorganisms. *Paleontological Journal* **53**, 1134-1146 (2019).

16 Selly, T. *et al.* A new cloudinid fossil assemblage from the terminal Ediacaran of Nevada, USA. *Journal of Systematic Palaeontology* **18**, 357-379 (2020).

17 Park, T.-Y. S. *et al.* Enduring evolutionary embellishment of cloudinids in the Cambrian. *Royal Society open science* **8**, 210829 (2021).

18 Cortijo, I., Mus, M. M., Jensen, S. & Palacios, T. A new species of *Cloudina* from the terminal Ediacaran of Spain. *Precambrian Research* **176**, 1-10 (2010).

19 Germs, G. J. New shelly fossils from Nama Group, south west Africa. *American Journal of Science* **272**, 752-761 (1972).

20 Adorno, R. R. *et al. Cloudina lucianoi* (Beurlen & Sommer, 1957), Tamengo Formation, Ediacaran, Brazil: taxonomy, analysis of stratigraphic distribution and biostratigraphy. *Precambrian Research* **301**, 19-35 (2017).

21 Cai, Y., Schiffbauer, J. D., Hua, H. & Xiao, S. Morphology and paleoecology of the late Ediacaran tubular fossil *Conotubus hemiannulatus* from the Gaojiashan Lagerstätte of southern Shaanxi Province, South China. *Precambrian Research* **191**, 46-57 (2011).

22 Han, J. *et al.* A *Cloudina*-like fossil with evidence of asexual reproduction from the lowest Cambrian, South China. *Geological Magazine* **154**, 1294-1305 (2017).

23 Cai, Y., Cortijo, I., Schiffbauer, J. D. & Hua, H. Taxonomy of the late Ediacaran index fossil *Cloudina* and a new similar taxon from South China. *Precambrian Research* **298**, 146-156 (2017).

24 Min, X., Hua, H., Cai, Y. & Sun, B. Asexual reproduction of tubular fossils in the terminal Neoproterozoic Dengying Formation, South China. *Precambrian Research* **322**, 18-23 (2019).

25 Yang, B. *et al.* Transitional Ediacaran–Cambrian small skeletal fossil assemblages from South China and Kazakhstan: Implications for chronostratigraphy and metazoan evolution. *Precambrian Research* **285**, 202-215 (2016).

26 Carbone, C. A., Narbonne, G. M., Macdonald, F. A. & Boag, T. H. New Ediacaran fossils from the uppermost Blueflower Formation, northwest Canada: disentangling biostratigraphy and paleoecology. *Journal of Paleontology* **89**, 281-291 (2015).

27 Walde, D. H.-G., Weber, B., Erdtmann, B.-D. & Steiner, M. Taphonomy of *Corumbella werneri* from the Ediacaran of Brazil: sinotubulitid tube or conulariid test? *Alcheringa.* **43**, 335-350 (2019).

28 Droser, M. L. & Gehling, J. G. Synchronous aggregate growth in an abundant new Ediacaran tubular organism. *Science* **319**, 1660-1662 (2008).

29 Surprenant, R. L., Gehling, J. G. & Droser, M. L. Biological and Ecological Insights from the Preservational Variability *of Funisia dorothea*, Ediacara Member, South Australia. *Palaios* **35**, 359-376 (2020).

30 Cai, Y., Hua, H. & Zhang, X. Tube construction and life mode of the late Ediacaran tubular fossil *Gaojiashania cyclus* from the Gaojiashan Lagerstätte. *Precambrian Research* **224**, 255-267 (2013).

31 Ebbestad, J. O. R. *et al.* Distribution and correlation of *Sabellidites cambriensis* (Annelida?) in the basal Cambrian on Baltica. *Geological Magazine*, 1-22 (2021).

32 Wang, X., Zhang, X., Zhang, Y., Cui, L. & Li, L. New materials reveal *Shaanxilithes* as a *Cloudina*-like organism of the late Ediacaran. *Precambrian Research* **362**, 106277 (2021).

33 Cai, Y., Xiao, S., Hua, H. & Yuan, X. New material of the biomineralizing tubular fossil *Sinotubulites* from the late Ediacaran Dengying Formation, South China. *Precambrian Research* **261**, 12-24 (2015).

34 Sappenfield, A., Droser, M. L. & Gehling, J. G. Problematica, trace fossils, and tubes within the Ediacara Member (South Australia): Redefining the Ediacaran trace fossil record one tube at a time. *Journal of Paleontology* **85**, 256-265 (2011).

35 Chen, Z. *et al.* New Ediacara fossils preserved in marine limestone and their ecological implications. *Scientific Reports* **4**, 1-10 (2014).
36 Dong, X.-P. *et al.* Embryos, polyps and medusae of the Early Cambrian scyphozoan *Olivooides*. *Proceedings of the Royal Society B: Biological Sciences* **280**, 20130071 (2013).
37 Morris, S. C. & Menge, C. Carinachitids, hexangulaconulariids, and Punctatus: problematic metazoans from the Early Cambrian of South China. *Journal of Paleontology*, 384-406 (1992).
38 Guo, J. *et al.* A fourteen-faced hexangulaconulariid from the early Cambrian (Stage 2) Yanjiahe Formation, South China. *Journal of Paleontology* **94**, 45-55 (2020).
39 Duan, B. *et al.* The early Cambrian fossil embryo *Pseudooides* is a direct-developing cnidarian, not an early ecdysozoan. *Proceedings of the Royal Society B: Biological Sciences.* **284**, 20172188 (2017).
40 Liu, Y. *et al. Quadrapyrgites* from the lower Cambrian of South China: growth pattern, post-embryonic development, and affinity. *Chinese Science Bulletin* **59**, 4086-4095 (2014).
41 De Moraes Leme, J., Guimarães Simões, M., Carlos Marques, A & Van Iten, H. Cladistic analysis of the suborder Conulariina Miller and Gurley, 1896 (Cnidaria, Scyphozoa; Vendian–Triassic). *Palaeontology* **51**, 649-662 (2008).
42 Van Iten, H., Muir, L. A., Botting, J. P., Zhang, Y. & Lin, J.-P. Conulariids and *Sphenothallus* (Cnidaria, Medusozoa) from the Tonggao Formation (Lower Ordovician, China). *Bulletin of Geosciences* **88**, 713-722 (2013).
43 Van Iten, H. *et al.* Origin and early diversification of the phylum Cnidaria Verrill: major developments in the analysis of the taxon's Proterozoic-Cambrian history. *Palaeontology* **57**, 677-690, doi:10.1111/pala.12116 (2014).
44 Hughes, N. C., Gunderson, G. O. & Weedon, M. J. Late Cambrian conulariids from Wisconsin and Minnesota. *Journal of Paleontology* **74**, 828-838 (2000).
45 Yunhuan, L. *et al.* Two new species of protoconulariids from the early Cambrian in South Shaanxi, China. *Acta Micropalaeontologica Sinica* **22**, 311-321 (2005).
46 Han, J. *et al. Olivooides*-like tube aperture in early Cambrian carinachitids (Medusozoa, Cnidaria). *Journal of Paleontology* **92**, 3-13 (2018).
47 刘云焕 *et al.* 陕西宁强原始锥石类五辐对称新属种的发现. *微体古生物学报* **28**, 244-249 (2011).
48 Dzik, J., Baliński, A. & Sun, Y. The origin of tetraradial symmetry in cnidarians. *Lethaia* **50**, 306-321 (2017).
49 Zhu, M.-y., Van Iten, H., Cox, R. S., Zhao, Y.-l. & Erdtmann, B.-D. Occurrence of *Byronia* Matthew and *Sphenothallus* Hall in the Lower Cambrian of China. *PalZ* **74**, 227-238 (2000).
50 Junyuan, C. & Qingqing, P. An Early Cambrian problematic organism Anabarites and its possible affinity. *Gu Sheng wu xue bao= Acta Palaeontologica Sinica* **44**, 57-65 (2005).
51 Shao, T. *et al.* Research on the symmetrical evolution of *Anabarites* from the Cambrian Xixiang Biota in southern Shaanxi Province. *Acta Micropalaeontologica Sinica*, 04 (2015).
52 Skovsted, C. B. & Peel, J. S. Hyolithellus in life position from the lower Cambrian of North Greenland. *Journal of paleontology* **85**, 37-47 (2011).
53 Peel, J. A problematic cnidarian (Cambroctoconus; Octocorallia?) from the Cambrian (Series 2–3) of Laurentia. *Journal of Paleontology* **91**, 871-882 (2017).
54 Geyer, G. *et al.* A remarkable Amgan (Middle Cambrian, Stage 5) fauna from the Sauk Tanga, Madygen region, Kyrgyzstan. *Bulletin of Geosciences* **89**, 375-400 (2014).
55 Park, T. Y. S. *et al.* Cambrian Stem-group Cnidarians with a New Species from the Cambrian Series 3 of the Taebaeksan Basin, Korea. *Acta Geologica Sinica-English Edition* **90**, 827-837 (2016).

56 Park, T.Y. S. *et al.* A stem-group cnidarian described from the mid-Cambrian of China and its significance for cnidarian evolution. *Nature Communications* **2**, 1-6 (2011).
57 Korde, K. Hydroconozoa-a new class of Coelenterates. *International Geology Review* **6**, 2229-2234 (1964).
58 Zhuravlev, A. Y., Debrenne, F. & Lafuste, J. Early Cambrian microstructural diversification of Cnidaria. *Courier Forschungsinstitut Senckenberg* **164**, 365-372 (1993).
59 Jell, P. A. & Jell, J. S. Early Middle Cambrian corals from western New South Wales. *Alcheringa* **1**, 181-195 (1976).
60 Peel, J. S. The coral *Cothonion* from the lower Cambrian of North Greenland. *Alcheringa* **35**, 405-411 (2011).
61 Fuller, M. & Jenkins, R. Reef corals from the lower Cambrian of the Flinders Ranges, South Australia. *Palaeontology* **50**, 961-980 (2007).
62 Scrutton, C. T. The Palaeozoic corals, I: origins and relationships. *Proceedings of the Yorkshire Geological Society* **51**, 177-208 (1997).
63 Murdock, D. J. The 'biomineralization toolkit'and the origin of animal skeletons. *Biological Reviews* **95**, 1372-1392 (2020).
64 O'Reilly, J. E. *et al.* Bayesian methods outperform parsimony but at the expense of precision in the estimation of phylogeny from discrete morphological data. *Biology Letters* **12**, 20160081 (2016).
65 Puttick, M. N., O'Reilly, J. E., Pisani, D. & Donoghue, P. C. Probabilistic methods outperform parsimony in the phylogenetic analysis of data simulated without a probabilistic model. *Palaeontology* **62**, 1-17 (2019).
66 Brazeau, M. D. Problematic character coding methods in morphology and their effects. *Biological Journal of the Linnean Society* **104**, 489-498 (2011).
67 Toshino, S. *et al.* Development and polyp formation of the giant box jellyfish *Morbakka virulenta* (Kishinouye, 1910)(Cnidaria: Cubozoa) collected from the Seto Inland Sea, western Japan. *Plankton and Benthos Research* **8**, 1-8 (2013).
68 Sendino, C. & Bochmann, M. M. An exceptionally preserved conulariid from Ordovician erratics of Northern European Lowlands. *PalZ*, 1-14 (2021).
69 Jerre, F. Anatomy and phylogenetic significance of *Eoconularia loculata*, a conulariid from the Silurian of Gotland. *Lethaia* **27**, 97-109 (1994).
70 Zapata, F. *et al.* Phylogenomic analyses support traditional relationships within Cnidaria. *bioRxiv*, 017632 (2015).
71 Kayal, E. *et al.* Phylogenomics provides a robust topology of the major cnidarian lineages and insights on the origins of key organismal traits. *BMC Evolutionary Biology* **18**, 68 (2018).
72 Whelan, N. V. *et al.* Ctenophore relationships and their placement as the sister group to all other animals. *Nature ecology & evolution* **1**, 1737 (2017).
73 Leys, S. P., Nichols, S. A. & Adams, E. D. Epithelia and integration in sponges. *Integrative and comparative biology* **49**, 167-177 (2009).
74 Nielsen, C. *Animal evolution: interrelationships of the living phyla*. (Oxford University Press on Demand, 2012).
75 Riesgo, A., Maldonado, M., López-Legentil, S. & Giribet, G. A proposal for the evolution of cathepsin and silicatein in sponges. *Journal of molecular evolution* **80**, 278-291 (2015).
76 Mueller, J. F. Some observations on the structure of hydra, with particular reference to the muscular system. *Transactions of the American Microscopical Society* **69**, 133-147 (1950).
77 Chia, F. S., Amerongen, H. M. & Peteya, D. J. Ultrastructure of the neuromuscular system of the polyp of *Aurelia aurita* L., 1758 (Cnidaria, Scyphozoa). *Journal of morphology* **180**, 69-79 (1984).

78 Chapman, D. Microanatomy of the cubopolyp, *Tripedalia cystophora* (Class Cubozoa). *Helgolaender wissenschaftliche meeresuntersuchungen* **31**, 128-168 (1978).
79 Kikinger, R. & von Salvini-Plawen, L. Development from polyp to stauromedusa in *Stylocoronella* (Cnidaria: Scyphozoa). *Journal of the Marine Biological Association of the United Kingdom* **75**, 899-912 (1995).
80 Chapman, D. Behavior and flagellar currents in coronate polyps (Scyphozoa) and comparisons with semaeostome polyps. *Helgoländer wissenschaftliche Meeresuntersuchungen* **25**, 214-227 (1973).
81 Komai, T. On *Stephanoscyphus* and *Nausithoe*. *Memoirs of the College of Science, Kyoto Imperial University. Ser. B* **10**, 289-339 (1935).
82 Stampar, S. N. *et al.* Ceriantharia in current systematics: life cycles, morphology and genetics. *The Cnidaria, past, present and future*, 61-72 (2016).
83 Hyman, L. H. Protozoa through ctenophora. (New York: McGraw Hill, 1940).
84 Miranda, L. S., Collins, A. G. & Marques, A. C. Molecules clarify a cnidarian life cycle–the "hydrozoan" *Microhydrula limopsicola* is an early life stage of the staurozoan *Haliclystus antarcticus*. *PLoS One* **5**, e10182 (2010).
85 Fritz, A. E., Ikmi, A., Seidel, C., Paulson, A. and Gibson, M.C. Mechanisms of tentacle morphogenesis in the sea anemone *Nematostella vectensis*. *Development* **140**, 2212-2223 (2013).
86 Mendoza-Becerril, M. A. *et al.* An evolutionary comparative analysis of the medusozoan (Cnidaria) exoskeleton. *Zoological Journal of the Linnean Society* **178**, 206-225 (2016).
87 Morandini, A. Identification of coronate polyps from the Arctic Ocean: *Nausithoe werneri* Jarms, 1990 (Cnidaria, Scyphozoa, Coronatae), with notes on its biology. *Steenstrupia* **32**, 69-77 (2010).
88 Chapman, D. & Werner, B. Structure of a solitary and a colonial species of *Stephanoscyphus* (Scyphozoa, Coronatae) with observations on periderm repair. *Helgoländer wissenschaftliche Meeresuntersuchungen* **23**, 393-421 (1972).
89 Werner, B. Contribution to the evolution of the genus *Stephanoscyphus* (Scyphozoa Coronatae) and the ecology and regeneration qualities of *Stephanoscyphys racemosus* Komai. *Departmental Bulletin Paper (Kyoto University)* (1970).
90 Toshino, S. *et al.* Monodisc strobilation in Japanese giant box jellyfish Morbakka virulenta (Kishinouye, 1910): a strong implication of phylogenetic similarity between Cubozoa and Scyphozoa. *Evolution and Development* **17**, 231-239 (2015).
91 Hewitt, C. L. & Goddard, J. H. A new species of large and highly contractile hydroid in the genus *Candelabrum* (Hydrozoa: Anthoathecatae) from southern Oregon, USA. *Canadian journal of zoology* **79**, 2280-2288 (2001).
92 Müller, W. A. a. L., T. Metamorphosis in the Cnidaria. *Canadian Journal of Zoology* **80**, 1755-1771 (2002).
93 Collins, A. G. Phylogeny of Medusozoa and the evolution of cnidarian life cycles. *Journal of Evolutionary Biology* **15**, 418-432 (2002).
94 Park, T. Y. *et al.* A stem-group cnidarian described from the mid-Cambrian of China and its significance for cnidarian evolution. *Nature Communications* **2**, 1-6 (2011).
95 Miranda, L. S. *et al*. Systematics of stalked jellyfishes (Cnidaria: Staurozoa). *PeerJ* **4**, e1951 (2016).
96 Ou, Q. *et al.* Three Cambrian fossils assembled into an extinct body plan of cnidarian affinity. *Proceedings of the National Academy of Sciences* **114**, 8835-8840 (2017).
97 Purcell, J. E. & Angel, D. L. *Jellyfish blooms: New problems and solutions*. Vol. 212 (Springer, 2015).
98 Miranda, L. S., García-Rodríguez, J., Collins, A.G., Morandini, A.C. and Marques, A.C. Evolution of the claustrum in Cnidaria: comparative anatomy reveals that it is exclusive to some species of Staurozoa and absent in Cubozoa. *Organisms Diversity & Evolution* **17**, 753-766 (2017).

99 Miranda, L. S., Collins, A.G., Hirano, Y.M., Mills, C.E. and Marques, A.C. Comparative internal anatomy of Staurozoa (Cnidaria), with functional and evolutionary inferences. *PeerJ* **4**, e2594 (2016).

100 Tibdall, J. G. Fine structural aspects of anthozoan desmocyte development (Phylum Cnidaria). *Tissue and Cell* **14**, 85-96 (1982).
