# Supplementary material for: A crown-group cnidarian from the Ediacaran of Charnwood Forest, UK
Source: Nat Ecol Evol. 2022 Jul 25;6(8):1095–104. doi: 10.1038/s41559-022-01807-x (PMC9349040; doi:10.1038/s41559-022-01807-x)
Supplement: Supplementary file 2 — Reporting Summary [file 41559_2022_1807_MOESM2_ESM.pdf]

nature portfolio

Corresponding author(s): Frances Dunn

Last updated by author(s): Oct 5, 2022

Reporting Summary

Nature Portfolio wishes to improve the reproducibility of the work that we publish. This form provides structure for consistency and transparency in reporting. For further information on Nature Portfolio policies, see our Editorial Policies and the Editorial Policy Checklist.

Statistics

For all statistical analyses, confirm that the following items are present in the figure legend, table legend, main text, or Methods section.

n/a	Confirmed	
☒	☐	The exact sample size (n) for each experimental group/condition, given as a discrete number and unit of measurement
☒	☐	A statement on whether measurements were taken from distinct samples or whether the same sample was measured repeatedly
☒	☐	The statistical test(s) used AND whether they are one- or two-sided<br>*Only common tests should be described solely by name; describe more complex techniques in the Methods section.*
☒	☐	A description of all covariates tested
☒	☐	A description of any assumptions or corrections, such as tests of normality and adjustment for multiple comparisons
☐	☒	A full description of the statistical parameters including central tendency (e.g. means) or other basic estimates (e.g. regression coefficient) AND variation (e.g. standard deviation) or associated estimates of uncertainty (e.g. confidence intervals)
☒	☐	For null hypothesis testing, the test statistic (e.g. F, t, r) with confidence intervals, effect sizes, degrees of freedom and P value noted<br>*Give P values as exact values whenever suitable.*
☐	☒	For Bayesian analysis, information on the choice of priors and Markov chain Monte Carlo settings
☒	☐	For hierarchical and complex designs, identification of the appropriate level for tests and full reporting of outcomes
☒	☐	Estimates of effect sizes (e.g. Cohen's d, Pearson's r), indicating how they were calculated

Our web collection on statistics for biologists contains articles on many of the points above.

Software and code

Policy information about availability of computer code

Data collection	Morphological phylogenetic data was compiled in Mesquite 3.6 (https://www.mesquiteproject.org/)
Data analysis	Disparity analyses were conducted in the open-source software 'R' (https://www.r-project.org), using the package dispRity (https://cran.r-project.org/web/packages/dispRity/dispRity.pdf). Morphological phylogenetic analyses were conducted in MrBayes 3.2.6 (open source) and the R package Claddis (https://cran.r-project.org/web/packages/Claddis/Claddis.pdf) was used for safe taxonomic reduction.

For manuscripts utilizing custom algorithms or software that are central to the research but not yet described in published literature, software must be made available to editors and reviewers. We strongly encourage code deposition in a community repository (e.g. GitHub). See the Nature Portfolio guidelines for submitting code & software for further information.

Data

Policy information about availability of data

All manuscripts must include a data availability statement. This statement should provide the following information, where applicable:

- Accession codes, unique identifiers, or web links for publicly available datasets
- A description of any restrictions on data availability
- For clinical datasets or third party data, please ensure that the statement adheres to our policy

Provide your data availability statement here.

Field-specific reporting

Please select the one below that is the best fit for your research. If you are not sure, read the appropriate sections before making your selection.

☐ Life sciences ☐ Behavioural & social sciences ☒ Ecological, evolutionary & environmental sciences

For a reference copy of the document with all sections, see nature.com/documents/nr-reporting-summary-flat.pdf

Ecological, evolutionary & environmental sciences study design

All studies must disclose on these points even when the disclosure is negative.

Study description	The material described is a fossil specimen from the Ediacaran of the UK, which have been imaged using low-angle lighting (RTI available also) which has been included in morphological disparity and morphological phylogenetic analyses.
Research sample	The fossil material is described from the Ediacaran of the UK (Charnwood Forest, ~560 million years old). The morphological phylogenetic matrix was based on a previously published matrix (Zhao et al. 2019) and the disparity matrix was based on a previously compiled dataset (Selly et al. 2020).
Sampling strategy	NA
Data collection	NA
Timing and spatial scale	NA
Data exclusions	Five taxa - Agalma, Keratoisidinae, Nephthyigorgia, Scleronepthya and Craseoa - were excluded from morphological phylogenetic analyses as they contained redundant information, following safe taxonomic reduction. Two further fossil taxa - Namacalathus, Eolympia - were excluded as they had previously been determined to have an extremely high (~95%) proportion of missing data (Namacalathus), the status of the genus was uncertain (Eolympia) (both following Zhao et al. 2019).
Reproducibility	NA
Randomization	Taxa were allocated into groups for disparity analyses and in plotting polyp size following previously described taxonomic groupings. Our fossil specimen was determined to belong to its own, new species because it displays a character combination not observed in other taxa included in our phylogenetic matrix or from the Ediacaran fossil record in general.
Blinding	Blinding of specimens is not relevant to morphological study of fossil specimens or phylogenetic analysis.

Did the study involve field work? ☒ Yes ☐ No

Field work, collection and transport

Field conditions	The specimen holotype remains in the field, but the plastotype was collected during a molding initiative of several outcrops in the Charnwood Forest area by British Geological Survey (BGS) staff. It is currently housed at the BGS Keyworth site and was the subject of study.
Location	The fossil is located in the Bed B assemblage of the Ediacaran of Charnwood Forest. The entire surface was molded and is now available at the British Geological Survey, Keyworth.
Access & import/export	The fossil was studied from casts and molds and so access and import/export are not relevant for this study.
Disturbance	NA

Reporting for specific materials, systems and methods

We require information from authors about some types of materials, experimental systems and methods used in many studies. Here, indicate whether each material, system or method listed is relevant to your study. If you are not sure if a list item applies to your research, read the appropriate section before selecting a response.

Materials & experimental systems

n/a	Involved in the study
☒	☐ Antibodies
☒	☐ Eukaryotic cell lines
☐	☒ Palaeontology and archaeology
☒	☐ Animals and other organisms
☒	☐ Human research participants
☒	☐ Clinical data
☒	☐ Dual use research of concern

Methods

n/a	Involved in the study
☒	☐ ChIP-seq
☒	☐ Flow cytometry
☒	☐ MRI-based neuroimaging

Palaeontology and Archaeology

Specimen provenance: The plastotype (GSM 106119) is housed at the British Geological Survey, Keyworth.

Specimen deposition: Fossil material is accessioned at the British Geological Survey, Keyworth.

Dating methods: NA

☐ Tick this box to confirm that the raw and calibrated dates are available in the paper or in Supplementary Information.

Ethics oversight: No ethical approval was needed as it is not required for the study of this fossil material or morphological phylogenetics.

Note that full information on the approval of the study protocol must also be provided in the manuscript.
